# A systematic review of sensors to combat crime and routes to further sensor development

**DOI:** 10.3389/fchem.2025.1568867

**Published:** 2025-06-12

**Authors:** Alice E. Cozens, Shane D. Johnson, Tung-Chun Lee

**Affiliations:** ^1^ DAWES Centre for Future Crime at UCL, Jill Dando Institute for Security and Crime Science, London, United Kingdom; ^2^ Institute for Materials Discovery, University College London (UCL), London, United Kingdom; ^3^ Department of Chemistry, University College London, London, United Kingdom

**Keywords:** systematic review, combating crime, sensor (or biosensor), point-of-care detection, low-cost sensors, illicit drug detection, body fluid analysis, food safety analysis

## Abstract

Forensic science plays an important part in crime reduction but faces many challenges. These include the validity, cost and complexity of current sensors used, and a reliance on trained professionals to conduct analyses. Recent advances in sensor technologies present a promising opportunity for rapid, decentralized, and cost-effective analysis by untrained individuals in the field. To date, a comprehensive systematic review covering sensing technologies and use cases has been lacking. This paper addresses that gap. After the initial screening of papers, 1,482 publications were included in the review, from which data on target analytes and sensing technologies were extracted. Given that law enforcement have limited resources, a second screening examined papers that focused on low-cost sensing devices published from 2020 onwards (N = 791). Overall, our review identified eleven key analyte categories that had been researched: illicit drugs, fingerprints, explosives, body fluids, food safety, poisons and toxins, pollutants, counterfeits and documentation, fire, gunshot, and others. Low-cost sensing technologies identified were categorised into electrochemical, colourimetric, immunoassay, luminescence and SERS. We review trends in the research reported, barriers to commercialisation and adoption, and review the use of these types of sensors by law enforcement agencies. Current sensors used by authorities face challenges of high costs, specificity issues, limited detection capabilities and complex sample preparation. Emerging research focuses on cost-effective printed electrodes and dual detection techniques to enhance analyte sensitivity and detection accuracy. Notably, body fluid analysis plays a crucial role in criminal cases, but current sensors suffer issues like false positives, DNA degradation, and high costs. Studies investigating eco-friendly materials and dual-detection approaches show promise in addressing these issues. Illicit drug analysis constitutes over one-third (36%) of included publications. In the UK, police rely on NIK tests and DrugWipe sensors for on-site drug detection, but challenges related to sensitivity, specificity, and confirmatory testing persist. Ongoing research explores dual detection methods, lateral flow immunoassays, and electro-chemiluminescent screening to enhance specificity and matrix tolerance. Future efforts should prioritise refining dual detection methods, reducing matrix interference, low-cost/eco-friendly materials and fostering collaboration between academia and law enforcement for effective implementation in these areas.

## 1 Introduction

Forensic science plays an important role in the detection and prosecution of crime. It draws on a variety of scientific disciplines and approaches (Bollella and Katz, 2020; Pereira de Oliveira et al., 2018), and can involve the analysis of biological fluids, illicit drug samples, gunshot residues and so on. While valuable, traditional technologies presently employed have problems to include damaging effects on DNA retrieval, unacceptable specificity, and the inability to perform concurrent analyses as part of a multiplex assay (Gooch et al., 2014). The utilisation of sensors, particularly biosensors, represents a considerable opportunity (Singh et al., 2014). Numerous forensic analysis methods use presumptive analysis (i.e., they are not confirmatory) and necessitate collected samples to be centrally analysed in a laboratory to meet evidential requirements. However, sensors may enable untrained persons to undertake rapid, decentralised (and less expensive) analyses of complex samples in the field (Bollella and Katz, 2020). Biosensors have been well developed in the healthcare sector, food and beverage industries and environmental monitoring fields (Chadha et al., 2022). However, even though their use may offer sensitive, user-friendly, selective and rapid on-site tools for analysis, in the context of forensic science, biosensors are relatively under-developed (Aydindogan et al., 2019; Geng et al., 2017; Yáñez-Sedeño et al., 2019).

In this article, we report a systematic review to take stock of the current uses of biosensors and to identify key areas for future research. We begin by explaining why sensors are important in this context and specify what defines a sensor and the key elements of the sensor we will be focussing on (target analytes and sensing technologies). We then discuss the approach taken to review the literature, detailing why a systematic approach was taken, present our findings, and then make suggestions for future work.

### 1.1 Investigating crime

To detect and investigate crime, illegal behaviour must be identified, and an understanding and reconstruction of the crime event possible. To do this, for many forms of crime, a coherent analysis of physical and other evidence is needed. This is termed forensic science (Chisum and Turvey, 2011). Recent legal and scientific advances have emphasised concerns over the validity of inferences and techniques used in forensic science. Validity is crucial as scientific inaccuracies have consequences for the criminal justice system, crime reduction and society. The reconstruction of a crime relies strongly on Locard’s Exchange Principle, that every contact leaves a trace (Locard, 1920). Other situations necessitate on-site detection at the time of a crime; for instance, roadside drug testing mandates that a trace of drug sample be identified immediately. Therefore, methods for analysis and identification of these traces are needed. Sensors are utilised already in many scenarios dealing with traces (target analytes) to detect crime. However, improved validity and increased capacity are needed. Sensors may help to deliver both.

### 1.2 Sensors

A sensor is defined as “*a device which detects and measures a physical property and records, indicates, or otherwise responds to it*” (Soanes and Stevenson, 2008). This can be anything from thermometers, accelerometer sensors to alcohol sensors.

#### 1.2.1 Biosensors

Due to recent advances in their development, one branch of sensors that is of particular importance to combating crime are biosensors (Parkhey and Mohan, 2018). Developments are mainly due to the utilisation of new nanomaterials and nanostructured devices (Harish et al., 2022), developments in microfabrication and miniaturisation technologies (Baracu and Gugoasa, 2021), new bio-recognition molecules (Bazin et al., 2017) and improved collaboration between life- and physical-scientists (Parkhey and Mohan, 2018).

Biosensor design incorporates three main stages. First, the biosensor must identify a specific analyte using a specific recognition component (bioreceptor–e.g., nuclei acids, proteins or other biological structures) that binds to the target analyte (Weetall, 1996). Bioreceptors are immobilized on a transducer surface to ensure specific detection. Novel bioreceptors are currently under development to replace traditional antibody-based methods. Notably, aptamers—such as peptide aptamers and oligonucleotide aptamers, comprised of single-stranded DNA or RNA—are emerging as promising alternatives (Parkhey and Mohan, 2018).

Second, the biological binding event must be converted into a physicochemical signal. Transducers translate this biological signal into a quantifiable one, which can be mechanical (force, pressure, displacement, acceleration), optical (light intensity, refractive index) or electrical (current, potential). Once transduced the signal can be processed (filtering, amplification) and transformed into pertinent chemical data (the third stage in the process).

### 1.3 Target analytes

An analyte is a substance whose chemical constituents are to be identified or measured (Soanes and Stevenson, 2008). Understanding current and future sensor applications for crime reduction requires identifying typical target analytes. This systematic review will categorize common analytes, including illicit drugs, fingerprints, and body fluids. For instance, sensors that detect blood stains at crime scenes can provide evidence such as blood type, DNA, and links to suspects or victims. Fast, low-cost, and reliable sensors are essential, and with advancements in technology comes the ability to detect a greater number of target analytes in smaller quantities (Gove and Durini, 2014).

### 1.4 Sensing technologies

Once target analytes are identified, suitable sensing technologies must be determined. Lab-based technologies include mass spectrometry using analysis of mass-to-charge-ratios (Gross, 2017), polymerase chain reaction (PCR) for DNA amplification (Hue-Roye and Vege, 2008) and high-performance liquid chromatography (HPLC) for rapid component separation (Kazakevich and Lobrutto, 2007).

Portable, lower cost technologies include electrochemical, colorimetric, immunoassay, luminescence and surface-enhance Raman spectroscopy (SERS). Electrochemical sensors use a recognition element coupled to an electrochemical transducer to give information about chemical composition (Yáñez-Sedeño et al., 2019). Voltammetry, a common electrochemical method, provides advantages over other electrochemical detection methods in portability (Ribeiro et al., 2020), matrix tolerance (Haghighi et al., 2020) and tolerance against potential interferents (Grothe et al., 2021). Colorimetric sensors utilise a specific indicator or reagent that reacts selectively with the target analyte providing a colour change for identification (Suslick et al., 2004). Immunoassays use antibodies or antigens to measure analyte presence or concentration (Diamandis and Christopoulos, 1996). Luminescence, including fluorescence, phosphorescence and chemiluminescence, uses characteristics such as intensity, wavelength and duration of emitted light to provide information about the target analyte (Holliday, 2016). Raman spectroscopy uses the interaction of molecular vibrations with light to provide non-destructive information about crystallinity, chemical structure and molecular interactions (Long, 1977). SERS enhances Raman scattering of molecules when they are adsorbed on or near to a SERS-active surface including nanostructures made from gold or silver (Xiu et al., 2021).

#### 1.4.1 Nanomaterials

Nanomaterials are essential for advancing diagnostic technologies, offering tailored properties in biomaterials (Bollella and Katz, 2020; Kim, 2017). Defined as substances with at least one dimension in the nanometer range (10^−9^ m), their small size confers unique properties attracting significant research interest due to their low cost (limited materials usage) and uniquely size-dependent properties (Amiri et al., 2021). Recent studies emphasize the role of nanomaterial-based biosensors, especially in electrochemical technologies, in enhancing portable devices by improving biocompatibility, stability, surface energy, and signal amplification (Su et al., 2017). Nano-coatings have also advanced bioreceptor immobilization, preventing non-specific binding (Bhalla et al., 2016). These developments address demands for biosensors with enhanced selectivity, sensitivity, rapid response and low cost (Su et al., 2017). Therefore, future research on crime reducing sensors will likely focus on nanomaterial-based biosensors.

### 1.5 Low-cost, on-site and performance

To date, devices have been costly and used complex methods which require an expert to undertake analyses. However, given budget constraints in policing and the ubiquity of some problems (e.g., drug driving), the next-generation of sensing devices will increasingly need to prioritise optimal performance during usage and post-storage, as well as being user-friendly and affordable (both in operation and production) (Weetall, 1996).

### 1.6 Systematic review

To understand advances in the use of sensing technologies to reduce crime and to map out a future research agenda, a systematic review (SR) was conducted. Ad hoc literature reviews present often sparse and biased coverage of an existing literature, whereas SRs are constructed to reduce bias by using transparent and systematic search approaches, enabling the extraction of as great a proportion of the existing evidence as is feasible on a subject (Cockbain et al., 2018). SRs are typically undertaken to gather evidence on “what works” in specific medicine and healthcare scenarios (Curtis and Cairncross, 2003) where data is plentiful. But SRs can also be employed for broader reviews for emerging issues (Blythe and Johnson, 2019; Elgabry et al., 2020), as is the case here.

### 1.7 Existing literature

Existing literature reviews on target analytes and sensing technologies for combating crime often focus on specific areas without a systematic approach. For instance, *Honeychurch’s* (Honeychurch, 2019) review on electroanalytical-based techniques for detecting benzodiazepines is detailed but narrow in scope, focussing on a specific analyte and sensing technology.

A 2020 special issue of *Biosensors* (Bollella and Katz, 2020), “*The Potential of (bio)sensors for the Forensic Sciences*” highlighted the potential of biosensors in forensic science. The special issue examines the crucial role biosensors can play in efficiently and accurately improving the techniques of crime detection (Bollella and Katz, 2020). However, although this review is broader in its approach than other articles, dealing with many target analytes, the sensing technology considered was limited to electrochemical biosensors.

Therefore, a wider scoped review of analytes and sensing technologies to reduce crime is of clear value to capitalise on recent advances in other sectors (Parkhey and Mohan, 2018). As far as we are aware, this is the first systematic review to scope all potential target analytes and sensing technologies of a sensor for combating crime. The overarching aims of the review are to understand:

1. What analytes are targeted in sensors for combating crime?
2. What sensing technologies have been used to combat crime?
3. What are the most recent directions of on-site and low-cost sensing devices?
4. What are the possible future avenues for further research?

## 2 Methodology and design

A SR protocol, developed using the Preferred Reporting Items for Systematic Review and Meta-analysis Protocols (PRISMA-P) guidelines (Shamseer et al., 2015), defined the scope of the review and the search strategy (see below). The protocol and search query were reviewed by an academic librarian with expertise in systematic reviews and updated in response to feedback.

### 2.1 Information sources

Gusenbauer and Haddaway (2020) identified principle academic search systems used in SRs. Of those identified, the most suitable were: ProQuest, PubMed and Web of Science. Searches focused on the title, abstract and keywords of articles, along with the field tag used (noft, Ti/Ab and TS respectively).

In addition, to find articles that may be missed by this search strategy, a chain citation technique (backward search) and the snowballing (forward search) of key studies identified was used (Cribbin, 2011).

### 2.2 Search query

To locate articles, a search query was refined, comprising two components: a sensing device concept and a criminal application concept. Before conducting the search, search terms were piloted and refined to achieve a balance between sensitivity (retrieving a high proportion of relevant articles), and precision (retrieving a low proportion of irrelevant articles). Relevant terms and synonyms were used to facilitate a systematic search.

Concept 1:

biosens* OR biomaterial* OR nanomaterial* OR nanoparticle* OR nanotechnolog* OR biotechnology* OR sensor*

Concept 2:

crime* OR criminal* OR offend* OR forensic* OR terror* OR illegal* OR illicit* OR unlawful*

Where the truncation character (*) retrieves variations of the search term, for example, crime* returns articles that include the term crime and crimes.

### 2.3 Eligibility criteria

The exclusion and inclusion criteria used to sift articles followed the PICOS format (Methley et al., 2014) and were applied at both the title and abstract, and full-text screening stages. The PICOS criteria used were:

Population (P): In contrast to medical studies, the population was less critical. Articles from the last 8 years (13/05/2016–13/05/2023) were considered, as older publications were less likely to be relevant due to rapid advances in sensing technology.

Interventions (I): Included articles focused on current or potential future uses of chemical and biochemical sensing devices to combat crime. Studies on other (sensing) technologies, such as new or emerging computer technologies (e.g., wireless sensor networks, IoT, machine learning), image and sound processing and those examining cyber or data security were excluded.

Outcomes (O): Measured whether the sensing technology could detect a specific target analyte.

Study Types (S): Included peer-reviewed journal articles, government or official documents (legal documents), and academic theses. Excluded were commentaries, books/book reviews, opinions, and working papers. Studies had to be written in English language and journal articles peer-reviewed, with the latter ensuring publications were of sufficient quality (Koshy et al., 2018).

### 2.4 Study selection

**FIGURE 1:**
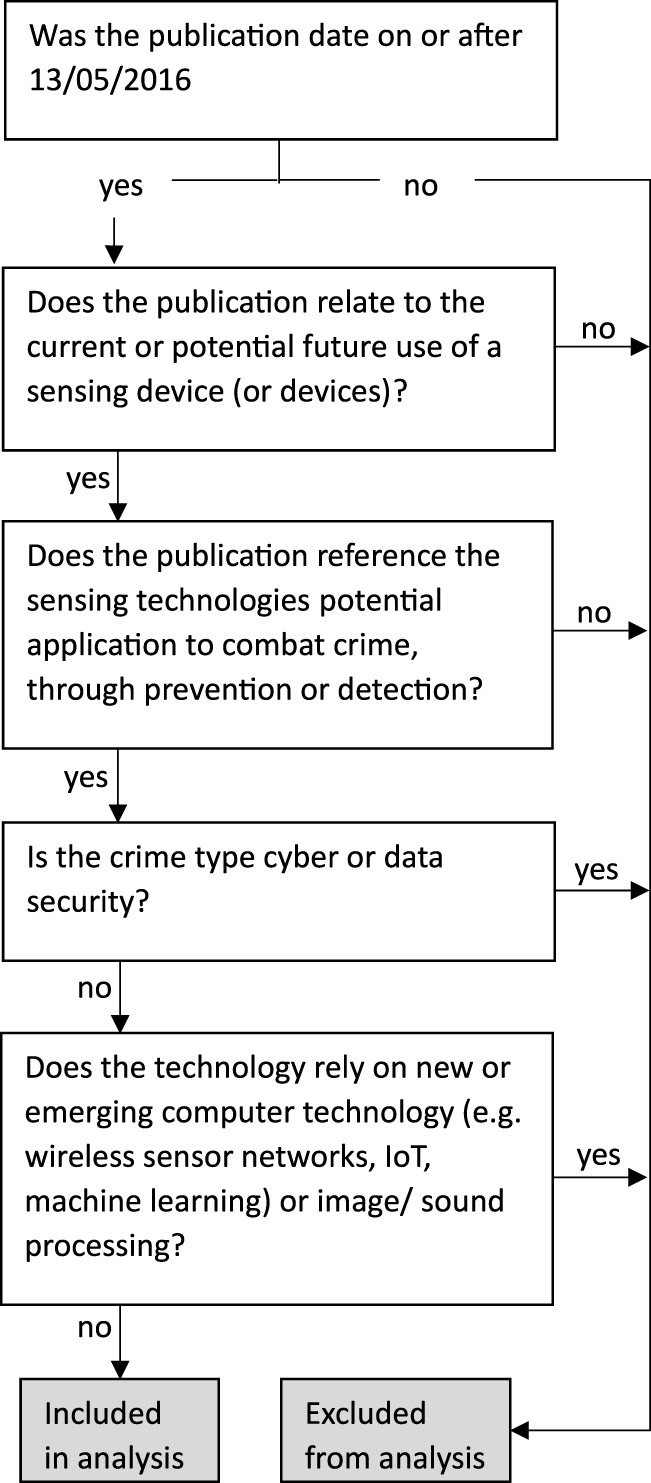
Schematic of the inclusion decision tree.

In stage 1, titles and abstracts were screened using the PICOS criteria and the EPPI-Reviewer 4.0 software (Thomas et al., 2010). Figure 1 guided decision making to ensure consistency by the primary reviewer and to assist co-reviewers during an inter-rater reliability (IRR) exercise. To assess IRR, a random sample of 5% of the identified publications were assessed on title and abstract by two other reviewers. Agreement between first reviewer and co-reviewers was measured using the prevalence-adjusted and bias-adjusted kappa (PABAK) statistic (Elgabry et al., 2020; Smith et al., 2011) (Equation 1), yielding values of 0.81 and 0.84, indicating very good agreement. The primary reviewer subsequently screened the full text of all articles included during stage 1 of the screening process.

$$\left[\frac{\left(no.\;papers\;with\;reviewer\;agreement\right)}{\left(total\;no.\;papers\right)}-0.5\right]/0.5$$ (1)

### 2.5 Data extraction and analysis

For each article, we extracted pertinent information, including study identifiers (publication year, author(s), publication type) and outcomes (target analyte, sensing technology, detection method). Following data extraction, findings were synthesised using a thematic analysis (Clarke and Braun, 2017).

### 2.6 Further exclusion

Following the initial systematic review and summary of core information, a more in-depth study was then performed on a sub-set of included publications. As noted in a special issue of *Biosensors* (Bollella and Katz, 2020) the future of evidence analysis in criminal investigations relies on the development of rapid, decentralised and low-cost testing by untrained individuals. For these reasons and because of the fast pace of research in sensing technologies, a more detailed analysis was carried out. The further eligibility criteria for the second stage were that.

• The publications date was on or after 01/01/2020
• The sensing technology was low-cost (as detailed in Section 1.4)

## 3 Results and discussion

In the results section, we first present findings from the initial SR screening, examining trends in publication numbers and target analytes identified over the last 8 years. Secondly, our further screening results are presented and discussed, focussing on publications from the last four and a half years that discuss low-cost sensing technologies. Publications are categorised by target analyte addressed with discussions looking at numbers of publications, sensing technologies used, and key themes identified. Current technologies used by law enforcement and local authorities are evaluated, and areas where further research could significantly impact crime reduction, based on SR results, highlighted.

### 3.1 Initial screening (2016-2023)

**FIGURE 2:**
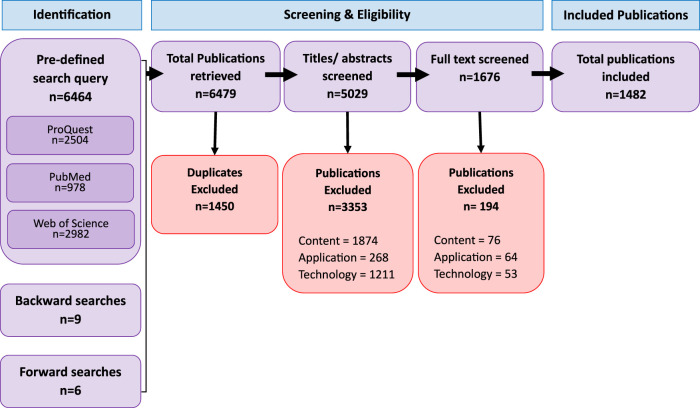
PRISMA flowchart summarising the inclusion and exclusion steps of the systematic review performed.

Figure 2 shows the number of articles identified, included and excluded at each review stage. The pre-defined search query resulted in 2,504 results from ProQuest, 978 from PubMed and 2,982 from Web of Science, plus 9 from backward searches and 6 from forward searches. Of the 6,479 publications initially identified, 22% were duplicates and removed. Title and abstract screening excluded 67% of the remaining publications, and full-text screening excluded another 12%. Ultimately, 1,482 publications were carried forward for analysis.

There was a year-on-year increase in included publications, from 134 in 2017 to more than double that amount (Ge et al., 2020) in 2022. This growth reflects increased research and development of biosensors (Parkhey and Mohan, 2018), underscoring the need to explore their potential applications for crime reduction.

#### 3.1.1 Eleven target analytes identified

Categorising included publications by target analytes is crucial for understanding current trends in sensing devices and enabling a more systematic analysis. Thematic analysis of the included publications identified eleven identified target analytes:

1. Fire
2. Gunshot
3. Counterfeits and documentation
4. Pollutants (e.g., adulterated fuels, industrial waste)
5. Body fluids (any target analyte that could be found in body fluids e.g., seminal or vaginal fluid, salivary amylase, blood, urine, DNA)
6. Explosives
7. Poisons and toxins (e.g., mycotoxins, pesticides)
8. Fingerprints
9. Food safety (e.g., food poisoning, adulteration)
10. Illicit drugs–As illicit drugs represented a large proportion of the included publications the category was further subdivided [depressants, stimulants, hallucinogens, pharmaceuticals, dissociates, cannabinoids and opioids per Target Zero (Author Anonymous, 2025a) Zero^1^]
11. Other (e.g., radioactive materials, illegal wildlife trade)

**FIGURE 3:**
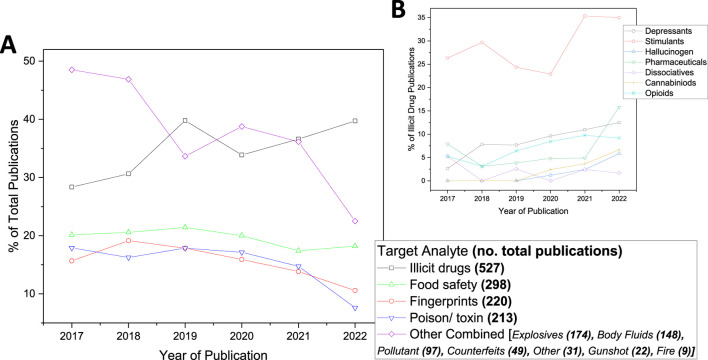
**(A)** Trends in the percentage of publications relating to each identified target analyte from 2016 to 2023*. Noting that some publications related to multiple target analytes and therefore the total percentage is seen to sum to more than 100%. **(B)** Trends in percentage of different categories of illicit drug publications identified from 2017 to 2022. Noting that some publications related to multiple target drugs or were not assigned to one of the 7 drug categories and therefore the total percentage is seen not to sum 100%. [*where 2016 and 2023 are not shown as data collection was only for half of these years.].

Figure 3A shows that the largest volume of included publications (36%) focussed on illicit drugs, followed by food safety (20%), fingerprints (15%) and poisons/toxins (14%). The large proportion of publications in these areas indicates strong research interest and rapid development but also reflects the broad scope of some of these categories. For example, the illicit drugs category is expansive. Additionally, Figure 3A highlights less interest and development in sensors for fire and gunshot analysis.

Over the past 8 years, the proportion of publications for each target analyte has remained fairly constant (Figure 3A). However, publications on fingerprint and poison/toxin detection have decreased, while those on illicit drugs have increased, especially from 2020 to 2022, echoing a growing global concern about substance abuse (United Nations, 2023) and could thus represent an important avenue for further research.

Figure 3B shows that overall about 30% of illicit drug publications focussed on the design of sensors to detect stimulant abuse. Opioid-related publications are seen to have increased rapidly from 2019 onwards. This aligns with the rising opioid crisis and persistent stimulant misuse. According to the US National Survey on Drug Use and Health (NSDUH), reported drug use in the US increased by 47% from 2016 to 2022 (Author Anonymous, 2025b), with 13% of Americans starting or increasing substance use to cope with COVID-19 stress (Abramson, 2021). These trends suggest the time critical nature of sensor development in illicit drug detection.

#### 3.1.2 Sensing technologies used

Eight different types of sensing elements were identified within the included publications and were classified as high or low-cost technologies (detailed in Section 1.4). High-cost technologies, requiring expensive equipment and trained professionals, included mass spectrometry (5% of publications), PCR (1%), and HPLC (2%). Low-cost technologies, suitable for use by untrained persons, included electrochemical (27%), colorimetric (11%), immunoassay (18%), luminescence (32%), and SERS (15%).

### 3.2 Detailed analyses (2020-2023)

After the initial overview, a more in-depth analysis was conducted on articles published from 2020 onwards, focusing on low-cost sensing technologies due to the reasons highlighted in the methods section and the fast-pace of sensor development. A total of 590 publications were excluded as they had a publication date before 01/01/2020 and 110 were excluded due to the high-cost of the technology discussed. This left 782 publications for more detailed analysis.

High- and low-cost technologies are classified based on material costs, instrumentation complexity, expenses, and accessibility. Low-cost methods use inexpensive materials and portable equipment for on-site testing, while high-cost methods require complex preparation, advanced instruments, and specialised operators for centralised labs.

**FIGURE 4:**
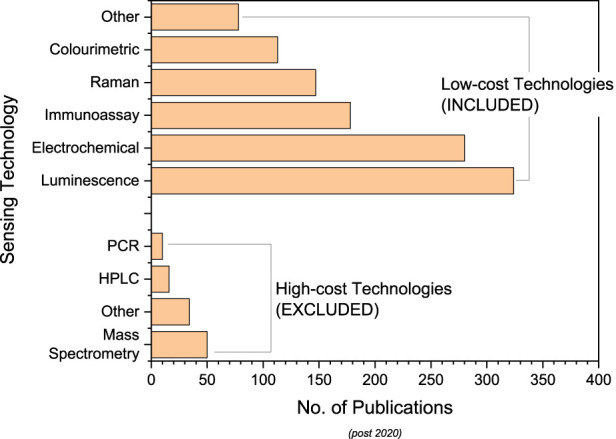
Number of publications identified relating to different low-cost sensing technologies (included) and high-cost sensing technologies (excluded).

Within the high-cost publications, mass spectrometry accounted for 45% followed by HPLC for 15% (Figure 4). Although these technologies allow accurate analysis [see Chiang et al. (2019)] their expense, requirement of trained users and time intensive nature makes them unsuitable for mass-market production and irrelevant to the rest of this review.

Low-cost publications comprised 88% of those identified from 2020 onwards, with luminescence and electrochemical technologies being the most prevalent (Figure 4). The literature focus on these technologies indicates that they are at the forefront of work being done in low-cost sensing development.

**FIGURE 5:**
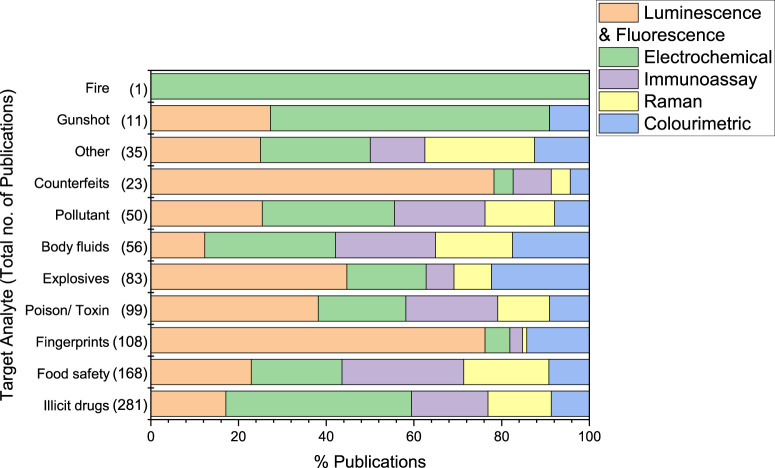
Plot showing the number of publications included in the further screening review. The percentage of publications assigned to a sensing technology for each target analyte has been indicated.

The subsequent sections discuss the above outlined publications, organised by target analyte (Figure 5). Tables summarise key themes identified in the included publications, categorised by sensing technology. Key themes and publications are explored further within the accompanying text to outline current technologies and potential areas for further research. Alongside the publications reviewed in the SR, an outline of the current sensing technologies used by UK police and authorities is provided. The shortfalls of these technologies are discussed, and the findings from the SR used to suggest the most promising directions for further research to address these issues.

#### 3.2.1 Fire

Sensors in fire analysis detect, map and trace fire sources. Only one publication focused on fire, highlighting minimal current interest. The identified publication reviewed metal oxide (MOx) electrochemical sensors (Shalini Devi et al., 2021) for hazard-surveillance and risk investigation, including fire-hazards, chemical-warfare agents, oil-spills and explosives. MOx sensors, combined with airflow detectors and specific algorithms, have been used in mobile robots for fire analysis. These developments are made possible by novel nanoarchitectural patterns which enhance sensitivity and the possibility of multi-analyte sensing using array sensors and blended composites. The authors noted that improving thermal damage prevention, response, recovery times, and robot mobility is necessary to realize the potential of these devices.

In the UK, police and fire services collaborate on-site to determine fire causes, origins and behaviours, collecting evidence such as fire debris and electrical appliances for analysis (Cellmark, 2025a; Northamptonshire Fire and Rescue Service Fire Investigation FI, 2013). Central laboratories use gas chromatography-mass spectrometry (GC-MS) to identify accelerants, origin and fire behaviour (Abel et al., 2018). Fire investigation dogs are used to pinpoint traces of potential accelerants (Forensic Technology Center of Excellence, 2021) and thermal imaging cameras to identify hotspots and origins (Police Law Enforcement Solutions, 2018).

Current issues include the portability of GC-MS and the lack of specificity of canine detection. On-site detection is necessary to reduce delays caused by evidence backlogs (Forensic Technology Center of Excellence, 2021). Whilst portable GC-MS shows promise, further research is needed to confirm field accuracy (Forensic Technology Center of Excellence, 2021). Research into portable MOx sensors (Shalini Devi et al., 2021) offers a rapid alternative to central laboratory GC-MS testing and increased specificity over canine units.

#### 3.2.2 Gunshot

For gunshot analysis, sensors help identify gunshot residue (GSR) on suspects or at a crime scene. Only 11 publications focussed on gunshot analysis, with electrochemical sensing being the most common (64% of papers) (Table 1). Shrivastava et al. (2021a) and Shrivastava et al. (2021b) described a handheld colorimetric sensor for rapid detection of lead and barium in GSR using polyvinyl alcohol capped silver nanoparticles and malonate capped gold nanoparticles respectively, ensuring rapid detection with no interference from other metal ions.

**TABLE 1:** Summary of included publications relating to the use of sensors in gunshot analysis, alongside details of technologies currently used by UK police and authorities for on-site gunshot analysis.

Sensing technology	Description	Research papers	Review papers
*Colourimetric*	Shrivastava et al. detailed the use of a handheld sensor using PVA capped AgNPs for detection of lead in gunshot residue and malonate capped AuNPs for detection of barium without interference from other metal ions	Shrivastava et al. (2021a), Shrivastava et al. (2021b)	
*Electrochemical*	Key themes were the use of 3D printing, doping of electrodes and *ad hoc* literature reviews	Castro et al. (2020), Promsuwan et al. (2020a), Chedid et al. (2023), McKeever et al. (2022)	Harshey et al. (2021), Shrivastava et al. (2021c), Castro et al. (2022)
*Luminescence*	Papers detail the detection of novel nontoxic ammunition alongside novel techniques to detect common metal ions within gunshot residue	Chedid et al. (2023), Chaiendoo et al. (2021)	Shrivastava et al. (2021c)
*Other*	Ad hoc review of LIBS		Senesi et al. (2021)
Currently used by UK police and authorities			
UK police currently use colourimetric spot tests Modified Griess Test and Sodium Rhodizonate Test to detect nitrite and lead residues respectively (Krishna and Ahuja, 2023)			

Electrochemical sensing studies have common themes of 3D printing and doped electrodes (the addition of impurities to modulate properties of the electrodes (Castro et al., 2020) alongside many review articles (36% of publications). Castro et al. (2020) detail the use of 3D-printed electrodes for simultaneous and semi-quantitative detection of lead and antimony (both present in GSR) without the need for sample preparation. Simultaneous detection reduces the numbers of tests needed to ensure identification of different types of GSR, reducing costs and time. Furthermore, the augmentation of electrode response through doping is shown to enable detection at lower concentrations. Promsuwan et al. (2020a) demonstrated enhanced electrocatalytic response with palladium doped glassy carbon microspheres, while McKeever et al. (2022) used voltametric electrodes with magnetic nanoparticles for propellant stabilizer detection. Several *ad hoc* reviews highlighted the benefits of advanced electrochemical methods such as single strip-based techniques over current heavy instrumentation, such as mass spectrometry (Shrivastava et al., 2021c; Harshey et al., 2021) due to their user-friendliness, sensitivity, and cost-effectiveness A third more general review highlighted the benefits of 3D-printing in electrochemical sensing as a powerful, affordable and accessible tool (Castro et al., 2022) emphasising the importance of these research endeavours reaching end users.

Chedid et al. (2023) discussed luminescence sensors for new nontoxic ammunition, which requires alternative detection techniques due to a lack of the conventional metals for detection of GSR. The presence of an inorganic luminescent chemical marker in GSR is shown to be effectively detected using square-wave voltammetry on a carbon paste electrode. Sensors able to detect these new residues will be essential moving forward and demonstrates the need for constant re-evaluation of the current target analytes and therefore techniques needed to detect them.

Senesi et al. (2021) provide an *ad hoc* review of laser-induced breakdown spectroscopy (LIBS) for gunpowder origin analysis looking at both prototype instruments and commercially available analysers. Key areas for future research focus on improving the portability and analysis speed of LIBS instruments outside the laboratory while maintaining high performance.

In the UK, police use colorimetric spot tests (Modified Griess Test and Sodium Rhodizonate Test) for on-site GSR analysis (Krishna and Ahuja, 2023), but these have low specificity and can degrade samples (Shrivastava et al., 2021c). Samples are often sent to labs for analysis using scanning electron microscopes (SEM), LIBS, and SERS (Cellmark, 2025b). A move towards field-deployable SEM can provide improvements to current investigative methods (Cellmark, 2025b).

To address these drawbacks, research detailed in the SR highlights promising advancements. Colorimetric sensors using capped nanoparticles (NPs) have been shown to reduce interference from other metal ions in samples (Shrivastava et al., 2021a; Shrivastava et al., 2021b), overcoming issues faced by current devices. Additionally, electrochemical methods under development demonstrate increased specificity compared to current spot tests, with the potential for simultaneous detection. These methods also offer benefits such as cheaper instrumentation and simpler user interfaces, thereby overcoming cost and training barriers (Castro et al., 2020; Castro et al., 2022).

##### 3.2.3 Counterfeits/documentation

Sensors for counterfeit and documentation analysis help identify authentic and forged documents and develop anticounterfeiting labels. 23 (3%) publications were concerned with counterfeit and document analysis, with luminescence sensing technology being most commonly used (78% of papers, see Table 2).

**TABLE 2:** Summary of included publications relating to the use of sensors in counterfeits or documentation analysis, alongside details of technologies currently used by UK police and authorities for on-site counterfeit and documentation analysis.

Sensing technology	Description	Research papers	Review papers
*Colourimetric*	Security inks using blue emitters have been demonstrated in anticounterfeiting labels	Kumar and Singh (2023)	
*Electrochemical*	An *ad hoc* review looking at the potential for magnetic nanoparticles to enhance electrochemical detection for forensic applications is presented		Nadar et al. (2021)
*Immunoassay*	Two *ad hoc* reviews focus on the potential for aggregation-induced emission and magnetic nanoparticles to apply advances made in other sectors with this technology to forensic detection		Nadar et al. (2021), Yan et al. (2021)
*Luminescence*	Key themes were the use of doped materials and ecofriendly materials. Alongside *ad hoc* literature reviews	Fouad and Saif (2020), Guleria et al. (2020), Kamal and Saif (2020), Naik et al. (2020), Praveen et al. (2020), Srivastava et al. (2020), Suresh et al. (2020), Szczeszak et al. (2020), Abdollahi et al. (2022), Ansari et al. (2022), Han et al. (2022), Li et al. (2022a), Narasimhamurthy et al. (2021), Ravindra et al. (2021), Dwivedi et al. (2023)	Nadar et al. (2021), Yan et al. (2021), Tomar et al. (2023), Verhagen and Kelarakis (2020)
*Raman*	Broad overview in banknote security materials and analytical techniques that are used in detecting counterfeits		Tomar et al. (2023)
*Other*	Ad hoc review of LIBS and two papers detailing IR using chemometrics for printer ink analysis and banknote authentication	Paxton et al. (2021), Nurfarhana et al. (2022)	Senesi et al. (2021)
Currently used by UK police and authorities			
UK police use various light sources, including ultraviolet (UV) and infrared (IR) light, for identifying security features (College of Policing, 2017). They also employ magnetic ink detectors and portable spectrometers for this purpose (Regula Forensics, 2025)			

Publications identified relating to the use of luminescence sensing have common themes of doped materials and ecofriendly materials along with *ad hoc* reviews of the literature. Five *ad hoc* reviews identified examined advances in materials and nanomaterials from other research areas being applied to counterfeit sensing technologies. For example, the use of magnetic nanoclusters with super-magnetic behaviour and smaller dimensions shows significant potential for anticounterfeiting with rapid and full reversible optical responses after magnetic field application (Nadar et al., 2021). However, the shelf-life of these nanoclusters remains uncertain, necessitating further research. Research exploring the aggregation-induced emission (AIE) phenomenon, known for its remarkable luminescence properties, has proven successful in applications such as anticounterfeiting banknotes and confidential documents (Yan et al., 2021). Tetraphenylethene derivatives, exhibiting fluorescence under ultraviolet irradiation but reverting to white colour within 1 min of excitation, demonstrate this effectiveness. Carbon dots (CDs) have also been discussed with a review highlighting their superior fluorescence, low-cost, non-toxic and colour-tuneable nature (Verhagen and Kelarakis, 2020). For example, CDs can be incorporated into inks capable of functioning as novel barcodes and nanotags for authentication and anticounterfeit applications.

The doping of materials has improved detection capabilities. Kamal and Saif (2020) detail the use of barium tungstate doped with terbium ion green nanophosphor and Naik et al. (2020) discuss the use of nitrogen-doped carbon dot threads as fluorescent ink in potential anti-counterfeiting applications.

The need for environmentally friendly and non-toxic materials were key research themes (Szczeszak et al., 2020). Lanthanide-doped SrF_2_ nanoparticles combined with luminescent cellulose fibres have been developed for anti-counterfeiting applications, where they are invisible under ambient light but bright green under near-infrared light (Szczeszak et al., 2020). This use of organic fibres is both beneficial to the environment and reduces associated costs with material manufacture. Abdollahi et al. (2022) detail the use of metal-free and eco-friendly photoluminescent polymer nanoparticles based on oxazolidine as a sustainable alternative for anticounterfeiting.

Tomar et al. (2023) present a broad overview of banknote security materials and analytical techniques for detecting counterfeits. They discuss new anti-counterfeiting materials and fluorescent nanoparticles that can be used as anti-counterfeiting inks with technologies such as Raman spectroscopy.

Publications not using luminescence technologies included the use colourimetric techniques to produce security ink for anticounterfeiting labels making it easier to detect fakes and trace their origin or dispersal (Kumar and Singh, 2023). 1,8-naphthalimide-based blue emitters non-covalently doped on silica have been demonstrated, with excellent results, in real-world situations. Infrared (IR) technology has also been demonstrated alongside novel chemometric methods to provide successful forensic analysis on printer inks (Paxton et al., 2021) and authentication for banknotes (Nurfarhana et al., 2022).

Counterfeit detection involves examining security features such as watermarks, holograms, and special inks. The UK police currently use various tools, including ultraviolet (UV) and infrared (IR) light, to check for security features that are invisible under normal lighting (College of Policing, 2017). However, these methods can produce false positives or negatives due to environmental factors or wear and tear and are limited to features specifically designed to be UV or IR reactive. Magnetic ink detectors are also employed to identify the presence of magnetic inks found in genuine banknotes and important documents, which are typically absent in most counterfeits (Regula Forensics, 2025). These detectors can be affected by nearby electronic devices or metal objects, and sophisticated counterfeiters increasingly use magnetic inks that can deceive them. Portable spectrometers, such as the Regula 4,115 (Regula Forensics, 2025), are used for the express verification of banknotes, featuring a built-in camera and various light sources for a comprehensive examination of security features. However, these devices are costly and require significant training for proper use.

To address these challenges, recent research highlighted in the SR points to promising advancements in colourimetric and luminescent sensing. These advances involve techniques such as doping (Kamal and Saif, 2020; Naik et al., 2020) and the use of environmentally friendly materials (Szczeszak et al., 2020; Abdollahi et al., 2022) to enhance detection capabilities and reduce costs, respectively. Additionally, magnetic nanoclusters exhibit super-magnetic behaviour compared to conventional magnetic inks (Nadar et al., 2021). Their distinct and durable magnetic properties make them difficult to replicate. However, further research is needed to commercialize these technologies.

#### 3.2.4 Pollutants

With increasing environmental concerns, monitoring illegal discharges and pollutants is crucial. Among the 50 (6%) publications on pollutant analysis, electrochemical and luminescence technologies dominated, accounting for 70% (Table 3). Key themes identified included eco-friendly materials, dual detection (combining two sensing technologies for enhanced detection), law-enforcement approval and portability.

**TABLE 3:** Summary of included publications relating to the use of sensors in pollutant analysis, alongside details of technologies currently used by UK police and authorities for on-site pollutant analysis.

Sensing technology	Description	Research papers	Review papers
*Colourimetric*	Optical sensors for detection of illicit discharge into sewage and of illicit dyes. A focus on pollutants of emerging concern and the use of environmentally friendly materials in detection	Rocher et al. (2021), de Souza et al. (2022)	Pena-Pereira et al. (2021), Kamel and Khattab (2020), Neal et al. (2021)
*Electrochemical*	Key themes were the use of ecofriendly materials, law-enforcement approval and dual detection	Noviana et al. (2019), Yu et al. (2022a), Shalini Devi et al. (2021), Hakeem et al. (2022), Kabel et al. (2021), Karuppaiah et al. (2023), Shi et al. (2021), Singh et al. (2021), Kolacz et al. (2023), Singh et al. (2020), Myadam et al. (2021), Molinara et al. (2022)	Pena-Pereira et al. (2021), Kamel and Khattab (2020), Karimi-Maleh et al. (2020), Neal et al. (2021), Kushwaha et al. (2022), Castro et al. (2022), Kadu et al. (2023)
*Immunoassay*	A focus on dual detection using immunochromatography is seen	Wu et al. (2021a), Senra and Fonseca (2021), Yu et al. (2022a), Li et al. (2023a), Lan et al. (2020)	Pena-Pereira et al. (2021), Kamel and Khattab (2020), Karimi-Maleh et al. (2020), Nardo et al. (2021), Mao et al. (2021a), Uttpal et al. (2022), Bräuer et al. (2021), Dube et al. (2023)
*Luminescence*	Key themes were the use of ecofriendly materials, law enforcement approval and portability through miniaturisation	Wu et al. (2021a), Chengyi et al. (2020), Mao et al. (2021b), Rocher et al. (2021), Su et al. (2021), Liu et al. (2022a), Liu et al. (2023a), Warning et al. (2021), Lian et al. (2020a), Harsha et al. (2020), Ke et al. (2020)	Pena-Pereira et al. (2021), Kamel and Khattab (2020), Neal et al. (2021), Kushwaha et al. (2022), Uttpal et al. (2022)
*Raman*	A focus again here is seen on dual detection through the combination of SERS with other technologies such as TLC.	Vunckx et al. (2020), Yang et al. (2021a), Li et al. (2023a), Chang et al. (2022a), Jiang et al. (2021a), Nie et al. (2023), Vendamani et al. (2023), Xie et al. (2022a), Yao et al. (2022a)	Zhang et al. (2022a)
*Other*	IR shows potential to detect pollution in sewage. The use of cockroaches to assess the toxicity of environmental pollutant has been demonstrated	Rocher et al. (2022), Adedara et al. (2022)	
Currently used by UK police and authorities			
UK government organisations utilise instruments such as portable Raman spectrometers, photoionization detectors (PIDs) and x-ray fluorescence analysers (XRF) (Department for Environment, Food & Rural Affairs, 2025; Guidance, 2014)			

To reduce environmental and other costs, the use of eco-friendly materials is important, particularly for large-scale production. Senra and Fonseca (2021) demonstrated the potential for replacing expensive tyrosinases (type-3 copper metalloenzymes) with cost-effective freshwater ciliates, rapid-growing unicellular microeukaryotes. They employed virtual screening to compute binding energies between 3D models of these homologs, paving the way for more economical alternatives. Additionally, paper-based (Noviana et al., 2019) and cellulose-based (Kamel and Khattab, 2020) biosensors were highlighted as environmentally friendly alternatives to traditional substrates.

Dual detection sensors simultaneously measure multiple parameters or analytes by integrating different sensing technologies or methods, enhancing accuracy, sensitivity, and versatility across various applications (Lan et al., 2020). Zhang et al. (2022a) *ad hoc* review discussed using thin layer chromatography (TLC) coupled with SERS for on-site multi-component detection. The TLC chromatographic plate is used for high-throughput separation with SERS enabling quantitative detection of mixtures. However, further work is needed to mature the technology for on-site applications, including the use of porous materials or polymers to enhance separation efficiency and the application of machine learning to improve the accuracy of quantitative signal information. Immunochromatographic assay strip readers combining immunoassay and chromatography techniques were also noted in two papers demonstrating their ability to extend the range of detectable analytes (Wu et al., 2021a) and increase the speed of detection (Lan et al., 2020).

Pena-Pereira et al. (2021) present an extensive *ad hoc* review of miniaturised analytical methods for detecting emerging environmental contaminants (e.g., illicit drugs, surfactants and personal care products). They highlight opportunities for low-cost, field deployable devices with the possibility for creating big data sets at low cost, and the development of screening methods to be used before more expensive traditional sensing methods (e.g., gas chromatography-mass spectrometry) are used to validate results. However, challenges include law enforcement approval, stability of sensing elements and few commercially available set-ups. Approval from law-enforcement agencies is key in moving developed sensors from small to large-scale use.

For identifying and measuring pollutants at crime scenes, accidents, or environmental incidents UK government organisations currently use instruments like GC-MS, Raman spectrometers, photoionization detectors (PIDs) and x-ray fluorescence analysers (XRF) (Department for Environment, Food & Rural Affairs, 2025; Guidance, 2014). Portable GCs require specialised training, regular maintenance and high initial and operational costs (Department for Environment, Food & Rural Affairs, 2025). PIDs are effective for detection of volatile organic compounds but not other pollutants (Zimmerman et al., 2020). XRF typically analyses only the surface layer of a sample, with interference from other elements and potential radiation exposure posing additional concerns (Department for Environment, Food & Rural Affairs, 2025).

Recent research highlighted in the SR indicates promising advances to tackle these current challenges. A crucial focus lies in addressing cost concerns by exploring more economical alternatives, both in material selection (Senra and Fonseca, 2021; Noviana et al., 2019; Kamel and Khattab, 2020) and screening methods (Pena-Pereira et al., 2021), which have shown promise. Additionally, current shortcomings in the capacity of tests to detect multiple target analytes have been emphasised. Dual detection methods have emerged as a solution, enhancing the range and speed of analyte detection, though further research is needed to mature these technologies for practical use (Zhang et al., 2022a; Wu et al., 2021a).

#### 3.2.5 Body fluids

Body fluid analysis, encompassing a wide range of analytes from salivary amylase to DNA, is crucial for combating crime. 56 (7%) publications were concerned with body fluid analysis, with all sensing technologies being fairly evenly represented (Table 4). Key themes identified included the importance of ecofriendly materials and dual detection.

**TABLE 4:** Summary of included publications relating to the use of sensors in body fluid analysis, alongside details of technologies currently used by UK police and authorities for on-site body fluid analysis.

Sensing technology	Description	Research papers	Review papers
*Colourimetric*	Papers have focused on DNA analysis and alpha amylase detection for identification of saliva samples	Tortajada-Genaro et al. (2022), Khajouei et al. (2020), Li et al. (2020a), Calabretta et al. (2021)	Kamel and Khattab (2020), McGoldrick and Halámek (2020), Adhikary and Banerjee (2021), Nadar et al. (2021), Neal et al. (2021)
*Electrochemical*	A key theme was the use of eco-friendly materials	Noviana et al. (2019), Ataide et al. (2020), Sanli et al. (2020), Kim (2021), Zhang et al. (2020a), Cook and Honeychurch (2021), Izham et al. (2022), Patiti et al. (2022), Pierpaoli et al. (2022), Kang et al. (2020a), Liao et al. (2020), Mishra et al. (2020), Sadeghi et al. (2020)	Kamel and Khattab (2020), Nadar et al. (2021), Mani et al. (2021), Neal et al. (2021)
*Immunoassay*	Papers focus on detection of fluids from DNA using AuNP-protein nanopores to bioanalytes such as uranyl in urine using aptamer-modified nanosensor arrays	Garcia-Cruz et al. (2020), Meir et al. (2020), Loredana et al. (2020), Sanli et al. (2020), Tang et al. (2020), Tortajada-Genaro et al. (2022), Lee et al. (2022a), Mishra et al. (2020)	Kamel and Khattab (2020), McGoldrick and Halámek (2020), Nadar et al. (2021), Nardo et al. (2021), Bräuer et al. (2021)
*Luminescence*	Key themes were the use of eco-friendly materials and dual detection	Calabretta et al. (2021), Kang et al. (2020b), Kasry et al. (2021), Badshah et al. (2020), Bazzi et al. (2023), Fan et al. (2023), Kasry et al. (2021), Chen et al. (2021a), Melman et al. (2022), Lian et al. (2020a), Khunoana et al. (2020)	Kamel and Khattab (2020), Nadar et al. (2021), Neal et al. (2021)
*Raman*	Papers have focused on the use of Raman to differentiate between hair dyes and to identify blood samples	Vendamani et al. (2023), Atta and Vo-Dinh (2023), Bruner and Monjardez (2023), Cai et al. (2022a), Erturan et al. (2023), Gautam et al. (2022), Higgins and Kurouski (2023), Ondieki et al. (2023), Reese et al. (2021), Suarez et al. (2023), Liu et al. (2021)	
*Other*	Papers have mostly focused on DNA identification through microfluidic and nanopore sensing approaches. Analysis of blood and semen stains is also discussed	Bruijns et al. (2020), Dhar et al. (2021), Mereuta et al. (2022), Schmidt et al. (2022), Yildirim et al. (2023)	
Currently used by UK police and authorities			
UK police use luminol and Bluestar for blood detection using luminescence technologies (College of Policing, 2017; Forensics Library, 2024). Rapid Stain Identification (RSID) tests use immunochromatographic assay technology for saliva, semen and urine detection (Forensic Body Fluid Analysis Services, 2024; Harbison and Fleming, 2016). Portable DNA analysers are also employed such as the RapidHIT ID System (Thermo Fisher Scientific, 2024)			

Paper-based methods show promise for on-site analysis of mitochondrial DNA and salivary amylase (Dhar et al., 2021). One paper-based device using core-shell nanoparticles identifies saliva by showing a visible colour change when the shell is disrupted by alpha-amylase exposure (Adhikary and Banerjee, 2021). For mass on-site analysis further testing on human saliva samples is needed. Kamel and Khattab (2020) discuss recent advances in cellulose-based biosensors for medical diagnosis. The use of these alternatives present renewable, less toxic and cheaper solutions to existing sensing devices.

Another key area of research is in the coupling of sensing technologies for a dual detection sensor. One example is the development of a grating-coupler as a transducer to excite surface-plasmon combined with fluorescence to identify DNA sequences creating quick and sensitive on-site analysis potential (Kasry et al., 2021). Lateral flow immunoassays (LFIAs) offer rapid, cost-effective on-site applications, and have become widespread in recent years (Nardo et al., 2021). In their *ad hoc* literature review, Nardo et al. (2021) report that LFIAs have been developed to analyse prostate specific antigens (Kishbaugh et al., 2019), salivary amylase (Kishbaugh et al., 2019) and human haemoglobin (Murahashi et al., 2020).

UK police use various sensors to detect body fluids at crime scenes. Luminol and bluestar cause a luminescent reaction upon contact with haemoglobin, making bloodstains visible even if they e been cleaned or are not visible to the naked eye (College of Policing, 2017; Forensics Library, 2024). There are issues with false positives from certain metals and cleaning agents, DNA degradation complicating subsequent analysis, and the short-lived luminescent reaction making it difficult to document complex crime scenes effectively (College of Policing, 2017; Forensics Library, 2024). Rapid Stain Identification (RSID) tests detect specific body fluids like saliva, semen, and urine using specific markers like prostate-specific antigen for semen (Forensic Body Fluid Analysis Services, 2024; Harbison and Fleming, 2016). RSID tests, while specific, can still suffer from cross-reactivity and sensitivity issues with the presence of other body fluids (Harbison and Fleming, 2016). Portable DNA analysers, such as the RapidHIT ID System (Thermo Fisher Scientific, 2024), can be useful in time-sensitive investigations in helping identify individuals from biological samples (Forensics Library, 2024). Issues of cost and complexity in the operation of portable DNA analysers can be a limitation for smaller police departments alongside sensitivity to environmental conditions causing a reduction in performance and accuracy (Forensics Library, 2024).

Recent research highlighted in the SR indicates promising advancements to tackle current challenges. Research into eco-friendly materials has shown promise in paper-based DNA detection methods, which can reduce costs (Dhar et al., 2021). Additionally, the use of dual detection techniques has been explored, demonstrating potential for rapid and sensitive on-site DNA analysis (Kasry et al., 2021). Improvements to RSID tests are also being investigated, with methods such as core-shell nanoparticles being researched for rapid colourimetric testing (Adhikary and Banerjee, 2021). However, these methods require further testing on human samples before they can be commercially utilised.

#### 3.2.6 Explosives

Sensors in explosives analysis facilitate the (proactive) identification of potential explosive threats, such as during the screening of large crowds. 83 (10%) publications were concerned with explosives analysis, with luminescence sensing technology being the most commonly used (47% of papers, see Table 5). Key themes identified included dual detection, selective detection, 3D printing and portability of sensors.

**TABLE 5:** Summary of included publications relating to the use of sensors in explosives analysis, alongside details of technologies currently used by UK police and authorities for on-site explosives analysis.

Sensing technology	Description	Research papers	Review papers
*Colourimetric*	Key themes were the use of dual detection, selective detection and improvised explosives detection	Abuzalat et al. (2020), Elbasuney et al. (2020a), Raza et al. (2020), Batool et al. (2022a), Cao et al. (2022), Su et al. (2022), Zhang et al. (2022c), Huang et al. (2022a), Li et al. (2022b), Nsuamani et al. (2022), Thipwimonmas et al. (2021), Wang et al. (2021a), Zhang et al. (2021a), Dhiman et al. (2021), Patil et al. (2020), Forbes et al. (2020), Pollock et al. (2021)	Adegoke and Nic Daeid (2021), Neal et al. (2021), Junaid et al. (2022)
*Electrochemical*	A key theme was the use of 3D printing	Cardoso et al. (2020), Dutta et al. (2020), Zhu et al. (2022a), Apak et al. (2023), Arman et al. (2022), Dief et al. (2022), Mohan et al. (2021), Puttasakul et al. (2022), Li et al. (2020b), Tan et al. (2020), Urbanová and Pumera (2020), Pollock et al. (2021)	Nadar et al. (2021), Neal et al. (2021), Kushwaha et al. (2022), Castro et al. (2022), Shalini Devi et al. (2021)
*Immunoassay*	Papers focus on technologies such as ligand binding proteins, MIPs and AIE. The portability of these technologies is considered	Scorsone et al. (2021), Karadurmus et al. (2022), Paul et al. (2020)	Nadar et al. (2021), Yan et al. (2021)
*Luminescence*	A key theme was the use of dual detection	Gao et al. (2021), Scorsone et al. (2021), Qin et al. (2022a), Abuzalat et al. (2020), Batool et al. (2022a), Cao et al. (2022), Su et al. (2022), Zhang et al. (2022c), Zhu et al. (2022a), Bener et al. (2022), Chakraborty et al. (2022), Hao et al. (2021), Harathi and Thenmozhi (2022), Hu et al. (2023), Khan et al. (2021), Kumar et al. (2021), Li et al. (2023b), Nguyen et al. (2023), Noh et al. (2022), Pragya et al. (2022), Qin et al. (2022b), Santos et al. (2021), Praveen et al. (2020), Kathiravan et al. (2020), Li et al. (2020c), Lin et al. (2020), Paul et al. (2020), Patil et al. (2020), Thakarda et al. (2020), Wang et al. (2020a), Pollock et al. (2021), Mitri et al. (2022)	Nadar et al. (2021), Kumar (2021a), Rasheed et al. (2020), Verbitskiy et al. (2020), Neal et al. (2021), Junaid et al. (2022), Kushwaha et al. (2022)
*Raman*	Papers focussed on the design of SERS substrates such as SiO_2_-TiO_2_ Aerogel/Ag Flexible Films and Au/AgNPs	Vunckx et al. (2020), Gao et al. (2020), He et al. (2020a), Liu et al. (2020a), Raza et al. (2020), Vendamani et al. (2023), Liu et al. (2022b), Goel et al. (2022)	
*Other*	Continuous real-time monitoring, IR and LIBS are discussed as some of the technologies used	Díez-Pascual et al. (2022), Dreier et al. (2022), Kumar et al. (2020a), Saha et al. (2022), van Damme et al. (2023), Viola et al. (2023), Elbasuney et al. (2020b), Kumar et al. (2020a), Ricci and Gregory (2020), Santoro et al. (2020), Hung et al. (2022)	Senesi et al. (2021), Ricci and Gregory (2021)
Currently used by UK police and authorities			
Portable ion mobility, Raman and mass spectrometers are employed (ENFSI, 2021). Alongside, fluorescence imaging, colourimetric test kits and canine units (ENFSI, 2021; NPSA, 2025)			

Dual detection enhances sensitivity and selectivity, with Cao et al. (2022) proposing the combined use of colourimetric and fluorescent sensing with a carbon dots/titanyl sulfate (CDs/TiOSO_4_) sensing system for peroxides. Su et al. (2022) also demonstrate colourimetric sensing alongside luminescence using a Pt (II) terpyridyl complex-based sensing platform for perchlorate detection in water, soil and air. Molecularly imprinted polymers (MIPs) offer artificial recognition sites with a fluorescent composite of carbon dots (CDs) for on-site analysis (Nadar et al., 2021). The use of these dual detection methods limits the selectivity from other strong oxidants which could otherwise generate false positives (Cao et al., 2022).

3D printing rapidly produces electrodes for electrochemical sensing. For example, Cardoso et al. (2020) compared 3D printing pens and desktop printers for TNT detection. A key advantage of 3D printed electrodes is that new electrode surfaces can be generated by polishing thereby enabling reuse of the sensor–another advantage over chemically-modified electrochemical sensors (Cardoso et al., 2020). Urbanová and Pumera (2020) looked at the use of 3D printed titanium electrodes and Castro et al. (2022) completed an *ad hoc* review of 3D-printed electrochemical sensors showing the great promise they have for portable, on-site analysis.

Furthermore, continuous real-time monitoring of many explosives has been explored using a free-standing thin-film sensor relying on the catalytic decomposition of the explosive and its heat effects (Ricci and Gregory, 2021). Senesi et al. (2021) reviewed laser-induced breakdown spectroscopy (LIBS), a chemical elemental analysis technique which is found to be a sensitive and selective sensing technology suitable for on-site measurement.

In investigating crime scenes with potential explosives, UK police currently use a variety of on-site devices. Portable explosive detectors, such as ion mobility and mass spectrometers, offer rapid detection but face issues like high-cost, false positives from molecules with similar structures, sensitivity to environmental conditions like humidity and temperature and a limited range of detectable compounds, particularly newer or less common explosives (ENFSI, 2021). Canine units can be used with their high sensitivity to detect a range of explosive materials quickly (NPSA, 2025). Issues of high training costs, fatigue and distraction and the potential for false positives or false negatives due to masking odours (NIST, 2025). Colourimetric kits provide simple and immediate results but lack specificity and their storage and shelf-life can have a large impact on their effectiveness (ENFSI, 2021). Fluorescence and Raman spectroscopy provide non-destructive analysis utilising compounds unique spectral fingerprints. These technologies face limitations due to their complexity, high costs, the need for trained personnel and potential interference from other substances (ENFSI, 2021).

Recent research highlighted in the SR indicates promising advancements to address current challenges. Issues of high-cost, false positives and range of target analytes within current sensors are seen to be tackled by research within identified SR papers with dual detection gaining significant attention (Su et al., 2022; Nadar et al., 2021). Dual detection is also seen to provide an improvement to the lack of specificity associated with current colourimetric tests (Cao et al., 2022; Su et al., 2022). Additionally, the 3D printing of electrodes in electrochemical sensors is being explored to reduce the costs of current sensing technologies (Castro et al., 2022; Cardoso et al., 2020). Continuous real-time monitoring, as presented in the SR literature, offers a substantial improvement over current canine units by reducing costs and training time (Ricci and Gregory, 2021).

#### 3.2.7 Poison/toxin

Sensors for poison and toxin analysis are crucial for safeguarding health, protecting the environment and responding to hazardous material emergencies. 99 (13%) publications were concerned with poison/toxin analysis, with luminescence sensing technologies being the most commonly used (41% of papers, see Table 6). Key themes of eco-friendly materials, fast response and on-site technologies have been highlighted in identified papers with development of recognition elements playing a key role in achieving these.

**TABLE 6:** Summary of included publications relating to the use of sensors in poison or toxin analysis, alongside details of technologies currently used by UK police and authorities for on-site poison and toxin analysis.

Sensing technology	Description	Research papers	Review papers
*Colourimetric*	Papers focus on analytes from hazardous chemicals to nerve agents and environmental contaminants, with many in the gas phase. With a focus on the portability and rapid response of sensors	Rocher et al. (2021), Ahamed et al. (2023), Kumar (2021b), Zhao et al. (2022a), Chen et al. (2020b), Shin et al. (2020), Takahashi et al. (2021), María-Hormigos et al. (2022)	Kamel and Khattab (2020), Pena-Pereira et al. (2021)
*Electrochemical*	Key themes were the use of nanomaterials nanoparticles and magnetic nanoparticles and portability	Ataide et al. (2020), Noviana et al. (2019), Balaji et al. (2020), Kweitsu et al. (2021), Kim et al. (2020), Zhu et al. (2022a), Liu et al. (2022c), Kolacz et al. (2023), Ong et al. (2023), Santana et al. (2021), Yoo et al. (2022), Myadam et al. (2021), Montali et al. (2020), Nagabooshanam et al. (2021), Wang et al. (2020c)	Nadar et al. (2021), Kamel and Khattab (2020), Pena-Pereira et al. (2021), Castro et al. (2022), Shalini Devi et al. (2021), Kadu et al. (2023), Pohanka (2022)
*Immunoassay*	Key themes were the use of nanomaterials and group recognition	Meir et al. (2020), Chengyi et al. (2020), Mao et al. (2021b), Senra and Fonseca (2021), Pöhlmann and Elßner (2020), Tuccitto et al. (2020a), Tuccitto et al. (2020b), Wille and Elliott (2021), Lu et al. (2021), Wang et al. (2023a), Köse et al. (2021), Lan et al. (2020), Yao et al. (2020), Zhou et al. (2020)	Nadar et al. (2021), Yan et al. (2021), Nardo et al. (2021), Kamel and Khattab (2020), Mao et al. (2021a), Pena-Pereira et al. (2021), Kumar et al. (2020b), Nurazzi et al. (2021), Rary et al. (2020)
*Luminescence*	Key themes were the use of ecofriendly materials, dual recognition and gas sensing	Rocher et al. (2021), Su et al. (2021), Naik et al. (2020), Xue et al. (2021), Ahamed et al. (2023), Kumar (2021b), Zhu et al. (2022a), Liu et al. (2022c), Chen et al. (2022), Chen et al. (2023a), Das et al. (2022), Feng et al. (2023), Nugroho et al. (2023), Qiu et al. (2022), Suhasini et al. (2021), Wu et al. (2022a), Zhang et al. (2022d), Zhao et al. (2020a), Lian et al. (2020a), Harsha et al. (2020), Ke et al. (2020), Dagnaw et al. (2020), Ma et al. (2020), Montali et al. (2020), Pappalardo et al. (2020), Wu et al. (2020b), Xu et al. (2020), Xu et al. (2021), Yang et al. (2020a), Yang et al. (2020b), Yao et al. (2020), Yi and Zhang (2021)	Nadar et al. (2021), Kumar (2021a), Kamel and Khattab (2020), Pena-Pereira et al. (2021), Verhagen and Kelarakis (2020), Ganesan and Nagaraaj (2020), Tuccitto et al. (2020c), Hang et al. (2022)
*Raman*	Key themes were the use of novel materials quantum dots, nanomaterials, metal-organic frameworks, environmental monitoring and portability	Vunckx et al. (2020), Yang et al. (2021a), Balaji et al. (2020), Tay and Hulse (2021), Wille and Elliott (2021), Lu et al. (2021), Wang et al. (2023a), Atta and Vo-Dinh (2023), Liu et al. (2022d), Wu et al. (2022b), Xie et al. (2022b), Ding et al. (2022)	Hang et al. (2022), Zhang et al. (2022b)
*Other*	Key themes were the use of dual detection, environmental monitoring and nanomaterials	Dhar et al. (2021), Díez-Pascual et al. (2022), Viola et al. (2023), Liberatore et al. (2023), Zhu et al. (2022b), Guria et al. (2020), Kvachakhia et al. (2020), Rahman et al. (2020), Estevez et al. (2022)	Senesi et al. (2021)
Currently used by UK police and authorities			
Portable infrared spectrometers are currently used (Thermo Scientific, 2024). Additionally, biosensors (Naresh and Lee, 2021; Justino et al., 2017) based on electrochemical, optical, or piezoelectric principles, as well as multiplex immunoassay platforms (Mégarbane et al., 2020; Pöhlmann and Elßner, 2020), are employed for detecting a variety of substances			

The use of AIE phenomenon on filter paper strips detecting volatile poisons and pesticides has been demonstrated, though further research is needed for detecting poisons in body fluids and universal AIE probes for group recognition (Yan et al., 2021). A recognition element that detects classes of poisons would reduce sensing time and cost (Senra and Fonseca, 2021). An *ad hoc* review (Nardo et al., 2021) of LFIAs (also discussed above) details their use in the rapid on-site detection of viruses (Couturier et al., 2020; DeMers et al., 2020), toxins (Wu et al., 2020a; Li et al., 2020d; Bever et al., 2020; Pan et al., 2020; Xu et al., 2019), mycotoxins (Li et al., 2019a; Huang et al., 2020; Li et al., 2019b; Wang et al., 2021b; Wang et al., 2020b; Byzova et al., 2020), bacteria (Zhuang et al., 2020; Ilhan et al., 2021; He et al., 2019; Anfossi et al., 2018; Wang et al., 2019), allergens (Galan-Malo et al., 2019; Zhang et al., 2021b) and pesticides (Ge et al., 2020; Cevallos-Cedeño et al., 2021; Wu et al., 2019; Chen et al., 2020a). Paper-based on-site methods for pathogen detection using isothermal nucleic acid amplification are demonstrated (Dhar et al., 2021). Fast response time has been demonstrated using two fluorescent probe molecules (4-mercaptocoumarins) in a test strip to detect mustard gas and its analogues with a 3 min response time and high sensitivity (Xue et al., 2021).

Another key theme was sensing of gaseous target analytes. Shin et al. (2020) developed a sensitive colorimetric gas sensor with a smartphone-based analysis for real-time quantitative detection of bacterial-derived ammonia gas, helping determine the *postmortem* interval (PMI). Such on-site sensing overcomes previous challenges of bacterial growth whilst the body is being moved which can produce false PMIs. Multiplexed gas sensing is discussed with several papers looking at the use of bifunctional fluorescent probes. These probes, equipped with two sensing sites, yield two distinct fluorescence responses upon exposure to either of the two target analytes, ranging from various nerve agent stimulants to mustard gas and phosgene (Feng et al., 2023).

The detection of poisons and toxins to combat crime presents a wide range of target analytes. UK police and other emergency responders currently employ a broad range of technologies for rapid and accurate on-site detection. Portable spectrometers such as Thermo Scientific’s TruDefender and FirstDefender use infrared spectroscopy but are costly and complex (Thermo Scientific, 2024). Biosensors based on electrochemical, optical or piezoelectric principles are common in use providing rapid results (Naresh and Lee, 2021; Justino et al., 2017). Challenges such as environmental instability (fluctuations in pH, humidity, and temperature), interference from complex sample matrices, and a limited detection range persist when attempting to simultaneously detect multiple analytes (Justino et al., 2017). Multiplex immunoassay platforms are also used allowing simultaneous detection of multiple toxins in a single sample by using antibodies to bind to specific toxins making them highly specific. However, there are issues of interference and a limited detection range with many potential unknown target analytes (Mégarbane et al., 2020; Pöhlmann and Elßner, 2020).

Recent research highlighted in the SR indicates promising advancements to address current challenges. Current issues such as high costs and a limited range of detectable target analytes are being tackled by papers identified within the SR focussing on the use of AIE probes for group recognition (Yan et al., 2021) and cost-effective paper-based methods (Dhar et al., 2021). The development of these technologies is expected to significantly impact not only poison and toxin detection but also the detection of other target analytes.

#### 3.2.8 Fingerprints

Fingerprint analysis is a key tool in modern law enforcement, providing reliable and efficient identification of individuals. 108 (14%) publications concerned fingerprint analysis, with luminescence sensing technologies being the most commonly used (79% of papers, see Table 7). Key themes include the development of environmentally friendly, non-toxic sensors and the use of nanomaterials for enhanced fingerprint sensing.

**TABLE 7:** Summary of included publications relating to the use of sensors in fingerprint analysis, alongside details of technologies currently used by UK police and authorities for on-site fingerprint analysis.

Sensing technology	Description	Research papers	Review papers
*Colourimetric*	Papers focus on the application of nanomaterials, such as graphene-isolated nanocrystals, hierarchical structures emitting colours, and fluorescent-labelled nanohybrids	Azman et al. (2021), Rajan et al. (2020), Wang et al. (2022a), Dhiman et al. (2021), Costa et al. (2020), Dare et al. (2020), Prasad et al. (2021), Lin et al. (2022), Rasin et al. (2023), Bhagat et al. (2019), Divya et al. (2020), Kumar and Singh (2023)	Nadar et al. (2021), Sharma et al. (2021)
*Electrochemical*	Electrochemical paper-based devices are reviewed alongside the use of nanomaterials and polymers	Ataide et al. (2020), Wei and Cui (2021), Liu et al. (2023b), Malik et al. (2020), Hu et al. (2020a)	Nadar et al. (2021)
*Immunoassay*	Papers look at the combination of multiple advanced techniques and renewable materials such as Chitosan	Tiedge et al. (2020), Vuckovic et al. (2021)	Nadar et al. (2021)
*Luminescence*	Key themes were the use of nanomaterial advances, dual detection and eco-friendly materials	Ding et al. (2021a), Mengjun and Peng (2021), Pattarith and Benchawattananon (2020), Lee et al. (2022b), Rajput and Shishodia (2020), Shabashini et al. (2021), Shahbazi et al. (2020), Trabelsi et al. (2021), Wang et al. (2020d), Wang et al. (2020e), Yuan et al. (2020), Zhao et al. (2020b), Gonzalez et al. (2020), Nugroho et al. (2023), Fan et al. (2023), Abdollahi et al. (2022), Ansari et al. (2022), Li et al. (2022a), Narasimhamurthy et al. (2021), Ravindra et al. (2021), Cai et al. (2022b), Duan et al. (2022a), Ghubish and El-Kemary (2022), Jindal and Kaur (2023), King et al. (2021), Latha et al. (2021), Lavanya et al. (2022), Lee et al. (2022b), Li et al. (2021a), Li and Peng (2022), Machado et al. (2022), Karimi-Maleh et al. (2020), Nugroho et al. (2022a), Nugroho et al. (2022b), Peng et al. (2022), Peng and Zhao (2023), Pramanik et al. (2022), Qiu et al. (2021), Ran et al. (2022), Renuka et al. (2021), Sekar et al. (2021), Singh et al. (2022a), Swathi et al. (2023), Vadivel et al. (2021), Wan et al. (2022), Wang et al. (2022b), Yu et al. (2022b), Yu et al. (2023a), Yuan et al. (2022a), Zhang et al. (2021c), Zhu et al. (2022c), Zou et al. (2022a), Fouad and Saif (2020), Guleria et al. (2020), Kamal and Saif (2020), Suresh et al. (2020), Ghubish et al. (2020), Hu et al. (2020a), Jenie et al. (2020), Kumar et al. (2020c), Lai et al. (2020), Navami et al. (2020), Olszowska et al. (2020), Pavitra et al. (2020), Prabakaran and Pillay (2020a), Prabakaran and Pillay (2020b), Liu and Zhang (2020), Dwivedi et al. (2023), Firmino et al. (2021), Illston-Baggs et al. (2021), Kanodarwala et al. (2021), You et al. (2021)	Nadar et al. (2021), Verhagen and Kelarakis (2020), Lian et al. (2020b), Prasad et al. (2020), Sharma et al., 2021, Pinto et al. (2022), Prabakaran and Pillay (2021), Shahbazi et al. (2022)
*Raman*	Papers look at the self-assembly of nanocrystals onto lipidic ridge details provides a dual-sensing technique and a the miniaturisation of Raman spectrometers	Vunckx et al. (2020), Wang et al. (2022a)	
*Other*	Papers look at the use of nanomaterials and doping to enhance detection and an *ad hoc* review of various fingerprint development techniques currently used	Liang et al. (2020), Assis et al. (2023), Bhati et al. (2023), Bumbrah et al. (2022), Dahiya et al. (2022), Fouda-Mbanga et al. (2022), Fouda-Mbanga et al. (2023), Bouaziz et al. (2022)	Ferreira et al. (2021a)
Currently used by UK police and authorities			
MobileID Devices are currently used to collect on-site fingerprints, with the IDENT1 System enabling real-time identification (Fingerprint Identification, 2024; Mobileidworld, 2013; Press Release, 2018; IDEMIA, 2025; Neurotechnology, 2025; Fingerprint Identification, 2024; Mobileidworld, 2013; Press Release, 2018; IDEMIA, 2025; Neurotechnology, 2025). Various powders and lifting techniques are currently employed to develop latent prints on-site including aluminium, magna flake, black granular, and black magnetic powders (Bandey and Gibson, 2006) (NCJRS Virtual Library, 2001; Defence Science and Technology Laboratory, 2022)			

The development of environmentally friendly, non-toxic sensors was a key issue identified for mass production. Azman et al. (2021) proposed using a lipase from *Candida* rugosa (CRL) as a greener option for fingerprint analysis on wet substrates. Although CRL is used in various scientific applications (Prlainović et al., 2013; Che Marzuki et al., 2015; Mohamad et al., 2015), its use in fingerprint visualisation is limited, presenting an exciting research opportunity.

Recent advances in the use of nanomaterials for sensing can be applied to fingerprint sensing for many benefits. A review of magnetic nanoparticles (MNPs) to conjugate with quantum dots (QDs) for fluorescence properties has been presented (Nadar et al., 2021). Unlike traditional powders used, the small size of MNPs were found to be efficient in selectively binding to fingerprints and not the background. Gold nanoparticles on fibrous nano-silica enhance ridge details and sweat pores on various surfaces with properties of low-cost, easy preparation, chemical stability and great affinity to finger residues (Wei and Cui, 2021). Ansari et al. (2022) review applied nanomaterials and luminescent Ln^3+^ NPs/upconversion (UC) NPs which provide higher contrast, sensitivity and selectivity which is lacking in most of the traditional fluorescent nanomaterials used. However, more studies are needed to improve the efficiency, performance, surface-functionality and biocompatibility of these Ln^3+^ NPs/UCNPs for fingerprint recognition.

Carbon dots (CDs) present exceptional characteristics such as high fluorescence, non-toxicity, eco-friendliness, stability and cost-effectiveness compared to traditional methods. Shabashini et al. (2021) present an *ad hoc* review of publications relating to the application of CDs but of importance here is the enhancement of fingerprint visualisation, using magnetic composite powder CDs, due to the abundant surface hydrophilic groups (Ding et al., 2021a). Low-cost, superparamagnetic fluorescence performance and excellent safety makes these CDs suitable candidates for on-site visualisation. However, issues remain to transition from proof of concept to field application.

For on-site fingerprint detection the UK police use various mobile biometric devices to capture fingerprints and perform identity checks in real-time (Press Release, 2018). NEC MobileID (Fingerprint Identification, 2024) allows officers to capture fingerprints while Cross Match SEEK Avenger (Mobileidworld, 2013) captures fingerprints, iris scans and facial images. Both devices enable real-time identification cross-referencing with the UK’s central fingerprint databases (e.g., IDENT1 System (Press Release, 2018)). Fixed systems in police stations, like Morpho Livescan by IDEMIA (IDEMIA, 2025) and Crossmatch L SCAN (Neurotechnology, 2025), offer higher quality capturing. However, the use of on-site detection is preferable to reduce trips to and from police stations improving efficiency (Press Release, 2018). Many kits are currently used for developing latent fingerprints at crime scenes. These include powders and lifting tapes, which are then analysed further in a lab or using mobile devices with Automated Fingerprint Identification System (AFIS) access. The choice of powder depends on the surface, with common types being aluminium, magna flake, black granular, and black magnetic powders (Bandey and Gibson, 2006). Traditional powder lifting technologies face limitations, including sensitivity to environmental conditions, surface compatibility, DNA degradation due to chemicals used and the need for extensive training to ensure proper application and interpretation (NCJRS Virtual Library, 2001; Defence Science and Technology Laboratory, 2022).

Recent research highlighted in the SR indicates promising advancements to address current challenges. Improvements to MobileID devices were not captured within this SR. A key theme in the papers identified through this SR was the use of nanomaterials instead of traditional powders for fingerprint detection. These nanomaterials offer enhanced sensitivity and selectivity, overcoming issues related to environmental conditions (Ansari et al., 2022; Nadar et al., 2021). However, further research is needed to improve surface functionality and biocompatibility to transition these innovations from proof of concept to widespread field application (Ansari et al., 2022; Shabashini et al., 2021; Ding et al., 2021a).

#### 3.2.9 Food safety

For food safety, sensors are essential for maintaining the integrity of the supply chain, protecting public health and ensuring compliance with regulations. 168 (22%) publications concerned food safety, with immunoassay sensing technologies being the most commonly used (36% of papers). The remaining papers were represented by a fairly even spread of sensing technologies (Table 8). Key themes discussed include the use of dual detection, nanomaterials and portability.

**TABLE 8:** Summary of included publications relating to the use of sensors in food safety analysis, alongside details of technologies currently used by UK police and authorities for on-site food safety analysis.

Sensing technology	Description	Research papers	Review papers
*Colourimetric*	Key themes were the use of dual detection (particularly immunochromatographic assays), nanomaterials, portability, labelling and metal-organic frameworks	Chu et al. (2020), Li et al. (2020e), Dai et al. (2022), Zhou et al. (2021), Wu et al. (2022c), Tortajada-Genaro et al. (2022), Fang et al. (2023), Qin et al. (2022c), Zhang et al. (2022e), Zhao et al. (2022b), Amalraj et al. (2022), Baghban et al. (2022), Ranbir et al. (2023), Tasangtong et al. (2023), Malahom et al. (2020)	Kamel and Khattab (2020), Hua et al. (2021), Li et al. (2020f), Cheng et al. (2021), Zhou et al. (2022a)
*Electrochemical*	Key themes were the use of eco-friendly materials paper-based and nanomaterials	Ataide et al. (2020), Noviana et al. (2019), Hu et al. (2021), Ngo et al. (2020), Li et al. (2020e), Mansouri et al. (2020), Rocha et al. (2020), Roushani et al. (2021), Saichanapan et al. (2020), Shishkanova et al. (2021), Wang et al. (2021c), Xie et al. (2020), Zhang et al. (2020b), Sfragano et al. (2020), Nagabooshanam et al. (2021), Dai et al. (2022), Yu et al. (2022a), Han et al. (2023), Shao et al. (2023), Kushwaha et al. (2022), Hakeem et al. (2022), Singh et al. (2021), Bai (2022), Carvalho et al. (2023), Geng et al. (2023), Gurusamy et al. (2022), Jin et al. (2023), Joao et al. (2021), Lei et al. (2022), Mei et al. (2022), Qader et al. (2022), Sun et al. (2023), Sundhoro et al. (2021), Torrarit et al. (2022), Wu et al. (2022d), Zhou et al. (2022b)	Kamel and Khattab (2020), Li et al. (2020f), Karimi-Maleh et al. (2020), Cheng et al. (2021), Zhou et al. (2022a), Gaudin (2021), Castro et al. (2022), Ferrari et al. (2021), Mahmudiono et al. (2022)
*Immunoassay*	Key themes were the use of dual detection, eco-friendly materials and multiplexing	Yao et al. (2020), Wu et al. (2021a), Duan et al. (2020), Na et al. (2020), He et al. (2021), Li et al. (2020e), Peng et al. (2020), Rebelo et al. (2021), Roushani et al. (2021), Zhang et al. (2020c), Suryoprabowo et al. (2021), Wang et al. (2021c), Wu et al. (2020c), Li et al. (2020g), Zhang et al. (2020d), Zinna et al. (2020), Moon et al. (2020), Lan et al. (2020), Zhou et al. (2020), Dai et al. (2022), Zhou et al. (2021), Wu et al. (2022c), Tortajada-Genaro et al. (2022), Fang et al. (2023), Qin et al. (2022c), Zhang et al. (2022e), Yu et al. (2022a), Han et al. (2023), Shao et al. (2023), Duan et al. (2023), Guan et al. (2022), Ling et al. (2022), Liu et al. (2022e), Pan et al. (2022), Wen et al. (2023), Xiao et al. (2022), Yang et al. (2023a), Duan et al. (2022b), Neng et al. (2023), Behyar and Shadjou (2021), Chen et al. (2023b), Jamieson et al. (2021), Li et al. (2022c), Li et al. (2022d), Luo et al. (2022), Ouyang et al. (2022), Peltomaa et al. (2022), Maashi (2023), Wang et al. (2022c), Wang et al. (2022d), Xiong et al. (2022a), Malahom et al. (2020), Zhang et al. (2020e)	Kamel and Khattab (2020), Hua et al. (2021), Karimi-Maleh et al. (2020), Nardo et al. (2021), Kumar et al. (2020b), Gaudin (2021), Uttpal et al. (2022), Bräuer et al. (2021)
*Luminescence*	Key themes were the use of nanomaterials, doping for enhanced sensing, dual detection and eco-friendly materials	Lian et al. (2020a), Yao et al. (2020), Yi and Zhang (2021), Badshah et al. (2020), Wu et al. (2021a), Jalili and Khataee (2020), Li et al. (2020e), Li et al. (2020h), Liao et al. (2021), Monago-Maraña et al. (2020), Suryoprabowo et al. (2021), Wang et al. (2020f), Wu et al. (2020c), Zhang et al. (2020d), Zhou et al. (2021), Harsha et al. (2020), Dai et al. (2022), Zhao et al. (2022b), Kushwaha et al. (2022), Duan et al. (2023), Guan et al. (2022), Ling et al. (2022), Liu et al. (2022e), Pan et al. (2022), Wen et al. (2023), Xiao et al. (2022), Yang et al. (2023a), Jindal and Kaur (2023), Anjali et al. (2022), Basterrechea et al. (2021), Hu et al. (2022a), Li et al. (2021b), Li et al. (2021c), Li et al. (2022e), Li et al. (2022f), Teng et al. (2023), Wang et al. (2022e), Wu et al. (2021b), Xie et al. (2022c), Xiong et al. (2022b), Yi and Zhang (2021), Zhou et al. (2022c), Zhou et al. (2023), Guo et al. (2020), Guo et al. (2021)	Kamel and Khattab (2020), Tao et al. (2020), Cheng et al. (2021), Zhou et al. (2022a), Uttpal et al. (2022)
*Raman*	Key themes were the use of dual detection and portability	Vunckx et al. (2020), Cheng et al. (2020), Cupil-Garcia et al. (2020), Duan et al. (2020), He et al. (2020b), Jing et al. (2021), Li et al. (2020e), Neng et al. (2020), Sha et al. (2020), Wu et al. (2020d), Li et al. (2020g), Wu et al. (2022c), Duan et al. (2022b), Neng et al. (2023), Vendamani et al. (2023), Goel et al. (2022), Cai et al. (2022a), Chang et al. (2022a), Alomar et al. (2022), Barveen et al. (2021), Chang et al. (2022b), Chen et al. (2023c), Huang et al. (2022b), Huang et al. (2022c), Jiang et al. (2021b), Jing et al. (2021), Li et al. (2022g), Lu et al. (2022), Mao et al. (2023), Sun et al. (2021), Wei et al. (2021), Yang et al. (2021b), Yang et al. (2022a), Yang et al. (2022b), Yang et al. (2022c), Yang et al. (2023b), Yu et al. (2022c), Yuan et al. (2022b), Zhu et al. (2021)	Cheng et al. (2021), Xie et al. (2022a), Girmatsion et al. (2021), Zhang et al. (2022b)
*Other*	Papers present the use of IR for rapid contaminant detection, graphene oxide-Fe_3_O_4_ for extraction of illegal dyes and an *ad hoc* review of MOFs in food safety sensors	Fulgencio et al. (2022), Sha et al. (2021)	Hitabatuma et al. (2022)
Currently used by UK police and authorities			
Current on-site detection is carried out by authorities using portable spectrometers (i.e., Raman (TRUSCANRM, 2025) and IR (PerkinElmer, 2025)), Rapid testing kits (Hygiena, 2025; Food Allergen Testing, 2025; Merck, 2025; Hygiena, 2025; Food Allergen Testing, 2025; Merck, 2025) (i.e., colourimetric and lateral flow immunoassays) and UV lights (Coleparmer, 2025)			

Tasangtong et al. (2023) discuss inkjet-printed paper devices for rapid, portable and eco-friendly formaldehyde analysis in foods. Similarly, 3D-printing of graphene-polylactic acid electrodes for atropine detection in beverages offers low-cost, reproducible, large-scale sensor production and shows great promise for developing other electrochemical sensors for analytes commonly found at crime scenes (Joao et al., 2021).

Development of dual detection sensors was seen as key across publications. Roushani et al. (2021) discuss a double recognition strategy using MIP and aptamer on a carbon electrode to sense ractopamine, a molecule commonly used in livestock feed, sometimes inappropriately or excessively. This strategy can be extended to other target analytes by the simple exchange of the relevant aptamer. The paper also highlights antibiotic degradation detection in milk using silver nanoparticle-decorated TiO_2_ for solid-phase microextraction (SPME) and SERS (Jing et al., 2021). SPME is a new sample preparation technique that simplifies extraction and reduces sample loss. Silver nanoparticles have greater SERS activity than traditional silver sol. In combination, SPME-SERS provides rapid on-site detection.

Antibody-based sensing technologies are common in food safety analysis offering low-cost and rapid detection. Development of a fluorescent immunochromatographic strip assay based on a chlorpheniramine (CPM) antibody in the detection of CPM, a harmful illegal additive in teas and health foods is discussed (Zhou et al., 2021). A LFIA based on a fluorescence and gold nanoparticles labelled antibody for Tadalafil (a banned additive found in beverages) recognition is also presented (Suryoprabowo et al., 2021). The strip is observed under ultra-violet light and can be completed within 10-min making it perfect for on-site analysis.

The responsibility of food safety analysis tends to rest with local authorities such as environmental health officers (EHOs) and the Food Standards Agency (FSA). With many potential target analytes there are a vast array of technologies currently employed. These include portable spectrometers such as Raman spectrometers (TRUSCANRM, 2025) and IR spectrometers (PerkinElmer, 2025) to identify contaminants and potential adulterants in food products. These products present issues of initial high investment costs and personnel training with the need for special sample preparation for some IR readings. Rapid testing kits, like ATP (adenosine triphosphate) Testing Kits (Hygiena, 2025) that measure cleanliness and Lateral flow devices (LFDs) (Food Allergen Testing, 2025) for rapid, on-site pathogen detection, often face issues of specificity which can result in false positives or negatives. Chemical test strips detect residues such as pesticides, heavy metals, and other contaminants but often suffer from limited sensitivity, provide only semi-quantitative results and require specific storage conditions (Merck, 2025). This is due to their small surface area and reaction zone which restrict the amount of analyte that can interact with reagents, and the absence of amplification steps commonly used in lab-based methods. UV lights are used to check hygiene standards and detect contamination with biological residues but these only work on smoother non-porous surfaces and require additional safety precautions due to potential prolonged UV exposure (Coleparmer, 2025).

Recent research highlighted in the SR indicates promising advancements to address current challenges. Issues with the high cost and extensive training requirements for current spectrometers are being addressed by developing dual detectors (Roushani et al., 2021; Jing et al., 2021). Some identified dual detectors achieve similar levels of selectivity but require further development to match the sensitivity of current devices. Additionally, inkjet and 3D printed electrochemical devices are being researched as cost-effective, mass-producible alternatives (Tasangtong et al., 2023; Joao et al., 2021). Improvements in rapid testing kits also focus on dual detection, enhancing both specificity and sensitivity (Roushani et al., 2021; Zhou et al., 2021; Suryoprabowo et al., 2021).

#### 3.2.10 Illicit drugs

Sensors play a pivotal role in illicit drug analysis for law enforcement, forensic investigations, public health and safety, enabling their identification, detection, and monitoring. 281 (36%) publications concerned illicit drug analysis, with electrochemical sensing technologies being the most popular (49% of papers). Colourimetric, immunoassay, luminescence and Raman sensing technologies represent an even share of the remaining publications. As shown in Table 9, the detection of some illicit drugs (e.g., stimulants) has received more attention than others (e.g., hallucinogens). The vast array of technologies explored for illicit drug analysis presents numerous opportunities for further research. Consequently, the key themes identified in the literature are summarized below.

**TABLE 9:** Summary of included publications relating to the use of sensors in illicit drug analysis, alongside details of technologies currently used by UK police and authorities for on-site illicit drug analysis.

Sensing technology	Drug category detected	Description	Research papers	Review papers
*Colourimetric*	Depressants	Papers look at eco-friendly materials, how to overcome interferants, portability and the use of nanomaterials	Hu et al. (2020b), Li (2020), Ryu and Kim (2022), Ha et al. (2022), Kaewnu et al. (2021), Mustafa et al. (2021), Son et al. (2021)	
	Stimulants	Papers look at portability, material innovation (e.g. metal nanoparticles), targeted detection but also the manipulation of developed technologies to detect other target analytes	Silva and da Paixao (2022), Lin et al. (2023a), Cho and Kim (2022), Jang et al. (2022), Jornet-Martinez et al. (2021), Ameku et al. (2021), Adegoke et al. (2020)	Khorablou et al. (2021), Ahmed et al. (2020)
	Hallucinogen	—		
	Pharmaceuticals	Papers look at novel detection materials such as Au@Ag core-shell nanoparticles and carbon quantum dots alongside smartphone-based assay detection	Shkembi et al. (2022), Madani-Nejad et al. (2023), Chu et al. (2020)	
	Dissociatives	—		
	Cannabinoids	Papers have focussed on portability of affinity assays and the use of ionogel-based materials	Puiu and Bala (2022), Catalan-Carrio et al. (2021)	
	Opioids	Papers look at the use of recognition elements for charge transfer colourimetric detection and two ad hoc reviews are presented on advances in nanobiosensors for opioids and biosensing for rapid illicit drug detection	Lin et al. (2021)	Razlansari et al. (2022), Ahmed et al. (2020)
	Other	Papers have focussed on ad hoc reviews with key themes of portability and nanomaterials (magnetic nanoparticles, nanoplates and nanocomposites)		Kamel and Khattab (2020), Pena-Pereira et al. (2021), Chen et al. (2020c), Nadar et al. (2021), Neal et al. (2021), Anzar et al. (2022), Dagar et al. (2022)
*Electrochemical*	Depressants	Key themes were the use of graphene-based materials (enhancing sensitivity and selectivity), nano- and 2D- materials, portability and dual detection	Jiang et al. (2023), Boroujerdi and Paul (2022a), de Lima and de Araujo (2022), Garima et al. (2022a), Mutz et al. (2022), Olean-Oliveira et al. (2023), Paschoarelli et al. (2023), Rocha et al. (2021a), Zhang et al. (2022a), Boroujerdi et al. (2020), Papadopoulos et al. (2020), Sohouli et al. (2020)	Davis-Martin et al. (2021), Brown and Dennany (2022)
	Stimulants	Key themes were the improvement of sensitivity and selectivity, portability, dual detection (particularly with MIPs) and nanomaterials	Silva and da Paixao (2022), Hu et al. (2022b), Jiang et al. (2023), Atik et al. (2023), El-Akaad et al. (2021), Li et al. (2022h), Sposito et al. (2022), Wu et al. (2023), Xie et al. (2022d), Dokuzparmak et al. (2021), Takahashi et al. (2022), Abd-Elsabour et al. (2022), Abnous et al. (2022), Anvari et al. (2023), Borgul et al. (2022a), Borgul et al. (2022b), Castro et al. (2021), de Faria et al. (2022), Ghorbanizamani et al. (2022), González-Hernández et al. (2022a), Saisahas et al. (2022b), Lee et al. (2022c), Li et al. (2021d), Novais et al. (2022), Papaioannou et al. (2022), Pospisilová et al. (2023), Schram et al. (2022), Vargas et al. (2022), Wang et al. (2021d), Xu et al. (2022), Zhang et al. (2022f), Zhou et al. (2022d), Borgul et al. (2021), Ameku et al. (2021), Tan et al. (2022), Soni et al. (2022a), Couto et al. (2021), Liu et al. (2020b), Ruchala et al. (2021), Rocha et al. (2021b), Akgönüllü et al. (2020), Anvari et al. (2021), Haghighi et al. (2020), Lima et al. (2020), Nayini et al. (2020), Ott et al. (2020), Shishkanova et al. (2020), Yang et al. (2021c)	Khorablou et al. (2021), Brown and Dennany (2022), Almabadi et al. (2023), Bilge et al. (2022), Boroujerdi and Paul (2022b), Ahmed et al. (2020)
	Hallucinogen	Key themes were the use of dual detection (particularly MIPs) and portability	Jiang et al. (2023), Li et al. (2022), Soni et al. (2022a), Vargas et al. (2022), Silva et al. (2021), Van Echelpoel et al. (2022), Van Echelpoel et al. (2023), Ribeiro et al. (2020), Amr et al. (2021)	Brown and Dennany (2022)
	Pharmaceuticals	Key themes were the use of nanomaterials, eco-friendly materials, portability and improved sensitivity and selectivity	Sposito et al. (2022), Sachdeva et al. (2021), Sanguarnsak et al. (2022), Borgul et al. (2021), Boroujerdi et al. (2022a), Dias et al. (2021), Düzmen and Aslanoglu (2022), Elbalkiny and Samir (2022), Kadhim et al. (2023), Pogăcean et al. (2022), Saisahas et al. (2022a), Wahba et al. (2023), Promsuwan et al. (2020b)	Mostafa et al. (2022)
	Dissociatives	Papers focus on the detection of ketamine and other “date rape” drugs. Looking at MIPs, nanoparticles and the reusability and portability of devices	Soliman et al. (2023), Rocha et al. (2021a), Zou et al. (2022b)	
	Cannabinoids	Papers look at the problem of novel cannabinoid detection and how their constant development impacts their detection alongside the use of screen-printed electrodes with nanoparticles and carbon nanotubes	Puiu and Bala (2022), Jiang et al. (2023), Ameen et al. (2022), Brenes et al. (2022), Pholsiri et al. (2023), Ameen et al. (2020), Klimuntowski et al. (2020)	Brown and Dennany (2022), Amini et al. (2022)
	Opioids	Key themes were the use of multiplexing (simultaneous detection of multiple drugs), issues of interferents within samples, disposable sensors and an ad hoc review on current and future perspectives on opioid sensors	Borgul et al. (2022c), González-Hernández et al. (2022b), Khorablou et al. (2022), Mousaabadi et al. (2022), Ortiz-Aguayo et al. (2022), Singh et al. (2022b), Wang et al. (2023b), Yang et al. (2022d)	Glasscott et al. (2020), Choinska et al. (2022), Ahmed et al. (2020)
	Other	Key themes were the use of dual detection, printing of cells (paper and 3D printing), portability, nanomaterials and multiplexing	Yu et al. (2023b), Ferreira et al. (2021b), Joosten et al. (2022), Parrilla et al. (2021), Parrilla et al. (2022), Poulladofonou et al. (2022), Zamani and Yamini (2023), Ceto et al. (2022), Sanli et al. (2020), Brown et al. (2020), Ataide et al. (2020), Noviana et al. (2019), Rocha et al. (2020), Shishkanova et al. (2021), Amr et al. (2021), De Rycke et al. (2020), Dragan et al. (2021), Ferrari et al. (2020), Li et al. (2021e), Masemola et al. (2020), Zanfrognini et al. (2020), Montali et al. (2020)	Kamel and Khattab (2020), Pena-Pereira et al. (2021), Nadar et al. (2021), Karimi-Maleh et al. (2020), Mani et al. (2021), Amiri et al. (2021), Teymourian et al. (2020), Neal et al. (2021), Anzar et al. (2022), Dagar et al. (2022), Castro et al. (2022), Dai (2023), Ren et al. (2021), Su (2022)2
*Immunoassay*	Depressants	Papers look at the use of MIPs and carbon-dot technologies alongside the development of wearable sensors for continuous monitoring	Hu et al. (2020b), Yen et al. (2020), Jiang et al. (2023)	
	Stimulants	Key themes were the use of dual detection (with electrochemical and fluorescent/ optical technologies), multiplexing (derivatisation-assisted immunoassay for group-specific detection) and portability	Lin et al. (2023a), Hu et al. (2022b), Jiang et al. (2023), Atik et al. (2023), El-Akaad et al. (2021), Li et al. (2022h), Sposito et al. (2022), Wu et al. (2023), Xie et al. (2022d), Esmaelpourfarkhani et al. (2023), Paul et al. (2021), Zamanian et al. (2022), Geng et al. (2022), Chen et al. (2021a), Morita et al. (2022), Zhao et al. (2022c), Tan et al. (2022), Soni et al. (2022a), Couto et al. (2021), Liu et al. (2020b), Grothe et al. (2021)	Lal et al. (2022)
	Hallucinogen	Papers look at nanomaterial modification such as incorporation of graphene oxide for enhanced sensor performance, conductivity and stability. Selective detection of the target analyte was also covered for accurate and reliable detection in complex samples	Jiang et al. (2023), Li et al. (2022), Soni et al. (2022a)	
	Pharmaceuticals	Papers focus on dual detection with nandrolone aptamers for colourimetric detection (steroids), MIPs for electrochemical sensing of diltiazem (high blood pressure treatment) and fluorescence polarisation immunoassay for amitriptyline (antidepressant)	Shkembi et al. (2022), Sposito et al. (2022), Medyantseva et al. (2022)	
	Dissociatives	Looks at the use of core-shell MIPs for on-site selective determination of ketamine	Soliman et al. (2023)	
	Cannabinoids	Papers look at the use of nanomaterials such as nanocellulose and graphene oxide	Jiang et al. (2023), Solin et al. (2023), Balaban et al. (2020)	
	Opioids	Papers look at multiplexed detection for fentanyl and its analogues and for multiple narcotics or explosives at once using a biosensor array. Papers also examine dual detection and detection in human matrices, specifically highlighting the utilization of Surface-Enhanced Raman Spectroscopy (SERS) in conjunction with an immunochromatographic assays for morphine detection in saliva	Canoura et al. (2023), Scorsone et al. (2021), Li et al. (2020)	
	Other	Key themes were dual detection, eco-friendly materials (enzyme-based paper tests), drug monitoring in waste-water and interferents	Yan et al. (2022), Gozdzialski et al. (2023), Sanli et al. (2020), Wu et al. (2021a), Zhang et al. (2020d), Wille and Elliott (2021), Zinna et al. (2020), Senra and Fonseca (2021), Garcia-Cruz et al. (2020), Badawy (2020), Suherman et al. (2020), Truta et al. (2020)	Kamel and Khattab (2020), Pena-Pereira et al. (2021), Nadar et al. (2021), Karimi-Maleh et al. (2020), Nardo et al. (2021), Mao et al. (2021a), Rary et al. (2020), Mao et al. (2020), Santillo (2020), Bräuer et al. (2021), Soni et al. (2022b)
*Luminescence*	Depressants	Papers look at advanced materials (nanostructured photonic hydrogels, AIE active fluorene-containing compounds, and graphene materials), on-site monitoring and specificity towards intended target analytes	Ryu and Kim (2022), Ahmed et al. (2022), Dahiwadkar et al. (2022), Garima et al. (2022b), Garrido et al. (2023)	Brown and Dennany (2022)
	Stimulants	Key themes were dual detection (quantum dots, aptamers, metal-organic frameworks), ultrasensitive detection and novel materials	Hu et al. (2022b), Dokuzparmak et al. (2021), Takahashi et al. (2022), Esmaelpourfarkhani et al. (2023), Paul et al. (2021), Zamanian et al. (2022), Adegoke et al. (2023), Ding et al. (2021b), Elmizadeh et al. (2023), Guo et al. (2022a), Tan et al. (2022), Ruchala et al. (2021), Dokuzparmak et al. (2021), Jeong et al. (2020), Wu et al. (2020e), Zhang and Yan (2020), Zhao et al. (2020b), Mittal et al. (2020)	Khorablou et al. (2021), Brown and Dennany (2022)
	Hallucinogen	Papers look at the use of silica nanoparticles loaded with fluorescent dye for detection of novel psychedelic drug and an ad hoc review is presented on the use of electrochemiluminescence sensors for drug detection	Garrido et al. (2020)	Brown and Dennany (2022)
	Pharmaceuticals	Key themes were dual detection, nanomaterials, specificity, portability (through miniaturisation) and functionalised surfaces	Sachdeva et al. (2021), Sanguarnsak et al. (2022), Medyantseva et al. (2022), Adegoke et al. (2022), Al-Hetlani et al. (2020)	
	Dissociatives	—		
	Cannabinoids	Papers look at the portability of affinity assays and the screening of samples in herbal mixtures (interferents present) with gold nanoclusters	Puiu and Bala (2022), Yen et al. (2022a)	Brown and Dennany (2022)
	Opioids	Papers look at the use of nanobiosensors with nanomaterials and bio-recognition elements for high sensitivity and selectivity alongside considering interferents that will be found in real samples and the potential for multiplexing	Yan et al. (2023), Scorsone et al. (2021), Alhaddad and Sheta (2020)	Razlansari et al. (2022)
	Other	Key themes were portability and nanomaterials	Yu et al. (2023b), Yan et al. (2022), Warning et al. (2021), Chen et al. (2023d), Pal et al. (2023), Wei et al. (2022), Yen et al. (2022b), Brown et al. (2020), Wu et al. (2021a), Zhang et al. (2020d), Montali et al. (2020)	Kamel and Khattab (2020), Pena-Pereira et al. (2021), Nadar et al. (2021), Neal et al. (2021), Anzar et al. (2022), Dagar et al. (2022), Hang et al. (2022), Loch et al. (2023)
*Raman*	Depressants	Papers look at nanomaterials, discussing nanosheets and nanocluster formation, alongside the need for rapid and sensitive detection	Chen et al. (2021b), Sha et al. (2020), Açikgöz and Hamamci (2020), Wang et al. (2021e)	
	Stimulants	Key themes were nanomaterials, multiplexing and machine learning (for complex detection scenarios), portability and removal of the need for pre-treatment	González-Hernández et al. (2022a), Geng et al. (2022), Chen et al. (2021b), Atta and Tuan (2023), Chio et al. (2021), Hong et al. (2022), Mao et al. (2021d), Wang et al. (2022f), Ye et al. (2023), Alder et al. (2021), Fang et al. (2020), Liyanage et al. (2020), Mao et al. (2021d), Picone et al. (2020), Smith et al. (2021)	Mao et al. (2021c)
	Hallucinogen	Looks at the detection of drugs in urine through the rapid formation of Ag nanoclusters	Chen et al. (2021b)	
	Pharmaceuticals	Papers look at the need for SERS techniques capable of handling real-world samples and the use of nanomaterials (MOF-gold core-satellite nanostructure and Au-coated Si nano-cone) for label-free, portable SERS	Boroujerdi et al. (2022b), Fan et al. (2022), Ren et al. (2022)	
	Dissociatives	Papers look at π-metal interaction for co-assembly enabling ultratrace detection and identification of a novel norketamine precursor	Ding et al. (2023), Yen et al. (2022c)	
	Cannabinoids	Looks at the detection of drugs in urine through the rapid formation of Ag nanoclusters	Chen et al. (2021b)	
	Opioids	Key themes were nanomaterials, interferents (complex samples), quantitative detection and portability	Ding et al. (2023), Li et al. (2023c), Su et al. (2023), Zhang et al. (2021d), Zhao et al. (2022d), Li et al. (2020), Liyanage et al. (2020), Akçan et al. (2020), Fedick et al. (2020), Han et al. (2021), Ye et al. (2021)	
	Other	Key themes were nanomaterials, portability, dual detection and eco-friendly materials	Gozdzialski et al. (2023), Cai et al. (2022a), Atta and Vo-Dinh (2023), Azimi and Docoslis (2022), Zhang et al. (2022g), Goel et al. (2022), Zhang et al. (2021e), Wille and Elliott (2021), Vunckx et al. (2020), Wu et al. (2020d), Burr et al. (2020), Han et al. (2020), Zhang et al. (2020g), Zhu et al. (2020)	Hang et al. (2022)
*Other*	Depressants	Papers look at near IR for differentiation of new psychoactive substances and a review of ethanol intoxication sensing technologies is presented. A systematic review for drug-facilitated sexual assault monitoring is also presented	Kranenburg et al. (2022a)	Paprocki et al. (2022), Soni et al. (2021)
	Stimulants	Papers look at a variety of detection technologies including surface plasmon resonance and focus on extraction in complex media	Kranenburg et al. (2022a), Far et al. (2022), Qiu et al. (2023), Yao et al. (2022b), Özgür et al. (2020)	
	Hallucinogen	Papers look at near IR and the influence of water of crystallisation	Kranenburg et al. (2022a), Kranenburg et al. (2023)	
	Pharmaceuticals	Papers look at asthma inhaler use via terahertz spectroscopy and a β-cyclodextrin holographic sensor for ibuprofen detection	Tyree et al. (2023), Yu et al. (2023c)	
	Dissociatives	A systematic review for drug-facilitated sexual assault monitoring is presented		Soni et al. (2021)
	Cannabinoids	Paper looks at near IR for differentiation of new psychoactive substances	Kranenburg et al. (2022a)	
	Opioids	Paper looks at polyanionoic cylcodextrins in the detection of fentanyl	Mayer et al. (2023)	
	Other	Key themes were nanomaterials, portability and interferents	Dahiya et al. (2022), Díez-Pascual et al. (2022), Alonzo et al. (2022), Kranenburg et al. (2022b), Kranenburg et al. (2022c), Rui et al. (2023), Hung et al. (2022), Rahman et al. (2020)	Pena-Pereira et al. (2021), Senesi et al. (2021), Moradi et al. (2022), Yeasmin et al. (2022), Zubrycka et al. (2022)
Currently used by UK police and authorities				
Current on-site testing for powdered, liquid or pill samples use NIK tests (ProPublica, 2025; Symonsbergen et al., 2018). For analysis in saliva and sweat the UK police use DrugWipe (lateral flow immunoassay detection) (DrugWipe, 2025)				

##### 3.2.10.1 Portability, Affordability and ease of use

Key to successful implementation of roadside drug testing is portability, with many review papers (Teymourian et al., 2020; Ren et al., 2021; Moradi et al., 2022) suggesting further work that needs to be done to achieve this.

SERS technology on a paper-based substrate enables on-site detection, such as for fentanyl citrate in serum and urine. This method utilises a paper-based SERS substrate embedded with chloride ion treated gold nanospheres, with SERS spectra collected using a portable Raman spectrometer (Han et al., 2021).

The development of a fluorescence immunochromatographic assay (FICA) strip reader provides low cost, user-friendly, highly sensitive and rapid detection capabilities, enabling convenient on-site testing (Wu et al., 2021a). This sensor incorporates a photoelectric adjustment system, leveraging the linear correlation between fluorescence and excitation light intensity, enabling precise tuning of the excitation light intensity. Such adjustment broadens the potential detection range for target analytes. This technology will have implications in many sensing devices for various target analytes on-site.

Truta et al. (2020) show that electrochemical methods can rapidly determine drugs with rapid, sensitive, selective detection in complex human matrices (e.g., blood, urine or saliva) and are easily miniaturised for on-site use. However, the electrochemical sensing of illicit drugs so far has been limited to academic research–no commercial market appears to have been conquered yet. Square-wave voltammetry (SWV) is the most widely used voltammetry technology for facile and rapid quantitative sensing of illicit drugs (De Rycke et al., 2020). The choice of electrode, especially the working electrode, can have a large impact on a sensor’s sensitivity. De Rycke et al. (2020) predict that carbon paste electrodes will further gain popularity and be used in most electrochemical sensors for the detection of illicit drugs as they are flexible in design with the possibility for miniaturisation. The presence on the market of cheap and disposable electrochemical cells, namely, screen-printed electrodes, has made feasible the creation of effective devices for the quantification of illicit drugs in an on-site screening test (Zanfrognini et al., 2020).

Nanomaterials, with their unique properties and low cost, enhance electrochemical sensor sensitivity for detecting drugs in low concentrations (Truta et al., 2020). However, future electrochemical sensor development needs to consider the implementation of biocompatible and environmentally friendly materials (Klimuntowski et al., 2020). Many articles discuss the potential of paper-based portable sensors to aid police enforcement (Noviana et al., 2019; Sha et al., 2020; Han et al., 2021; Ameku et al., 2021; Rocha et al., 2021a; Ribeiro et al., 2020; Dias et al., 2021; Saisahas et al., 2022a; Pholsiri et al., 2023; Ataide et al., 2020; Solin et al., 2023; Alder et al., 2021; Mao et al., 2021c). Cellulose is also discussed as a potential low-cost, environmentally friendly supporting material for biosensors, whose high number of hydroxyl functional groups provide the ability for the construction of novel materials for new advanced biosensor-based applications (Kamel and Khattab, 2020).

A pivotal review article surveys literature spanning the last 2 decades, focusing on optical and electrochemical sensing technologies for analysing methamphetamine (Khorablou et al., 2021). Many low-cost sensing technologies have been outlined from fluorescence to electro-chemiluminescence highlighting the range of existing and potential low-cost sensing platforms for methamphetamine. Application of these developed sensing technologies to other drug sensing provides a low-cost, high-yield route for further sensing development.

To reduce errors of inference by non-experts (i.e., most police officers do not have chemistry degrees), on-site drug tests would need to be simple to operate and interpret. Therefore, recent publications for on-site testing have focused on the simplification of current systems. Research includes the design of data processing software to simplify measurements (Noviana et al., 2019), potential for smartphone control of sensors (Madani-Nejad et al., 2023), and the development of smartphone apps for interpreting data (Garcia-Cruz et al., 2020).

##### 3.2.10.2 Matrix tolerance and interferents

Roadside drug sensors will require detection in a human matrix which introduces issues of interference and may require sample pre-treatment (Zanfrognini et al., 2020). Therefore, testing in a greater range of potential matrices to make technologies field ready is attractive (Papadopoulos et al., 2020). Electro-chemiluminescent screening using a Nafion film on a glassy carbon electrode is a sensing technique that requires little to no extraction or sample preparation. This provides ideal implementation for on-site screening in serum, urine and saliva (Dokuzparmak et al., 2021). Saliva-based drug detection is of particular interest for on-site screening, as unlike blood assays, it does not require invasive sample collection (Truta et al., 2020). Selective discrimination of illicit drugs and their metabolites is a key theme to be explored for many sensing devices (Gill et al., 2020).

##### 3.2.10.3 Specificity

For roadside drug testing, high specificity is needed to ensure the fair apprehension of suspects. Many drugs, due to degradation (Truta et al., 2020), are identified through their metabolites in body fluids (Wille and Elliott, 2021). Technologies outlined to improve specificity include the use of novel recognition elements such as aptamers (Ahmed et al., 2020) – which provide a cheap method to bind to target groups enabling sensing–and the combination of two sensor elements. For example, molecularly imprinted polymers and graphene quantum dots as a signal amplifier offer a revolution in sensor design by increasing surface area and conductivity (Khorablou et al., 2021).

##### 3.2.10.4 Multiplexing

Multiplex detection is increasingly important, allowing simultaneous analysis of multiple analytes (Mani et al., 2021; De Rycke et al., 2020; Klimuntowski et al., 2020). This will be vital for on-site drug tests as there are many potential target drugs that need to be identified. Paper-based sensors have the potential for multiplexed detection and will be important for on-site application (Noviana et al., 2019). Lateral flow tests provide rapidity, simplicity, relative cost-effectiveness, and the possibility to be used by non-skilled personnel. However, drawbacks include possible cross-reactivity, matrix interference and (easy) manipulation by users (e.g., the use of soda to cause a false positive). A lateral flow immunoassay combines multiple lines to increase detection capability, where each line contains a specific recognition element for different target analytes. However, the addition of multiple recognition sites and therefore lateral flow lines requires an increased sample volume, higher fabrication costs and increased reagent use (Nardo et al., 2021).

The importance of illicit drug analysis in combating crime is evidenced by the volume of publications identified and the variety of sensing technologies used. From the above analysis it is evident that portability, specificity, matrix tolerance and multiplexing are key components that require further research for a commercial on-site drug test. It is observed that low-cost, simply operated, portable devices are being developed, including paper-based devices requiring little to no extraction and sample preparation. Advances are also being made to increase detection limits and reduce costs, with a focus on saliva-based detection. However, more research is needed particularly in the discrimination between certain target analytes including amphetamine type stimulants. This is particularly critical as often in illicit drug analysis the target is unknown and therefore determination of its exact identity is important. Portable sensor development should involve environmentally friendly materials and the use of nanomaterials alongside the development of software for the extraction of data. The specificity of the sensor will be vital and an understanding of drug concentrations, half-life and metabolites in the matrix will also be key. Electrochemical sensing, particularly SWV, has yet to impact the commercial market for mass on-site detection but its relative cheapness, ease of use and sensitivity make it ideal.

With the focus on low cost testing we are looking at detecting drug offenses on-site. For example, identifying those under the influence with drug driving, drink spiking and identifying samples rapidly. Specific drug detection kits used by the UK police for on-site analysis vary depending on factors such as region, budget of the police force and advancements of technology (Evidential Drug Identification Testing, 2023).

UK police use various kits for on-site drug analysis, such as NIK tests for powders, pills, or liquids (Symonsbergen et al., 2018). These reagent tests including Marquis (MDMA), Mandelin (MDMA, opiates), Scott (cocaine), and Simons (MDMA, methamphetamine) utilize colorimetric methods and are popular for their ease of use and quick results. However, they have limitations. Their sensitivity and specificity are restricted, leading to potential false positives or negatives due to interference from other substances. This means they may miss low concentrations of drugs or misidentify substances, necessitating confirmatory testing for accurate results (ProPublica, 2025). Additionally, these tests typically only cover a limited range of drugs, potentially missing emerging substances. This necessitates the use of multiple tests or techniques to accurately identify the presence and type of drugs in a sample, which can be time-consuming and resource-intensive for law enforcement agencies.

For drug driving analysis in saliva and sweat, the UK police currently use the Securetech & Dreiger (DrugWipe (DrugWipe, 2025)) sensor to detect cannabis and cocaine. DrugWipe employs a lateral flow immunoassay detection system based on the principle of competitive binding. An anti-drug antibody binds either to a protein conjugate line, forming a visible test line, or to the target drug, if present, reducing the appearance of the test line (DrugWipe, 2025). This technology has type approval (Testler, 2025), indicating it meets the specifications required by the Home Office. However, having roadside evidential screening that allows the analysis to be used as court evidence would be beneficial for the police.

Therefore, current on-site drug detection kits provide only preliminary results, requiring confirmatory testing by certified laboratories - often using high-performance liquid chromatography-mass spectrometry (HPLC-MS) - for legal purposes, leading to additional costs and delays in proceedings (ProPublica, 2025).

Recent research highlighted in the SR indicates promising advancements to address current challenges. The need to detect a broader range of drugs is addressed with research into dual detection methods, such as FICA strip readers, enabling the detection of multiple drugs simultaneously (Wu et al., 2021a). Lateral flow immunoassays are being investigated for multiplexing capabilities, although further research is required to reduce matrix interference (Nardo et al., 2021). Enhancements in specificity are being explored through the use of aptamers, MIPs and quantum dots (Khorablou et al., 2021; Ahmed et al., 2020). To overcome current issues related to matrix tolerance, technologies such as electro-chemiluminescent screening are also being researched (Dokuzparmak et al., 2021).

#### 3.2.11 Other

Only 35 (5%) publications meeting the inclusion criteria did not focus on specific target analytes, affirming the appropriateness of chosen key analyte categories. These publications included a fairly even split of the sensing technology categories (Tables 9, 10).

**TABLE 10:** Summary of included publications relating to the use of sensors in target analytes not covered by the outlined categories.

Sensing technology	Description	Research papers	Review papers
*Colourimetric*	Papers look at eco-friendly materials with paper-based microfluidic devices and a focus on nanomaterials	Musile et al. (2021), Upadhyay et al. (2022), Dang et al. (2022)	Costanzo et al. (2023)
*Electrochemical*	Key themes were nanomaterials monolayer-protected gold nanoparticles, 2D materials, graphite electrodes, 3D electrodes, and wireless wearable electrochemical sensors and eco-friendly materials low-cost stencil printing of graphite electrodes	Kajale et al. (2021), Upadhyay et al. (2022), Dang et al. (2022), Kongkaew et al. (2022)	Abdelkader et al. (2022), Abdulhussein et al. (2022), Alves et al. (2021)
*Immunoassay*	Papers look at pH-switchable nanozymes and paper-based devices	Musile et al. (2021), Liang et al. (2023)	Alberti et al. (2023)
*Luminescence*	Key themes were multiplexing, portability and *ad hoc* reviews looking at fluorescent chemical sensors, sulfonamides and nanomaterials	Shah et al. (2023), Musile et al. (2021), Upadhyay et al. (2022), Zhang et al. (2022h), Calabretta et al. (2021), Zhao et al. (2021), Gasser et al. (2022)	Costanzo et al. (2023), Fakayode et al. (2023), Batool et al. (2022b)
*Raman*	Key themes were portability, novel substrates and *ad hoc* reviews saliva studies and progress in SERS scattering molecular sensing	Guo et al. (2022b), Pliatsikas et al. (2021), Parungao et al. (2022), Aleknavicene et al. (2022), Fularz et al. (2021), Khinevich et al. (2021), Lin et al. (2023b), Zhang et al. (2022b), Zhao et al. (2021)	Hardy et al. (2022), Mandal and Tewari (2022)
*Other*	Papers look at smartphone-based sensors, carbon dots and microfluidic designs	Kim et al. (2022), Ross et al. (2023), Khayal et al. (2021), Liu and Niu (2022), Marques et al. (2023)	Kulkarni et al. (2022), Geballa-Koukoula et al. (2023)

Many publications focussed on the detection of radioactive materials (Marques et al., 2023; Kim et al., 2022). Studies highlighted the advantages of silicon photomultipliers in beta and gamma detectors over current radiation monitors used at seaports, citing their lightweight, compact design, and lower power consumption (Marques et al., 2023). Emphasis was placed on developing environmentally friendly alternatives to current sensing technologies, including sustainable printed electrochemical platforms (Kongkaew et al., 2022) and paper-based microfluidic devices (Musile et al., 2021). Portability emerged as a key theme, with innovations such as a mobile fibre-optics Raman spectrometer addressing challenges of dispersive Raman spectroscopy and potentially enabling mobile spectroscopy applications, such as for elephant ivory (Parungao et al., 2022). Additionally, several *ad hoc* reviews were identified (Costanzo et al., 2023; Abdelkader et al., 2022; Abdulhussein et al., 2022; Alves et al., 2021; Alberti et al., 2023; Fakayode et al., 2023; Batool et al., 2022b; Hardy et al., 2022; Mandal and Tewari, 2022; Kulkarni et al., 2022; Geballa-Koukoula et al., 2023), covering diverse topics such as 3D electrodes in electrochemical sensing (Abdelkader et al., 2022), SERS studies on saliva (Hardy et al., 2022) and advances in SERS for molecular sensing (Mandal and Tewari, 2022).

#### 3.2.12 UK crime statistics (2022)

Combining our systematic review findings with knowledge of current sensing devices and their limitations, along with an understanding of the volume and economic impact of various crime types, enables the identification of critical gaps where further research could significantly enhance crime prevention and response efforts.

In the UK during 2022, there were 498,381 reports of criminal damage and arson, with arson estimated at 10%–15% of cases, translating to 53,000 to 79,500 arson incidents (Criminal Damage and Arson Crime and Safety Statistics, 2025; Official Statistics, 2025). Firearms offenses totalled 5,850 (CENSUS, 2023), while counterfeit currency offenses totalled 5,600 (Ministry of Justice, 2023). Waste crime, prosecuted by the Environment Agency, costs the economy approximately £1 billion annually, leading to nearly 100 prosecutions (Environmentagency, 2023). DNA profiling aided in solving 22,477 cases, including 550 rapes and 644 homicides (Corporate Report, 2023). Explosives-related offenses totalled 348 (Corporate Report, 2023), and poisonings with intent to harm numbered 105 (Office for National Statistics, 2022). Fingerprint evidence led to 22,000 matches, solving 8,472 burglaries, 3,409 vehicle crimes, and 1,529 instances of criminal damage (Corporate Report, 2022). 610 reported food safety cases were potentially up to 3,050 due to underreporting, with a financial impact of up to £1.96 billion (Food Standards Agency, 2025b; Food Standards Agency, 2025c; Food Safety News, 2025; Food Standards Agency, 2025a). Drug-related offenses reached 200,000, with a 21% increase in drug seizures compared to 2021 (Official Statistics, 2022). In 2022, UK roadside drug wipes increased to 6,273 from 4,668 in 2021, with 53.6% testing positive (NPCC, 2025), while drink/drug driving convictions rose 40% from 2014, making drug driving a growing concern (Drug Testing Clinics, 2025).

These statistics underscore the varied impacts of different crimes, guiding the focus of future research efforts. In the UK, gunshot and counterfeit crimes had minimal impact, consistent with the low number of related SR publications. Conversely, explosives cases were fewer than expected from the SR, but their economic and social impact can be severe, justifying further research. Poisoning cases were also surprisingly low, possibly due to the narrow focus of available crime statistics indicating a need for more comprehensive data on poisons and toxins.

Pollutant and food safety crimes, though reported less frequently, have a substantial economic impact, advocating for increased research attention. Comprehensive crime statistics on body fluids remain elusive, but DNA profiling highlights numerous severe cases such as homicides and rapes, showing significant potential for combating crime. Despite this, the low number of included publications in the SR suggests insufficient research in this area. It is unclear if this is due to sufficient existing sensing devices or a research gap needing exploration, warranting further investigation.

Fire-related crimes are numerous but underrepresented in included SR publications, suggesting current technologies may suffice. Fingerprint analysis accounts for a significant number of crimes and 14% of SR publications, justifying continued research efforts.

Drug-related crimes, comprising 36% of included SR publications, have seen a dramatic increase, particularly in drug driving offenses. The substantial volume of research and crime statistics in this area indicates a significant impact and underscores the need for continued and enhanced research efforts to address drug-related crime effectively.

## 4 Conclusion

This report offers a concise analysis of recent advancements in low-cost sensing technologies for crime reduction, based on a systematic review. Analysis of publication trends indicates substantial growth in research focused on sensors for combating crime (125% increase in included publication numbers from 2017 to 2022). Taking stock of the literature, as was done here using a systematic approach, is thus important to identify trends and research gaps that should be pursued.

Included publications were categorised into the analytes that they targeted (illicit drugs, fingerprints, explosives, body fluids, food safety, poisons and toxins, pollutants, counterfeits and documentation, fire, gunshot and other) and the types of sensing technology used (high cost: mass spectrometry, PCR, HPLC and low cost: electrochemical, colourimetric, immunoassay, luminescence and SERS). The proportion of publications from each target analyte category remained fairly constant over the period reviewed. However, an increase was seen in the dominance of the field by the four main categories (illicit drugs, food safety, fingerprints and poisons and toxins) and an increase in particular in the dominance of illicit drug publications. In fact, more than one-third (36%) of all publications related to the analysis of illicit drugs, of which 30% focussed on stimulant abuse.

Particular attention was given to articles published from 2020 onwards, reflecting the rapid technological advancements in this area, and to articles focussing on low-cost technologies, which are argued to be most impactful in crime reduction efforts. This further detailed analysis (782 documents) revealed trends in specific areas such as illicit drug detection, where stimulants and opioids were prominent subjects. Technologies like electrochemical and luminescence sensors showed promise for creating accessible, user-friendly testing devices.

The current challenges associated with the sensing devices used by UK police and authorities were reviewed, offering insights into how ongoing research identified through the SR may address these issues. A summary of key findings follows.

Fire Analysis: UK police and fire services collaborate using tools such as gas chromatography-mass spectrometry (GC-MS), fire investigation dogs, and thermal imaging cameras. Issues include the need for portable GC-MS and the limitations of canine detection. Research into portable MOx sensors shows promise for rapid on-site analysis.

Gunshot Residue (GSR) Detection: Current methods, like colourimetric spot tests, face issues of low specificity and sample degradation. Field-deployable scanning electron microscopes (SEM) and electrochemical methods under development show potential for improved specificity and cost-effectiveness.

Counterfeit Detection: Techniques like UV and infrared light, magnetic ink detectors, and portable spectrometers are used to identify counterfeit items. However, false positives and sophisticated counterfeiting techniques pose challenges. Advances in colourimetric and luminescent sensing and magnetic nanoclusters offer improved detection capabilities.

Pollutant Measurement: Instruments like GC-MS, Raman spectrometers, photoionization detectors (PIDs), and x-ray fluorescence analysers (XRF) are used to detect pollutants. Issues include high costs, maintenance, and limited detection capabilities. Research into economical materials and dual detection methods shows promise for enhanced analyte detection.

Body Fluid Identification: Sensors such as luminol, Rapid Stain Identification (RSID) tests, and portable DNA analysers are used to detect body fluids at crime scenes. Challenges include false positives, DNA degradation, and high costs. Research into eco-friendly materials and dual detection techniques offers potential solutions.

Explosive Detection: Portable detectors, canine units, and colourimetric kits are used for on-site explosive detection. Current challenges include high costs, false positives, and limited detectable compounds. Research into dual detection methods and 3D printed electrodes for electrochemical sensors aims to address these issues.

Poison and Toxin Identification: Technologies such as portable spectrometers and biosensors are used for on-site detection. Issues include high costs, environmental stability, and limited detection range. Advances in AIE probes and paper-based methods show potential for cost-effective and comprehensive detection.

Fingerprint Detection: MobileID devices and various powders are used for on-site fingerprint detection. Current limitations include sensitivity to environmental conditions and the need for extensive training. Research into nanomaterials offers enhanced sensitivity and selectivity for fingerprint detection.

Food Safety Analysis: Local authorities use portable spectrometers, rapid testing kits, chemical test strips, and UV lights for food safety. Challenges include high costs, specificity issues, and the need for special sample preparation. Research into dual detectors and printed electrochemical devices aims to improve cost-effectiveness and sensitivity.

Illicit Drug Detection: UK police use NIK tests and DrugWipe sensors for on-site drug detection. Current limitations include sensitivity, specificity, and the need for confirmatory testing. Advances in dual detection methods, lateral flow immunoassays, and electro-chemiluminescent screening are being explored to enhance specificity and matrix tolerance.

An analysis of the prevalence of different crime types and their social and economic impacts identified research gaps that could significantly enhance crime prevention. Gunshot, counterfeit, explosives and poisoning analyses were found to have a minimal impact on UK crime compared to other analytes. While improvements in sensing for these areas would be beneficial, they should not be the primary focus in the UK at present. Pollutant and food safety analytes, though less frequently reported in crime statistics, have a substantial economic impact, indicating significant benefits from further research. The number of crimes involving body fluid analysis, particularly DNA profiling, is substantial. However, the relatively low number of related SR publications included indicates either a research gap or the adequacy of existing sensing devices. The review of SR publications relating to body fluids reveals many areas where further research is and would be beneficial, underscoring that there are significant research gaps that need to be addressed. Illicit drug-related crimes, especially drug driving, have significantly increased in recent years, comprising 36% of included SR publications. This highlights the urgent need for continued and enhanced research efforts to address these issues effectively.

Overall, the review highlights significant progress in low-cost sensing technologies for crime reduction, addressing key challenges and proposing innovative solutions for more efficient and effective crime detection and analysis. These advancements indicate promising pathways for enhancing crime detection and public safety through accessible, reliable sensing technologies. Future efforts should focus on refining dual detection methods, reducing matrix interference, and fostering collaboration between academia and law enforcement for effective implementation.

This systematic review of sensing technologies to combat crime is intended to provide policymakers, law enforcement agencies, and researchers with a comprehensive and timely evaluation of existing research, guiding strategic decisions on technology adoption and resource allocation. By identifying gaps and future research directions, the review is also intended to stimulate innovation and development of advanced sensing tools, crucial for combating sophisticated criminal activities.

## 5 Future outlooks

### 5.1 Study limitations

This work has provided a systematic review and detailed understanding of the existing sensing technologies for combating crime and key gaps in the literature where further work would be beneficial.

#### 5.1.1 Systematic review

Although a systematic approach was taken here to ensure repeatability and extensive coverage, there is a possibility that the search terms used, or the application of the inclusion/exclusion criteria may have resulted in relevant studies being missed or excluded. Moreover, journal space limitations preclude the discussion of all insights extracted from the included studies. For example, a summary of the methods used in every publication for each analyte category would provide more understanding of the existing literature but would increase the length of the review significantly.

#### 5.1.2 Other languages and countries

This review only included publications written in English, which means studies from non-English-speaking countries may have been excluded. This limitation highlights the potential loss of key publications and suggests that the findings of this systematic review might not be universally applicable. Engaging with international stakeholders and experts could help address this limitation and ensure a more comprehensive understanding.

### 5.2 Future directions

#### 5.2.1 Patent search

Alongside a search of academic databases, it would be beneficial to search recent patent applications and grants relating to sensors utilised for combating crime. The same key search queries can be used to search databases such as Google Patents and Derwent Innovations. This will outline the most recent sensing technologies that are currently being developed and may indicate further key gaps and avenues for future beneficial research.

#### 5.2.2 Low hanging fruit

Low hanging fruit arguably represent the best directions for further research that will allow the rapid and easy development of mass producible sensors to combat crime. To expedite advancements in sensing technologies for crime detection, focusing on integrating AI and IoT for enhanced data analysis and connectivity is paramount. Concurrently, investing in advanced materials like nanomaterials and conductive polymers can significantly improve sensor performance while reducing production costs and environmental impact. Standardising portable designs and enabling multiplexed detection capabilities are also crucial for practical deployment. Lastly, fostering collaborations between stakeholders, law enforcement officials, and government science technology agencies will accelerate the translation of research innovations into commercial products, ensuring robustness, scalability, and regulatory compliance of next-generation sensing devices.

#### 5.2.3 Manufacture of a sensor

The overarching aim in creating this SR was to, alongside an understanding of current developments, understand gaps in the literature for further research. Further research in these areas should ultimately lead to the creation of a low-cost, portable sensing device that can be used for its chosen analyte to combat crime by its mass manufacture and deployment. Considerations of long-term storage, matrix tolerance and approval from law enforcement agencies will be vital.

#### 5.2.4 Global megatrend

A global megatrend is a large-scale, sustained shift in major social, economic, environmental, technological, or geopolitical patterns that significantly transforms multiple industries and aspects of life over decades (PwC, 2025; META, 2016). This therefore makes it an important consideration when looking at further research into sensors to combat crime.

The global megatrend for sensors to combat crime encompass advancements in AI, IoT, and big data, which are enhancing the capabilities and integration of these technologies. Urbanization and connectivity improvements, particularly through 5G and cloud computing, are driving the adoption and effectiveness of sensors to combat crime. Economic, social, and environmental considerations further shape the development and deployment of these systems, highlighting the need for cost-effective, ethical, and resilient solutions in combating crime.

#### 5.2.5 Sustainable development goals

The Sustainable Development Goals are pivotal in addressing global challenges by promoting equity and sustainability through interconnected goals and specific 2030 targets, thereby fostering international cooperation and accountability (United Nations, 2025). Therefore, considering these goals is essential when exploring further research into sensing devices to combat crime.

Research in this field not only drives technological advancements (Goal 9) but also contributes to achieving societal goals such as safety, justice, and sustainable urban development (Goals 11 and 16). Furthermore, ensuring the ethical deployment and use of sensor technologies aligns with the overarching principles of sustainable development, emphasizing inclusivity, safety, and justice for all (Goal 17).

#### 5.2.6 Emerging technologies

Rapid advancements in sensor technology mean that newer studies may use more advanced sensors not covered in older reviews. Keeping reviews up-to-date with the latest technologies and applications is challenging but crucial. Whilst this SR aimed to provide a comprehensive overview of sensing technologies for combating crime, future reviews may benefit from focusing separately on specific target analytes to achieve a more nuanced understanding and detailed analysis.

## Footnotes

1 TargetZero is an initiative to reduce the number of traffic deaths and serious injuries to zero by 2030.
