# Supplementary material for: A systematic review of sensors to combat crime and routes to further sensor development
Source: Front Chem. 2025 Jun 12;13:1568867. doi: 10.3389/fchem.2025.1568867 (PMC12198248; doi:10.3389/fchem.2025.1568867)
Supplement: Supplementary file 1 [file Supplementaryfile1.docx]

Supplementary Material

In the systematic review, a representative selection of papers for each target analyte was briefly presented and discussed. This aimed to highlight key points and provide a broad overview, enabling the reader to appreciate the current state of the literature, identify existing gaps, and understand potential future directions. For completeness, this supplementary information offers a more in-depth examination of the selected studies. It focuses on the sensing mechanisms employed, along with their respective advantages and disadvantages, to give the more engaged reader a deeper understanding of the current research landscape beyond the scope of the main text.

# Fire

Table 1 Overview of selected fire sensing study, highlighting the sensing mechanisms employed along with their respective advantages and disadvantages.

| **Ref.** | **Title** | **Discussion** |
| --- | --- | --- |
| (1) | *Expanding Horizons of Metal Oxide-based Chemical and Electrochemical Sensors* | The identified publication reviewed metal oxide (MOx) electrochemical sensors for hazard-surveillance and risk investigation, including fire-hazards, chemical-warfare agents, oil-spills and explosives. MOx sensors, combined with airflow detectors and specific algorithms, have been used in mobile robots for fire analysis. These developments are made possible by novel nanoarchitectural patterns which enhance sensitivity and the possibility of multi-analyte sensing using array sensors and blended composites.  SENSING MECHANISMS:  MOx sensors are based on changes in electrical resistance caused by interactions between the sensor surface and target gas molecules. Oxygen species adsorbed on the MOx surface withdraw electrons, altering the charge carrier concentration, and these resistance changes are influenced by factors such as grain size, morphology, doping, and environmental conditions. 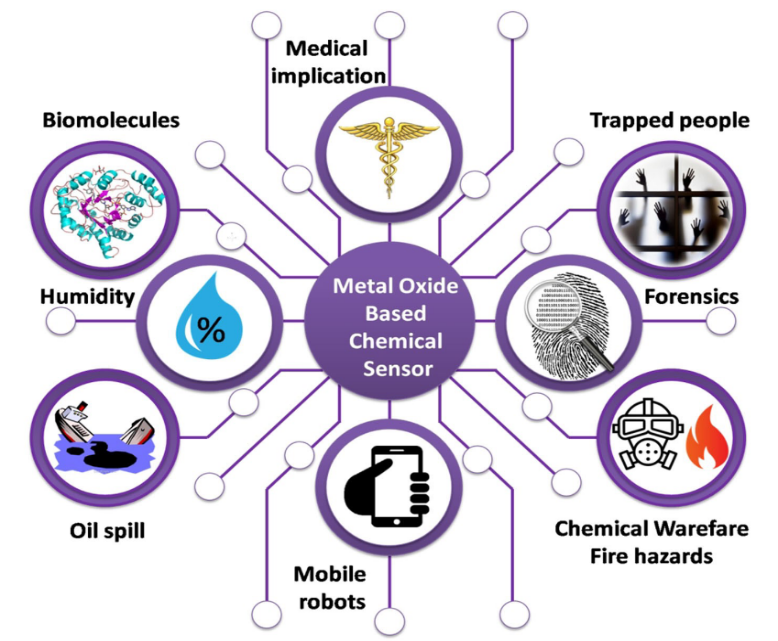 Figure 1 Emerging metal oxide chemical sensor applications  Reviewed MOx semiconductor sensors include:   - **Tin oxide (SnO_2_)** – thin films (via spray pyrolysis), nanowires and nanocolumns and hollow nanofibers doped with other MOx were highlighted for their ability to detect combustion products efficiently and provide improved sensitivity and lower detection temperature utilising heterojunction formation. - **Tungsten oxide (WO_3_)** – responds well to smoke gases - **Nickel oxide (NiO)** – shows high sensor response but may negatively affect sensor performance when used in composites. - **Indium Oxide (In_2_O_3_)** – was used to enhance sensor performance in combination with SnO_2_.   The authors noted that improving thermal damage prevention, response, recovery times, and robot mobility is necessary to realize the potential of these devices. |

# Gunshot

Table 2 Overview of selected gunshot sensing studies, highlighting the sensing mechanisms employed along with their respective advantages and disadvantages.

| **Theme** | **Ref.** | **Title** | **Discussion** |
| --- | --- | --- | --- |
| Electrochemical | (2) | *Simultaneous determination of lead and antimony in gunshot residue using a 3D-printed platform working as sampler and sensor* | *Castro et al.* detail the use of 3D-printed electrodes for simultaneous and semi-quantitative detection of lead and antimony (both present in GSR) without the need for sample preparation. 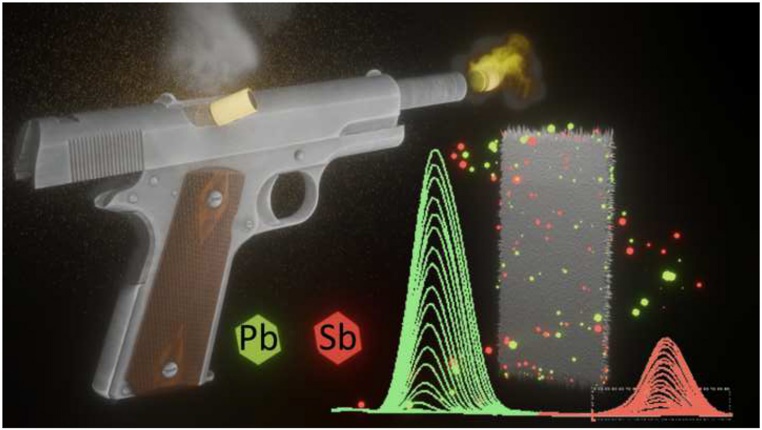 Figure 2 Graphical abstract depicting the electrochemical peaks for lead and antimony (at varying concentrations) observed upon analysis.  SENSING MECHANISMS:  Lead and antimony metals ions are detected here using anodic stripping voltammetry, this involves the pre-concentration of metal ions on an electrode surface. The stripping phase then uses an electrode potential swept in the positive direction, causing the deposited metals to oxidize and release electrons, generating current peaks that are directly proportional to the concentration of each metal in the sample.  Simultaneous detection reduces the numbers of tests needed to ensure identification of different types of GSR, reducing costs and time. Furthermore, the augmentation of electrode response through doping is shown to enable detection at lower concentrations |
|  | (3) | *Nitrite amperometric sensor for gunshot residue screening* | *Promsuwan et al.* demonstrated enhanced electrocatalytic response with palladium doped glassy carbon microspheres.  SENSING MECHANISMS:  The electrochemical mechanism used is amperometry, where the sensor measures the current generated by the electrochemical oxidation of nitrite, a key marker found in gunshot residue. Thereby enabling quantitative detection, as the current is directly proportional to the nitrite concentration. 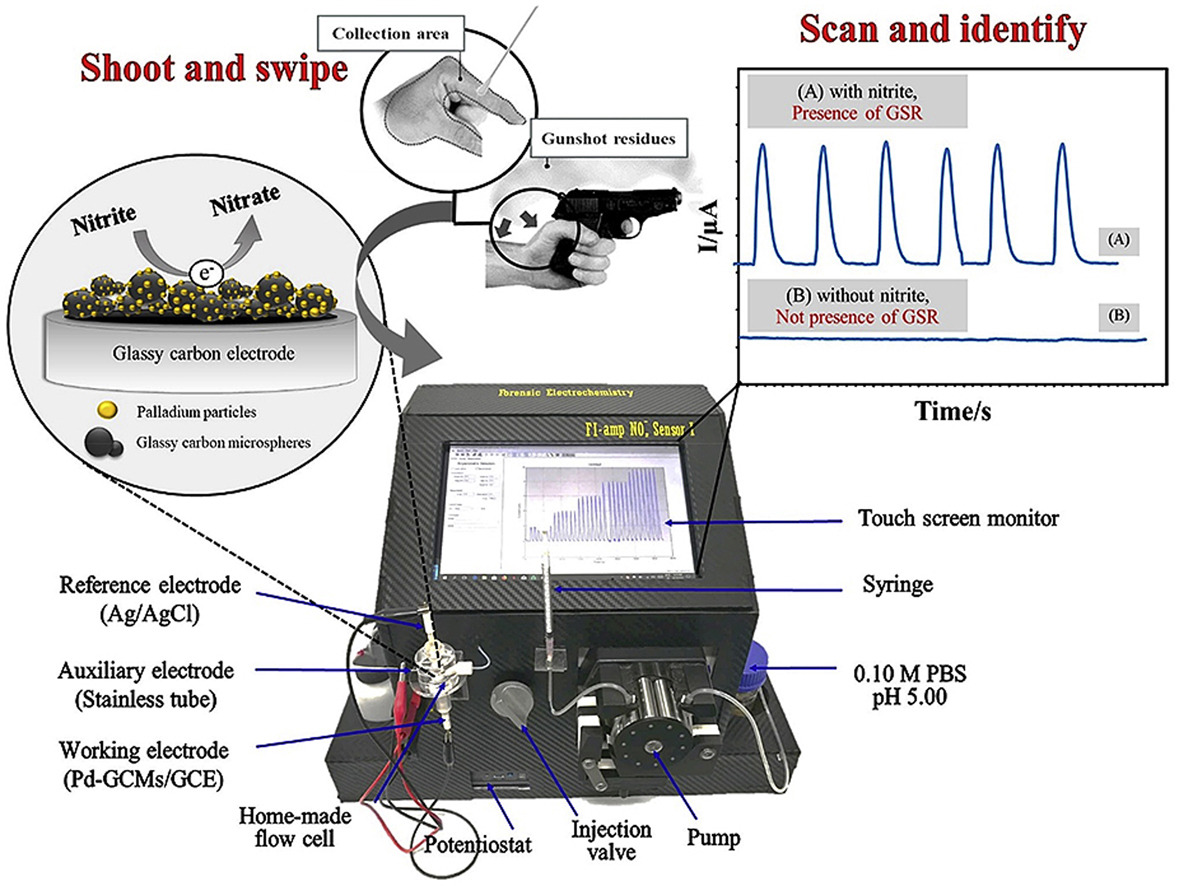 Figure 3 Graphical abstract of sensing mechanisms and analysis  This nitrite sensor offers high sensitivity and fast response, with a simple, low-cost setup enabling on-site screening. The selective detection of nitrite enables detection without sample pre-treatment. However, potential issues of interference from other electroactive species, sensor degradation over time and its restriction to nitrite detection which may result in an incomplete gunshot residue profile. |
|  | (4) | *Magnetic nanoparticle modified electrodes for voltammetric determination of propellant stabiliser diphenylamine* | *McKeever et al.* used voltametric electrodes with magnetic nanoparticles for propellant stabilizer detection (diphenylamine).  SENSING MECHANISM:  Voltametric sensing is detailed using electrodes modified with magnetic nanoparticles to enhance sensitivity. Diphenylamine undergoes electrochemical oxidation at the electrode surface, generating a current signal that correlates with its concentration, while the magnetic nanoparticles increase the electroactive surface area and improve electron transfer kinetics, enabling more efficient and sensitive detection. 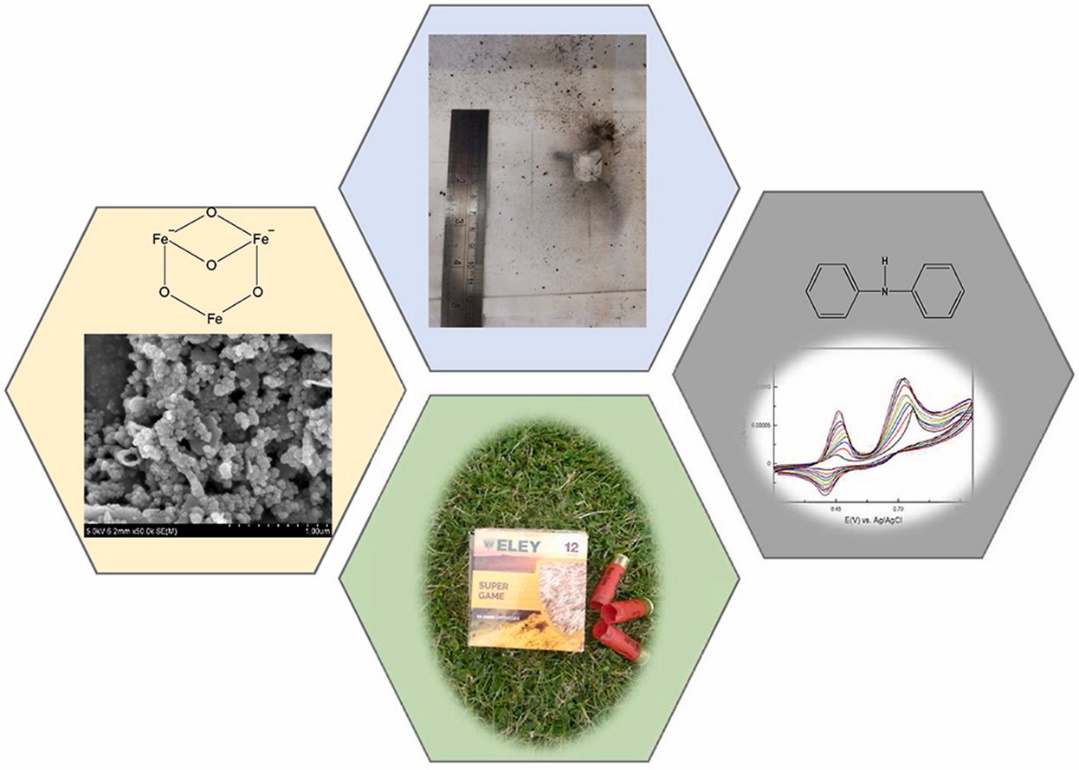 Figure 4 Graphical abstract detailing the sample types and voltammetric analysis.  The use of magnetic nanoparticle-modified electrodes offers advantages such as enhanced sensitivity, improved electron transfer, and easy manipulation of the electrode surface, making them well-suited for detecting low concentrations of diphenylamine in complex samples. However, drawbacks include potential nanoparticle aggregation, reduced long-term stability and reproducibility, increased fabrication complexity, and possible interference from other electroactive species. |
|  | (5) | *Gunshot residue detection technologies—a review* | This is an ad hoc review highlighting the benefits of advanced electrochemical methods due to their user-friendliness, sensitivity, and cost-effectiveness. 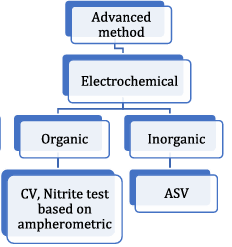 Figure 5 Flowchart representing various electrochemical methods developed to detect GSR. ASV (abrasive stripping voltammetry) and CV (cyclic voltammetry).  SENSING MECHANISM:  A focus is seen on the detection of organic gunshot residue (OGSR) components through techniques such as amperometry, voltammetry, and electrochemical impedance spectroscopy. These methods rely on the redox activity of target compounds—like nitrite, diphenylamine, and other stabilizers—at the electrode surface, generating measurable electrical signals. Modifications to electrodes, such as the use of nanomaterials or molecularly imprinted polymers, enhance sensitivity, selectivity, and detection limits for trace-level analysis in complex matrices.  These electrochemical methods are noted for their high sensitivity, rapid analysis, simplicity, and cost-effectiveness. They also offer portability, making them suitable for on-site detection without the need for extensive sample preparation. One limitation is the inability to detect barium due to its high electrochemical potential, which poses challenges in its analysis using these techniques. |
|  | (6) | *Trends in Gunshot Residue Detection by Electrochemical Methods for Forensic Purpose* | This is also an ad hoc review highlighting the benefits of advanced electrochemical methods due to their user-friendliness, sensitivity, and cost-effectiveness.  SENSING MECHANISM:  Electrochemical methods for GSR detection primarily focus on analysing both inorganic and organic components of residues. Inorganic elements such as lead (Pb), antimony (Sb), and barium (Ba) are typically detected using anodic stripping voltammetry (ASV), which involves pre-concentrating the analytes onto the electrode surface and then measuring the stripping current after applying a potential sweep. Organic compounds like nitroglycerin (NG) and dinitrotoluene (DNT) are identified through techniques like cyclic voltammetry (CV) and square-wave voltammetry (SWV), which assess the redox behaviour of these substances. Recent advancements have led to the simultaneous detection of both organic and inorganic GSR components in a single analysis, enhancing the efficiency and comprehensiveness of forensic evaluations. 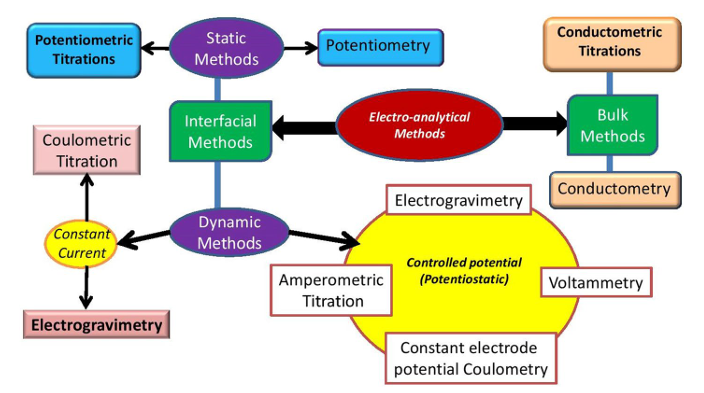 Figure 6 Classification of electroanalytical methods.  These methods for GSR detection offer advantages such as high sensitivity, portability, cost-effectiveness, and rapid analysis, making them suitable for on-site and routine forensic applications. However, they face challenges like limited detection of certain elements, potential interferences from other substances, and the need for specialized knowledge to operate and interpret the results accurately. |
|  | (7) | *Promising Applications of Additive-Manufactured (3D-printed) Electrochemical Sensors for Forensic Chemistry* | A third more general review highlighted the benefits of 3D-printing in electrochemical sensing as a powerful, affordable and accessible tool emphasising the importance of these research endeavours reaching end users.  SENSING MECHANISMS:  The sensing mechanism discussed involves using a 3D-printed G/PLA electrode to identify gunshot residue (GSR) by employing square-wave anodic stripping voltammetry (SWASV). SWASV is a highly sensitive electrochemical technique used for trace metal analysis. It involves applying a square-wave potential to the electrode, followed by a stripping step where metal ions, like Pb²⁺ and Sb³⁺, are reduced and then oxidized, generating a current proportional to their concentration.  This technique enables the simultaneous detection of lead (Pb²⁺) and antimony (Sb³⁺) ions, key components of GSR, with high sensitivity and selectivity, while addressing challenges such as the complexity of GSR samples and potential interference from other metals. |
| Luminescence | (8) | *Voltammetric analysis of luminescent markers in gunshot residues* | *Chedid et al.* discussed luminescence sensors for new nontoxic ammunition, which requires alternative detection techniques due to a lack of the conventional metals for detection of GSR.  SENSING MECHANISM:  The presence of an inorganic luminescent chemical marker in GSR is shown to be effectively detected using square-wave voltammetry on a carbon paste electrode. This luminescent marker ([(Eu₂Zr)(btc)₃(Hbtc)₀.₅]·6H₂O, where 'btc' stands for benzene-1,3,5-tricarboxylate.) typically exhibits unique electrochemical behaviour, allowing for specific identification through voltammetric scanning. The luminescent marker can be excited using voltammetric waves, and the specific electrochemical signature can be distinguished from other compounds in the sample.  The overall sensing mechanism enables the detection of very low concentrations of this marker, providing a sensitive method for GSR analysis.  The voltammetric approach is advantageous due to its high sensitivity, rapid response, and ability to distinguish specific chemical markers within complex forensic samples. Sensors able to detect these new residues will be essential moving forward and demonstrates the need for constant re-evaluation of the current target analytes and therefore techniques needed to detect them. |
| Review | (9) | *Field-portable and handheld laser-induced breakdown spectroscopy: Historical review, current status and future prospects* | *Senesi et al.* provide an ad hoc review of laser induced breakdown spectroscopy (LIBS) for gunpowder origin analysis looking at both prototype instruments and commercially available analysers.  SENSING MECHANISM:  LIBS operates by focusing a high-energy laser pulse onto a surface suspected of containing GSR particles. The laser's energy ablates the material, forming a plasma that emits light at wavelengths specific to elements like lead (Pb), antimony (Sb), and barium (Ba), which are commonly found in GSR. By analysing the emitted light, LIBS facilitates the identification and quantification of these elements, thereby confirming the presence of GSR on surfaces such as skin, clothing, or objects near a shooting incident.  Key areas for future research focus on improving the portability and analysis speed of LIBS instruments outside the laboratory while maintaining high performance. |

# Counterfeits/ Documentation

Table 3 Overview of selected counterfeits/ documentation sensing studies, highlighting the sensing mechanisms employed along with their respective advantages and disadvantages.

| **Theme** | **Ref.** | **Title** | **Discussion** |
| --- | --- | --- | --- |
| Luminescence | (10) | *The untapped potential of magnetic nanoparticles for forensic investigations: A comprehensive review* | The use of magnetic nanoclusters with super-magnetic behaviour and smaller dimensions shows significant potential for anticounterfeiting with rapid and full reversible optical responses after magnetic field application.  SENSING MECHANISM:  The sensing mechanism involves the use of magnetically tunable assemblies, where the optical properties of the MNPs, such as their colour and fluorescence, can be altered by applying an external magnetic field. This allows for the creation of dynamic, reversible colour changes and invisible inks that can be controlled for authentication purposes. Additionally, MNPs in barcode and label applications can offer multi-level security, with some systems utilizing surface-enhanced Raman scattering (SERS) or fluorescence to verify authenticity, making them difficult to replicate or tamper with.  However, the shelf-life of these nanoclusters remains uncertain, necessitating further research |
|  | (11) | *Lighting up forensic science by aggregation-induced emission: A review* | Research exploring the aggregation-induced emission (AIE) phenomenon, known for its remarkable luminescence properties, has proven successful in applications such as anticounterfeiting banknotes and confidential documents.  SENSING MECHANISM:  Aggregation-Induced Emission (AIE) molecules in anti-counterfeiting applications, highlight their ability to emit fluorescence only under specific conditions, such as UV light or near-infrared (NIR) light. These molecules, including fluorescent imidazole derivatives and NIR-transmitting diketopyrrolopyrrole materials, are used in security features like hidden codes or inks that are visible only under certain light conditions. Additionally, AIE materials with room-temperature phosphorescence (RTP) properties and reversible colour changes due to mechanical, thermal, or gas-induced stimuli further enhance security, providing dynamic and multi-level verification methods.  Advantages of AIE materials in anti-counterfeiting include their high photostability, ability to emit fluorescence under specific wavelengths (e.g., UV or NIR light), and the potential for reversible colour changes, which improve the practicality of security features. Additionally, their sensitivity to environmental factors like grinding, heating, or gas exposure provides multiple verification methods, enhancing security. Disadvantages include the need for specialized equipment (e.g., NIR cameras or UV light) for detection, and the potential for reduced effectiveness if exposed to prolonged environmental conditions, such as excessive light or wear, which could compromise their reliability in some applications. |
|  | (12) | *Carbon Dots for Forensic Applications: A Critical Review* | Carbon dots (CDs) have been discussed with this review highlighting their superior fluorescence, low-cost, non-toxic and colour-tuneable nature. For example, CDs can be incorporated into inks capable of functioning as novel barcodes and nanotags for authentication and anticounterfeit applications.  SENSING MECHANISM:  Several sensing mechanisms for anti-counterfeiting are discussed, including fluorescence, room-temperature phosphorescence (RTP), and up-conversion photoluminescence (UCPL). These mechanisms are utilized in C-dot-based systems, which allow for the creation of unique, unclonable patterns and markings that can be detected under specific light sources, such as UV or infrared light, or through changes in fluorescence lifetime or colour. Additionally, the use of materials like hydrophobic C-dots and graphene quantum dots (GQDs) enables reversible fluorescence and RTP behaviours, enhancing the complexity and security of anti-counterfeiting features.  The advantages of using C-dots and related nanomaterials for anti-counterfeiting include their ability to create unique, hard-to-duplicate patterns with multiple detection mechanisms, such as fluorescence, RTP, and UCPL, making them highly secure. They are also versatile, as they can be applied to various surfaces and materials, including banknotes, packaging, and documents. However, the disadvantages include potential challenges in mass production and scalability, as well as the need for specialized equipment to detect the security features, which may limit their accessibility for widespread use. |
| Doping | (13) | *Barium tungstate doped with terbium ion green nanophosphor: Low temperature preparation, characterization and potential applications* | *Kamal et al.* detail the use of barium tungstate doped with terbium ion green nanophosphor.  SENSING MECHANISM:  The sensing mechanism discussed involves the use of Barium Tungstate: 0.07Tb3+ nanophosphors embedded in a polyvinyl alcohol (PVA) solution to create fluorescent inks for anti-counterfeit markings. These nanophosphors, when dispersed in the PVA solution, enable the ink to emit specific fluorescence, which can be used for security purposes, such as identifying counterfeit items by revealing the drawn symbols under certain light conditions. 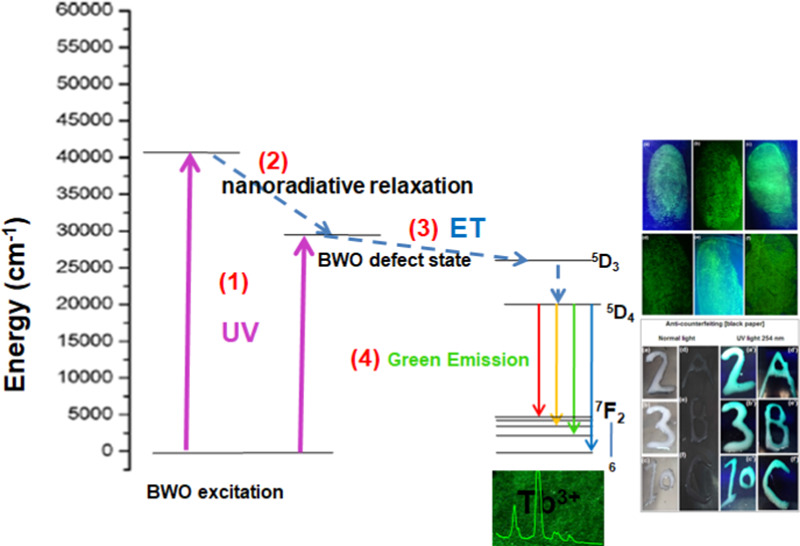 Figure 7 Graphical abstract for the optimised Nanophosphor sample (BWO: 0.07Tb3+) has been used as a photoluminescent agent for latent fingerprint detection and anti-counterfeiting applications. |
| Environmentally friendly and non-toxic material | (14) | *Nitrogen-doped carbon dot threads as a “turn-off” fluorescent probe for permanganate ions and its hydrogel hybrid as a naked eye sensor for gold(III) ions* | *Naik et al.* discuss the use of nitrogen-doped carbon dot threads as fluorescent ink in potential anti-counterfeiting applications.  SENSING MECHANISM:  This study discusses nitrogen-doped carbon dot threads that function as a "turn-off" fluorescent probe for detecting permanganate ions. The fluorescence of these carbon dots is quenched upon interaction with permanganate ions, providing a simple and effective method for sensing these ions, with a hydrogel hybrid used as a sensor for gold(III) ions based on changes in fluorescence intensity.  The approach is simple, cost-effective, and sensitive for detecting permanganate ions, offering potential applications in environmental monitoring and detection of gold(III) ions. The sensing mechanism might have limitations in terms of selectivity, as similar ions could potentially interfere with the fluorescence changes. |
|  | (15) | *Upconversion luminescence in cellulose composites (fibres and paper) modified with lanthanide-doped SrF_2_ nanoparticles* | Lanthanide-doped SrF_2_ nanoparticles combined with luminescent cellulose fibres have been developed for anti-counterfeiting applications, where they are invisible under ambient light but bright green under near-infrared light.  SENSING MECHANISM:  The study investigates the upconversion luminescence of lanthanide-doped SrF2 nanoparticles in cellulose composites. These composites exhibit luminescence when excited by specific light sources, providing a security feature for anti-counterfeiting.  The upconversion luminescence offers high sensitivity, stability, and the potential for multi-layer security features, making it useful for counterfeit detection in sensitive applications like currency.  The synthesis and integration of lanthanide-doped nanoparticles into cellulose may be complex and require specific conditions, potentially increasing production costs. This use of organic fibres is both beneficial to the environment and reduces associated costs with material manufacture. |
|  | (16) | *Metal-free and ecofriendly photoluminescent nanoparticles for visualization of latent fingerprints, anticounterfeiting, and information encryption* | *Abdollahi et al.* detail the use of metal-free and eco-friendly photoluminescent polymer nanoparticles based on oxazolidine as a sustainable alternative for anticounterfeiting.  SENSING MECHANISM:  Metal-free photoluminescent nanoparticles are used for visualizing latent fingerprints, anti-counterfeiting, and encryption. These nanoparticles emit fluorescence when exposed to light, enabling clear fingerprint visualization and serving as a versatile tool for security applications.  The nanoparticles are ecofriendly, cost-effective, and provide an efficient solution for visualizing latent fingerprints without the need for toxic chemicals, making them suitable for security and forensic applications. The method's sensitivity and reliability might be affected by environmental conditions, such as humidity or surface texture, which can impact the quality of fingerprint visualisation. |
| Review | (17) | *An Overview of Security Materials in Banknotes and Analytical Techniques in Detecting Counterfeits* | *Tomar et al.* present a broad overview of banknote security materials and analytical techniques for detecting counterfeits. They discuss new anti-counterfeiting materials and fluorescent nanoparticles that can be used as anti-counterfeiting inks with technologies such as Raman spectroscopy.  SENSING MECHANISM:  This paper reviews the use of various security materials in banknotes and analytical techniques to detect counterfeits. Techniques such as spectroscopy and microscopy are discussed for identifying counterfeit items based on advanced inks and security tags.  The paper provides a comprehensive overview of state-of-the-art methods for detecting counterfeit currency, combining innovative materials with advanced analytical techniques to enhance security. The reliance on expensive and sophisticated equipment for analysis might limit accessibility for widespread use, especially in low-resource settings. |
| Colourimetric | (18) | *Visualization and dermatoglyphics of latent fingerprints (sweat pores): Security ink for anticounterfeiting labels and case studies* | The use of colourimetric techniques to produce security ink for anticounterfeiting labels is discussed, making it easier to detect fakes and trace their origin or dispersal. 1,8-naphthalimide-based blue emitters non-covalently doped on silica have been demonstrated, with excellent results, in real-world situations.  SENSING MECHANISM:  The paper explores the use of solid-state fluorescence properties of NG (Naphthalimide) for developing a high-level security ink for anti-counterfeiting applications. The ink is prepared by dissolving NG in different solvent mixtures (ethanol, ethanol: H2O, CH3CN: H2O), with ethanol providing the best results. The thumb impressions, stamps, and handwritten text made with this ink are invisible under normal light but can be clearly visualized under UV light (365 nm), enabling secure identification of authentic documents and labels. 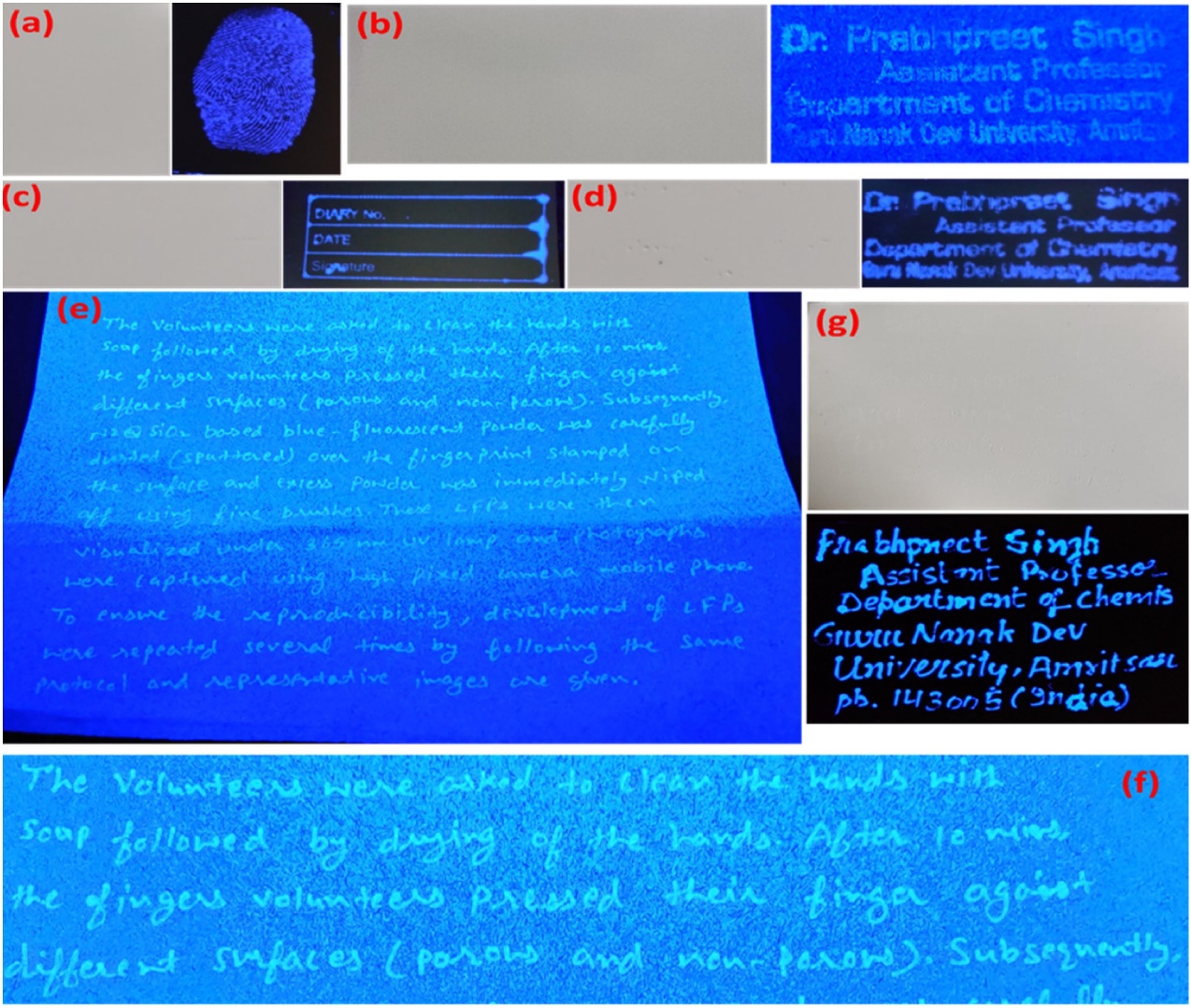 Figure 8 Images of  (a) thumb impression on [silica](https://www.sciencedirect.com/topics/materials-science/silicon-dioxide) coated [TLC](https://www.sciencedirect.com/topics/agricultural-and-biological-sciences/thin-layer-chromatography) strips before and after visualization under 365 nm lamp; stamps on (b) paper and (c,d) silica coated [TLC](https://www.sciencedirect.com/topics/materials-science/thin-layer-chromatography) strips before and after visualization under 365 nm lamp; and hand written English text using ball pen on (e,f) paper and (g) silica coated [TLC](https://www.sciencedirect.com/topics/chemical-engineering/thin-layer-chromatography) strips before and after visualization under 365 nm lamp.  The developed ink provides a highly sensitive and secure method for anti-counterfeiting, allowing for invisible markings that become visible under UV light. It is accessible for both literate and illiterate individuals, as even thumb impressions can be used for authentication. The ability to visualize text and stamps under UV light enhances security without altering the appearance of the document under normal conditions.  The reliance on UV light for visualization may limit its practical application in environments where UV lamps are not readily available. Additionally, the potential for interference from other fluorescent materials under UV light could pose challenges in distinguishing the markings, reducing the specificity of the detection method. The inks offer a non-invasive and effective solution for detecting counterfeit goods. The need for specific lighting conditions and the potential for wear or degradation over time could compromise the long-term efficacy of these security inks. |
| Chemometrics | (19) | *Forensic analysis on printer inks via chemometrics* | Novel chemometric methods are shown to provide successful forensic analysis on printer inks.  SENSING MECHANISM:  This research applies chemometrics to forensic analysis of printer inks. By analysing spectral signatures of inks, chemometric techniques can distinguish genuine prints from counterfeits.  Chemometrics offers high precision and accuracy in differentiating between genuine and counterfeit prints, providing a sophisticated and reliable method for forensic analysis. The method might be complex and require specialized knowledge and equipment, limiting its practicality for everyday use in counterfeit detection. |
|  | (20) | *Counterfeit fifty Ringgit Malaysian banknotes authentication using novel graph-based chemometrics method* | Novel chemometric methods and infrared (IR) technology has been demonstrated to provide successful forensic analysis for authentication of banknotes.  SENSING MECHANISM:  This study introduces a graph-based chemometrics method for authenticating counterfeit Malaysian banknotes. It analyses visual and chemical properties to differentiate genuine from counterfeit notes.  The method improves the security of currency verification, offering a reliable approach to detecting counterfeits based on unique chemical and visual signatures. The reliance on specific chemical properties might not be effective against highly sophisticated counterfeits that are engineered to mimic the chemical composition of genuine banknotes. |

# Pollutants

Table 4 Overview of selected pollutant sensing studies, highlighting the sensing mechanisms employed along with their respective advantages and disadvantages.

| **Theme** | **Ref.** | **Title** | **Discussion** |
| --- | --- | --- | --- |
| Eco-friendly materials | (21) | *New tyrosinases with putative action against contaminants of emerging concern.* | *Senra et al.* demonstrated the potential for replacing expensive tyrosinases (type-3 copper metalloenzymes) with cost-effective freshwater ciliates, rapid-growing unicellular microeukaryotes. They employed virtual screening to compute binding energies between 3D models of these homologs, paving the way for more economical alternatives.  SENSING MECHANISM:  This paper investigates new tyrosinases, enzymes that could be used for detecting pollutants, specifically contaminants of emerging concern. Tyrosinases, through their catalytic action, might help in sensing pollutants by facilitating the conversion of pollutants into detectable products, making them useful for environmental monitoring.  Tyrosinases are potentially highly sensitive and selective for certain pollutants, offering a biochemical route for pollutant detection that could be tailored for different contaminants. The biological nature of tyrosinases could lead to sustainable and eco-friendly sensing methods. The enzymatic approach may have limitations in terms of stability and reproducibility, as enzymes can be sensitive to environmental factors like temperature and pH. Additionally, the selectivity for specific pollutants might be a constraint in detecting a broad range of emerging contaminants. |
|  | (22) | *Electrochemical paper-based devices: sensing approaches and progress toward practical applications.* | Paper-based biosensors were highlighted as environmentally friendly alternatives to traditional substrates.  SENSING MECHANISM:  This paper explores electrochemical paper-based sensors, which can detect pollutants through electrochemical reactions. The sensors use paper as a substrate for conducting electrochemical analysis, often incorporating redox-active compounds that interact with pollutants, allowing for their detection.  Electrochemical paper-based sensors are low-cost, portable, and easy to fabricate, making them highly accessible for on-site pollutant monitoring. They offer rapid, sensitive, and specific detection, and can be tailored to detect various environmental contaminants. The performance of these sensors may be limited by the stability of the electrochemical reactions over time and under varying environmental conditions. Additionally, the sensors may require specific calibration for different pollutants, limiting their versatility for a wide range of contaminants. |
|  | (23) | *Recent Advances in Cellulose-Based Biosensors for Medical Diagnosis* | Cellulose-based biosensors were highlighted as environmentally friendly alternatives to traditional substrates.  SENSING MECHANISM:  Cellulose-based biosensors are employed for detecting pollutants by integrating biological components (like enzymes) with cellulose substrates. These biosensors can detect contaminants by producing a measurable signal in response to interactions between the contaminants and the biomolecules on the cellulose surface.  Cellulose-based biosensors are biodegradable and eco-friendly, making them a sustainable option for environmental monitoring. They can be fabricated cheaply and are flexible, which enhances their potential for portable and on-site pollutant detection.  The sensitivity of cellulose-based biosensors may be influenced by environmental factors such as humidity or temperature. Additionally, the detection range might be narrow, and more complex systems may be required for detecting a variety of pollutants simultaneously. |
| Dual detection | (24) | *Review of Thin-Layer Chromatography Tandem with Surface-Enhanced Raman Spectroscopy for Detection of Analytes in Mixture Samples* | *Zhang et al.’s* ad hoc review discussed using thin layer chromatography (TLC) coupled with SERS for on-site multi-component detection.  SENSING MECHANISM:  The TLC chromatographic plate is used for high-throughput separation with enhancement of the Raman signal through SERS, allowing for highly sensitive, quantitative detection of contaminants in complex mixtures.  The tandem technique offers high specificity and sensitivity for detecting pollutants, even at low concentrations, in complex environmental samples. The combination of TLC and SERS provides a robust and versatile method for detecting a wide range of pollutants. However, further work is needed to mature the technology for on-site applications, including the use of porous materials or polymers to enhance separation efficiency and the application of machine learning to improve the accuracy of quantitative signal information. |
|  | (25) | *Development of a Photoelectric Adjustment System with Extended Range for Fluorescence Immunochromatographic Assay Strip Readers* | Immunochromatographic assay strip readers combining immunoassay and chromatography techniques were noted demonstrating their ability to extend the range of detectable analytes  SENSING MECHANISM:  This paper focuses on a fluorescence immunochromatographic assay system for detecting pollutants. The system uses a photoelectric adjustment method to enhance the detection range, enabling the identification of contaminants through the fluorescence emitted by labelled antibodies interacting with the pollutants.  The fluorescence-based detection system offers high sensitivity, allowing for rapid and reliable detection of pollutants even at trace levels. The extended range of the photoelectric adjustment system enhances the versatility and accuracy of the assays. Fluorescence-based assays may suffer from interference due to environmental factors like light exposure or sample matrix effects. Additionally, the equipment needed for the system may not be readily available in all settings, limiting its widespread adoption for on-site pollutant detection. |
|  | (26) | *Simultaneous and rapid detection of carbofuran and 3-hydroxy-carbofuran in water samples and pesticide preparations using lateral-flow immunochromatographic assay* | Dual detection sensors simultaneously measure multiple parameters or analytes by integrating different sensing technologies or methods, enhancing accuracy, sensitivity, and versatility across various applications.  SENSING MECHANISM:  The paper discusses a lateral-flow immunochromatographic assay for the simultaneous detection of carbofuran and its hydrolyzed product, 3-hydroxy-carbofuran, in water and pesticide samples. The assay utilizes antibodies specific to the contaminants, producing a colorimetric signal when the pollutants are present.  The assay offers a rapid, cost-effective, and easy-to-use method for detecting specific pollutants in real-time. It is suitable for on-site testing of water and agricultural products, providing a straightforward approach for environmental monitoring. The sensitivity of the lateral-flow assay may be affected by the complexity of the sample matrix, leading to potential false positives or negatives. The method also may not be suitable for detecting contaminants at very low concentrations without further optimization. |
| Review | (27) | *Miniaturized analytical methods for determination of environmental contaminants of emerging concern – A review* | *Pereira et al.* present an extensive ad hoc review of miniaturised analytical methods for detecting emerging environmental contaminants (e.g. illicit drugs, surfactants and personal care products).  SENSING MECHANISM:  This review examines miniaturized analytical methods, including sensors and microfluidic devices, for detecting environmental contaminants of emerging concern. The methods utilize various detection techniques, such as electrochemical, optical, and chemical sensors, to provide fast and efficient monitoring of pollutants in environmental samples.  Miniaturized sensors are portable, cost-effective, and capable of providing rapid results, making them ideal for field-based applications. These systems also enable high-throughput screening, which is crucial for monitoring multiple contaminants simultaneously. The miniaturization of these sensors may limit their sensitivity, and they may not be suitable for detecting very low concentrations of pollutants. Additionally, the complexity of some sensors and the need for specialized equipment could hinder their widespread adoption.  They highlight opportunities for low-cost, field deployable devices with the possibility for creating big data sets at low cost, and the development of screening methods to be used before more expensive traditional sensing methods (e.g. gas chromatography-mass spectrometry) are used to validate results. However, challenges include law enforcement approval, stability of sensing elements and few commercially available set-ups. Approval from law-enforcement agencies is key in moving developed sensors from small to large-scale use. |

# Body Fluids

Table 5 Overview of selected body fluid sensing studies, highlighting the sensing mechanisms employed along with their respective advantages and disadvantages.

| **Theme** | **Ref.** | **Title** | **Discussion** |
| --- | --- | --- | --- |
| Paper-based | (28) | *Developing mitochondrial DNA field-compatible tests* | Paper-based methods show promise for on-site analysis of mitochondrial DNA and salivary amylase.  SENSING MECHANISM:  This paper focuses on the development of mitochondrial DNA (mtDNA) tests for field applications, primarily to identify individuals through biological evidence like blood or tissue samples. The method relies on extracting mitochondrial DNA from degraded samples and amplifying it for identification in forensic and clinical settings.  Portable, field-compatible tests enhance rapid identification in forensic investigations. Non-invasive and can work with small or degraded biological samples. Sensitive to contamination, which could affect accuracy. May require specialized equipment for DNA extraction and analysis, limiting widespread use in low-resource settings. |
|  | (29) | *Development of smart core-shell nanoparticle-based sensors for the point-of-care detection of alpha amylase in diagnostics and forensics* | One paper-based device using core-shell nanoparticles identifies saliva by showing a visible colour change when the shell is disrupted by alpha-amylase exposure.  SENSING MECHANISM:  This paper discusses the use of smart core-shell nanoparticles designed to detect alpha-amylase, an enzyme commonly found in body fluids such as saliva. The sensors detect alpha-amylase levels by fluorescence or colour change due to the interaction of the enzyme with the nanoparticles.  Can provide quick, on-site diagnosis, making it useful for both medical and forensic purposes. The nanoparticles enhance sensitivity and specificity for detecting low concentrations of the analyte. But, may require complex preparation and handling of nanoparticle-based materials. Stability of nanoparticles in different environmental conditions may impact the reliability of results. For mass on-site analysis further testing on human saliva samples is needed. |
|  | (23) | *Ten Years of Lateral Flow Immunoassay Technique Applications: Trends, Challenges and Future Perspectives* | *Kamal et al.* discuss recent advances in cellulose-based biosensors for medical diagnosis. The use of these alternatives present renewable, less toxic and cheaper solutions to existing sensing devices  SENSING MECHANISM:  This review highlights the development of lateral flow immunoassays (LFIA) for detecting body fluids such as saliva, urine, and blood. The sensing mechanism relies on antibodies or antigens that bind specifically to target biomarkers, which produce a visual signal (e.g., colour change) on a test strip.  Simple, rapid, and cost-effective, making it suitable for point-of-care applications. Non-invasive and requires minimal sample preparation. Lower sensitivity and specificity compared to more advanced techniques, such as PCR. May face challenges in detecting biomarkers at very low concentrations or in complex sample matrices. |
| Dual detection | (30) | *Detection of prostate specific antigen and salivary amylase in vaginal swabs using SERATEC® immunochromatographic assays* | Lateral flow immunoassays (LFIAs) offer rapid, cost-effective on-site applications, and have become widespread in recent years. In their ad hoc literature review, *Di Nardo et al.* (30) report that LFIAs have been developed to analyse prostate specific antigens (31), salivary amylase (31) and human haemoglobin (32).  SENSING MECHANISM:  This paper discusses the use of immunochromatographic assays to detect prostate-specific antigen (PSA) and salivary amylase in vaginal swabs. The assay uses antibodies specific to the target biomarkers, which generate a visible signal when the antigen binds to the antibody on the test strip.  Quick and easy detection of body fluids for forensic and diagnostic purposes. Can be used in non-laboratory settings, making it accessible for point-of-care testing. May have limited sensitivity and could produce false negatives if the concentration of the target biomarker is low. The assay may not be suitable for complex samples without proper sample pre-treatment. |
|  | (33) | *Grating-coupled surface-plasmon fluorescence DNA sensor* | Another key area of research is in the coupling of sensing technologies for a dual detection sensor. One example is the development of a grating-coupler as a transducer to excite surface-plasmon combined with fluorescence to identify DNA sequences creating quick and sensitive on-site analysis potential.  SENSING MECHANISM:  This paper presents a DNA sensor that utilizes grating-coupled surface plasmon resonance (SPR) in combination with fluorescence to detect DNA. The sensor exploits the enhanced electromagnetic field near the metal surface of a grating, which is coupled with the fluorescence of a DNA probe that binds to the target DNA sequence. This interaction leads to a detectable fluorescence signal.  High sensitivity due to the combined use of surface plasmon resonance and fluorescence. Allows for real-time, label-free detection of DNA sequences, making it suitable for various applications, including diagnostics and forensic analysis. The technique requires specialized equipment, such as a grating-coupled SPR setup, which can be expensive. The sensitivity may be influenced by environmental factors such as temperature, which can affect the accuracy of the measurements. |

# Explosives

Table 6 Overview of selected explosive sensing studies, highlighting the sensing mechanisms employed along with their respective advantages and disadvantages.

| **Theme** | **Ref.** | **Title** | **Discussion** |
| --- | --- | --- | --- |
| Dual detection | (34) | *Colorimetric-fluorescent dual-mode sensing of peroxide explosives based on inner filter effect with boosted sensitivity and selectivity* | Dual detection enhances sensitivity and selectivity, with *Cao et al.* proposing the combined use of colourimetric and fluorescent sensing with a carbon dots/titanyl sulphate (CDs/TiOSO_4_) sensing system for peroxides. The use of these dual detection methods limits the selectivity from other strong oxidants which could otherwise generate false positives.  SENSING MECHANISM:  This study introduces a dual-mode sensing system for peroxide-based explosives, utilizing both colorimetric and fluorescent methods. The sensor operates based on the inner filter effect, where the fluorescence intensity changes upon binding of the peroxide explosives, enabling highly sensitive detection. The colour change provides an easy visual confirmation, while the fluorescence offers enhanced sensitivity.  Dual-mode detection enhances both sensitivity and selectivity, making it versatile for different detection scenarios. The system offers rapid response with a visual cue, making it user-friendly and accessible for field applications. The system may be affected by environmental factors like light conditions, which could interfere with the colorimetric detection. The fluorescence-based detection might require specific equipment or settings, limiting its accessibility in some field environments. |
|  | (35) | *Ultrasensitive dual-mode visualization of perchlorate in water, soil and air boosted by close and stable Pt-Pt packing endowed low-energy absorption and emission* | *Su et al.* demonstrate colourimetric sensing alongside luminescence using a Pt(II) terpyridyl complex-based sensing platform for perchlorate detection in water, soil and air.  SENSING MECHANISM:  This paper details a dual-mode detection system for perchlorate, utilizing a novel Pt-Pt nanoparticle structure that boosts sensitivity through close and stable packing. The system employs low-energy absorption and emission mechanisms, allowing for ultrasensitive detection of perchlorate in environmental samples such as water, soil, and air.  Dual-mode visualization offers high sensitivity and the ability to detect low concentrations of perchlorate in complex environmental matrices. The stable and close Pt-Pt packing enhances both absorption and emission, improving performance across diverse conditions. The reliance on nanoparticle structures may make the system costly and difficult to scale for large-area or continuous monitoring. Environmental factors, such as high ionic strength or temperature fluctuations, may influence the system’s performance. |
|  | (36) | *The untapped potential of magnetic nanoparticles for forensic investigations: A comprehensive review* | Molecularly imprinted polymers (MIPs) offer artificial recognition sites with a fluorescent composite of carbon dots (CDs) for on-site analysis.  SENSING MECHANISM:  This review focuses on the use of magnetic nanoparticles (MNPs) for forensic detection of explosives. MNPs can selectively bind to explosive molecules, and their magnetic properties allow for easy separation and concentration of analytes, enhancing detection sensitivity in complex matrices.  Magnetic nanoparticles allow for rapid and selective separation of explosives, improving detection accuracy and speed. They offer potential for multi-analyte detection and integration into portable field devices. The synthesis of high-quality MNPs can be complex and costly. Potential interference from matrix effects or other magnetic materials in the sample may affect detection reliability. |
| 3D printing | (37) | *3D-printing pen versus desktop 3D-printers: Fabrication of carbon black/polylactic acid electrodes for single-drop detection of 2,4,6-trinitrotoluene* | 3D printing rapidly produces electrodes for electrochemical sensing. For example, *Cardoso et al.* compared 3D printing pens and desktop printers for TNT detection. A key advantage of 3D printed electrodes is that new electrode surfaces can be generated by polishing thereby enabling reuse of the sensor – another advantage over chemically-modified electrochemical sensors.  SENSING MECHANISM:  This paper compares two methods of fabricating electrochemical sensors using 3D printing: using a 3D-printing pen and a desktop 3D printer. The fabricated electrodes are designed for the detection of the explosive compound 2,4,6-trinitrotoluene (TNT) through electrochemical measurements, where the interaction of TNT with the electrode leads to a measurable current change.  The ability to fabricate sensors using 3D printing offers low-cost and customizable sensors, potentially making them more accessible for field applications. Single-drop detection simplifies the testing process, requiring minimal sample volume. The electrochemical sensor’s sensitivity may vary depending on the precision of the 3D printing process. The sensors may require calibration for different explosive compounds, potentially limiting their generalizability. |
|  | (38) | *Uranium detection by 3D-printed titanium structures: Towards decentralized nuclear forensic applications* | *Urbanová et al.* looked at the use of 3D printed titanium electrodes  SENSING MECHANISM:  This study explores the use of 3D-printed titanium structures for the detection of uranium. The titanium-based structures are sensitive to uranium ions and allow for efficient capture and detection through electrochemical signals, offering a portable method for nuclear forensic applications.  3D-printed titanium offers a low-cost, customizable approach for uranium detection, particularly useful in field applications. The system provides a direct, rapid detection method for uranium without requiring complex laboratory equipment. The effectiveness of the detection system may be influenced by the environmental conditions or the presence of other ions. The specific focus on uranium limits its use for detecting other explosives or hazardous materials. |
|  | (7) | *Promising Applications of Additive-Manufactured (3D-printed) Electrochemical Sensors for Forensic Chemistry* | *Castro et al.* completed an ad hoc review of 3D-printed electrochemical sensors showing the great promise they have for portable, on-site analysis.  SENSING MECHANISM:  This paper explores the use of 3D-printed electrochemical sensors for forensic applications, particularly in the detection of explosives and other hazardous materials. The sensors are made from conductive materials such as carbon or metal-based inks, which change their electrochemical properties upon interaction with target compounds, allowing for the detection of explosives like TNT.  3D printing offers the ability to quickly and cost-effectively produce customized sensors for various explosive compounds. The portability and versatility of these sensors make them suitable for on-site forensic investigations. The performance and accuracy of 3D-printed sensors can vary depending on the quality of the 3D printing process. Long-term stability and durability of 3D-printed sensors may be a concern for repeated use in the field. |
| Real-time monitoring | (39) | *Free-standing, thin-film sensors for the trace detection of explosives* | Continuous real-time monitoring of many explosives has been explored using a free-standing thin-film sensor relying on the catalytic decomposition of the explosive and its heat effects.  SENSING MECHANISM:  This paper discusses free-standing, thin-film sensors designed for the trace detection of explosives. These sensors are capable of detecting minute quantities of explosive compounds based on the interaction of the analytes with the thin film material, which results in a measurable change in electrical or optical properties.  The thin-film design allows for highly sensitive and rapid detection of trace amounts of explosives. The free-standing nature of the sensors makes them easy to integrate into portable detection devices. Thin-film sensors can be sensitive to environmental factors such as humidity and temperature, which may reduce their reliability. The materials used for the thin films may need to be optimized to increase sensor lifespan and stability under real-world conditions. |
| Review | (40) | *Field-portable and handheld laser-induced breakdown spectroscopy: Historical review, current status and future prospects* | *Senesi et al.* reviewed laser-induced breakdown spectroscopy (LIBS), a chemical elemental analysis technique which is found to be a sensitive and selective sensing technology suitable for on-site measurement  SENSING MECHANISM:  This review covers laser-induced breakdown spectroscopy (LIBS) as a tool for detecting explosives, where a laser is used to create a plasma on the sample surface, and the emitted light is analysed to identify the chemical composition of the explosive. LIBS is particularly valuable for detecting trace amounts of explosives in real-time without the need for sample preparation.  LIBS provides rapid, real-time analysis of explosives with no need for sample preparation, making it ideal for field use. It is capable of detecting a wide range of explosive materials with high sensitivity. The technique can be affected by interference from background materials or environmental conditions, potentially reducing detection accuracy. High power requirements and the need for a laser source make it less portable and more expensive compared to other detection methods. |

# Poison/ Toxin

Table 7 Overview of selected poison/ toxin sensing studies, highlighting the sensing mechanisms employed along with their respective advantages and disadvantages.

| **Theme** | **Ref.** | **Title** | **Discussion** |
| --- | --- | --- | --- |
| AIE (Aggregation-induced emission) | (11) | *Lighting up forensic science by aggregation-induced emission: A review* | The use of AIE phenomenon on filter paper strips detecting volatile poisons and pesticides has been demonstrated, though further research is needed for detecting poisons in body fluids and universal AIE probes for group recognition.  SENSING MECHANISM:  This review explores Aggregation-Induced Emission (AIE)-based fluorescent probes, which light up upon aggregation, making them useful for detecting biological and chemical toxins, including blood-based toxins or residues. AIE luminogens are used in trace detection and imaging of biological fluids, cell activity, and toxic agents. AIE luminogens undergo restricted intramolecular motions upon interaction with biological analytes, causing enhanced fluorescence for detection of trace toxicants in body fluids or tissues.  High sensitivity and photostability, ideal for long-term imaging or trace detection. Low background interference due to emission only upon aggregation. May require synthetic customization for specific analytes. Some AIE materials are costly or not yet field-deployable. |
|  | (21) | *New tyrosinases with putative action against contaminants of emerging concern* | A recognition element that detects classes of poisons would reduce sensing time and cost.  SENSING MECHANISM:  The paper discusses novel tyrosinase enzymes that can interact with and potentially break down various toxic organic compounds, such as pharmaceuticals, pesticides, or industrial toxins. The implication is their use in biosensors that recognize and degrade contaminants via enzymatic oxidation. Tyrosinases catalyse oxidation of phenolic contaminants, generating quinones that can be detected via electrochemical or colorimetric signals.  Biocatalytic degradation potential, offering both detection and neutralization. High specificity for phenolic or aromatic compounds. Enzyme stability under varied environmental conditions can be an issue. Not suitable for real-time detection unless immobilized or integrated into devices. |
|  | (30) | *Developing mitochondrial DNA field-compatible tests* | An ad hoc review of LFIAs details their use in the rapid on-site detection of viruses (41,42), toxins (43–47), mycotoxins (48–53), bacteria (54–58), allergens (59,60) and pesticides (61–64).  SENSING MECHANISM:  This study focuses on the development of field-deployable mitochondrial DNA (mtDNA) tests, which can be indirectly used to assess cell damage or poisoning by identifying degraded or altered DNA profiles, potentially caused by toxins. Field-compatible PCR and amplification methods for mitochondrial DNA allow detection of toxin-induced degradation or alterations in mtDNA sequences.  Highly sensitive to cellular stress or degradation. Portable, allowing use in field settings for forensic and ecological toxin exposure assessments. Not specific to individual toxins—more of a biomarker for damage than direct detection. Requires careful sample handling and extraction protocols. |
|  | (28) | *Ten Years of Lateral Flow Immunoassay Technique Applications: Trends, Challenges and Future Perspectives* | Paper-based on-site methods for pathogen detection using isothermal nucleic acid amplification are demonstrated.  SENSING MECHANISM:  This review includes Lateral Flow Immunoassays (LFIAs) used to detect toxins, drugs, or biological markers via antigen-antibody binding and colorimetric detection. For toxins, specific antibodies can detect proteins or small molecules from poisons or biological agents. Lateral flow immunoassays (LFIA) detect toxin-associated antigens via immobilized antibodies; visual readout via colloidal gold or fluorescent labels.  Rapid, user-friendly, and portable, suitable for field deployment. Relatively low cost and does not require extensive training. Limited sensitivity compared to lab-based methods. Can be prone to false positives/negatives without confirmatory testing. |
|  | (65) | *Sensitive and selective detections of mustard gas and its analogues by 4-mercaptocoumarins as fluorescent chemosensors in both solutions and gas phase* | Fast response time has been demonstrated using two fluorescent probe molecules (4-mercaptocoumarins) in a test strip to detect mustard gas and its analogues with a three minute response time and high sensitivity.  SENSING MECHANISM:  Utilises fluorescent chemosensors based on 4-mercaptocoumarins to detect mustard gas and analogues. The sensors fluoresce upon interaction with toxic compounds due to structural changes in the molecule. 4-mercaptocoumarin chemosensors react with mustard gas analogues via thiol-alkylation, inducing fluorescence quenching or enhancement.  Highly sensitive and selective, capable of detecting trace levels in both gas and liquid phases. Works under dual-phase conditions, improving versatility. May require UV excitation sources for fluorescence, which can limit portability. Performance may degrade in complex environments like battlefield or disaster zones. |
| Gaseous Target | (66) | *Smart forensic kit: Real-time estimation of postmortem interval using a highly sensitive gas sensor for microbial forensics* | *Shin et al.* developed a sensitive colorimetric gas sensor with a smartphone-based analysis for real-time quantitative detection of bacterial-derived ammonia gas, helping determine the postmortem interval (PMI). Such on-site sensing overcomes previous challenges of bacterial growth whilst the body is being moved which can produce false PMIs.  SENSING MECHANISM:  Introduces a gas sensor that detects volatile organic compounds (VOCs) produced by microbial activity postmortem—many of which are toxic gases (e.g., cadaverine, putrescine). The kit estimates the postmortem interval (PMI) using these toxins as markers. Metal oxide semiconductor gas sensor detects microbial VOCs (e.g., NH₃, H₂S) emitted during decomposition, correlating with postmortem interval and presence of toxic gases.  Real-time monitoring with high sensitivity to trace toxic gases. Useful for both forensic and death investigations in varied environments. Sensor performance can be influenced by ambient environmental factors like humidity or temperature. Cross-sensitivity to other VOCs may affect specificity. |
|  | (67) | *Bifunctional Fluorescent Probes for the Detection of Mustard Gas and Phosgene* | Multiplexed gas sensing is discussed with several papers looking at the use of bifunctional fluorescent probes. These probes, equipped with two sensing sites, yield two distinct fluorescence responses upon exposure to either of the two target analytes, ranging from various nerve agent stimulants to mustard gas and phosgene.  SENSING MECHANISM:  This paper presents bifunctional fluorescent probes capable of detecting both mustard gas and phosgene. The dual recognition and response mechanism is triggered by interaction with the electrophilic groups in these gases, causing fluorescence changes. Bifunctional fluorescent probes undergo nucleophilic substitution with mustard gas/phosgene, altering electron distribution and emission spectra.  Enables dual detection in a single probe, enhancing utility in multi-threat environments. Offers visual, real-time results, aiding rapid response. May require controlled lighting conditions to observe fluorescence accurately. Synthetic complexity of bifunctional probes may hinder large-scale production. |

# Fingerprints

Table 8 Overview of selected fingerprint sensing studies, highlighting the sensing mechanisms employed along with their respective advantages and disadvantages.

| **Themes** | **Ref.** | **Title** | **Discussion** |
| --- | --- | --- | --- |
| Environmentally friendly | (68) | *Box-Behnken design optimisation of a green novel nanobio-based reagent for rapid visualisation of latent fingerprints on wet, non-porous substrates* | The development of environmentally friendly, non-toxic sensors was a key issue identified for mass production. *Azman et al.* proposed using a lipase from Candida rugosa (CRL) as a greener option for fingerprint analysis on wet substrates. Although CRL is used in various scientific applications (69–71), its use in fingerprint visualisation is limited, presenting an exciting research opportunity.  SENSING MECHANISM:  A green nanobio-based reagent optimized via Box-Behnken design combines curcumin with biodegradable polymers and biosurfactants. It interacts with lipid and amino acid residues in latent prints, forming visible contrast through hydrophobic and electrostatic interactions on wet, non-porous surfaces.  Environmentally sustainable and non-toxic, ideal for eco-conscious forensics. Effective on difficult surfaces (e.g., glass, metal) even when wet. Visual development is rapid and doesn’t require UV or advanced imaging. Limited stability over time due to natural reagent degradation. May show variability across substrates and with different residue compositions. Lower contrast compared to fluorescent methods. |
| Advances in nanomaterials | (72) | *The untapped potential of magnetic nanoparticles for forensic investigations: A comprehensive review* | A review of magnetic nanoparticles (MNPs) to conjugate with quantum dots (QDs) for fluorescence properties has been presented. Unlike traditional powders used, the small size of MNPs were found to be efficient in selectively binding to fingerprints and not the background  SENSING MECHANISM:  Magnetic nanoparticles (Fe₃O₄ and hybrids) enhance fingerprint contrast by binding to residues through van der Waals forces, hydrogen bonding, or charge-based interactions, often combined with optical or fluorescence readouts.  Easy recovery and reuse due to magnetic separation. Surface can be tailored for specificity (e.g., via functional ligands or dyes). Can be used in combination with fluorescent, colorimetric, or electrochemical readouts. Nanoparticles tend to aggregate, reducing efficiency and uniformity. Requires careful storage and stabilization. Can be more expensive and labour-intensive to produce compared to traditional powders. |
|  | (73) | *Synthesis of gold nanoparticles immobilized on fibrous nano‐silica for latent fingerprints detection* | Gold nanoparticles on fibrous nano-silica enhance ridge details and sweat pores on various surfaces with properties of low-cost, easy preparation, chemical stability and great affinity to finger residues.  SENSING MECHANISM:  Gold nanoparticles (AuNPs) immobilized on fibrous nano-silica adhere to fingerprint residues via affinity for proteins and lipids, enhancing visibility due to localized surface plasmon resonance (LSPR).  Provides high sensitivity and contrast under ambient or dark-field imaging. Nanostructured silica increases surface area for interaction and retention. Good chemical stability of AuNPs enhances shelf life. Synthesis is multi-step and can be expensive. Some background signal from silica may interfere under fluorescence. Requires controlled humidity or environmental conditions for optimal results. |
|  | (74) | *Recent progress of fluorescent materials for fingermarks detection in forensic science and anti-counterfeiting.* | *Ansari et al.* review applied nanomaterials and luminescent Ln^3+^ NPs/ upconversion (UC) NPs which provide higher contrast, sensitivity and selectivity which is lacking in most of the traditional fluorescent nanomaterials used. However, more studies are needed to improve the efficiency, performance, surface-functionality and biocompatibility of these Ln^3+^ NPs/ UCNPs for fingerprint recognition.  SENSING MECHANISM:  Fluorescent metal complexes, quantum dots, and organic dyes interact with fingerprint residues via hydrogen bonding, hydrophobicity, or electrostatic attraction, emitting under specific wavelengths for enhanced imaging.  Enables visualization on complex, multi-coloured, or porous surfaces. Useful for old or degraded prints due to high signal sensitivity. Can be multiplexed for anti-counterfeiting or dual imaging purposes. Some dyes are photobleachable or environmentally sensitive. Health or environmental toxicity of certain materials (e.g., Cd-based QDs). Requires UV or specific excitation sources for visualization. |
| Carbon dots (CDs) | (75) | *Applications of Carbon Dots (CDs) in Latent Fingerprints Imaging* | Carbon dots (CDs) present exceptional characteristics such as high fluorescence, non-toxicity, eco-friendliness, stability and cost-effectiveness compared to traditional methods. *Shabashini et al.* present an ad hoc review of publications relating to the application of CDs but of importance here is the enhancement of fingerprint visualisation, using magnetic composite powder CDs, due to the abundant surface hydrophilic groups (76). Low-cost, superparamagnetic fluorescence performance and excellent safety makes these CDs suitable candidates for on-site visualisation. However, issues remain to transition from proof of concept to field application.  SENSING MECHANISM:  Carbon Dots (CDs) bind to fingerprint residues via surface functional groups (e.g., –COOH, –NH₂), leading to strong blue or green fluorescence under UV due to quantum confinement and surface defect emission.  Simple, low-cost synthesis from green precursors (e.g., citric acid, urea). High fluorescence quantum yield with good photostability. Non-toxic and biocompatible for safe handling. Background fluorescence from certain surfaces (e.g., plastics) may interfere. Fluorescence can vary based on dot size or surface passivation. Long-term stability of the fluorescence can degrade without proper storage. |
|  | (76) | *A user-secure and highly selective enhancement of latent fingerprints by magnetic composite powder based on carbon dot fluorescence* | Carbon dots (CDs) present exceptional characteristics such as high fluorescence, non-toxicity, eco-friendliness, stability and cost-effectiveness compared to traditional methods. *Shabashini et al.* present an ad hoc review of publications relating to the application of CDs but of importance here is the enhancement of fingerprint visualisation, using magnetic composite powder CDs, due to the abundant surface hydrophilic groups (76). Low-cost, superparamagnetic fluorescence performance and excellent safety makes these CDs suitable candidates for on-site visualisation. However, issues remain to transition from proof of concept to field application.  SENSING MECHANISM:  Magnetic carbon dot composites provide dual functionality: fluorescence via CDs and easy recovery via magnetic components. The composite binds selectively to sweat residues in ridges through electrostatic and hydrophobic interactions.  Dual functionality with fluorescence for visual imaging, magnetism for easy clean-up. Selective enhancement of ridge detail due to surface interactions with CDs. Reduces background interference and makes the method user-friendly. Synthesis requires careful control of carbon dot surface chemistry and magnetic core integration. Requires fluorescence imaging setup (UV source, filters). Material cost and preparation complexity higher than conventional fingerprint powders. |

# Food Safety

Table 9 Overview of selected food safety sensing studies, highlighting the sensing mechanisms employed along with their respective advantages and disadvantages.

| **Theme** | **Ref.** | **Title** | **Discussion** |
| --- | --- | --- | --- |
| Paper-device | (77) | *Diameter-based inkjet-printed paper devices for formaldehyde analysis in foods* | *Tasangtong et al.* discuss inkjet-printed paper devices for rapid, portable and eco-friendly formaldehyde analysis in foods.  SENSING MECHANISM:  The study presents inkjet-printed paper-based analytical devices (PADs) for formaldehyde detection in food via a diameter-based colorimetric approach. The devices utilize the Nash reagent (ammonium acetate and acetylacetone) which reacts with formaldehyde to form a yellow diacetyldihydrolutidine product, with the intensity and spread of colour correlating to concentration. 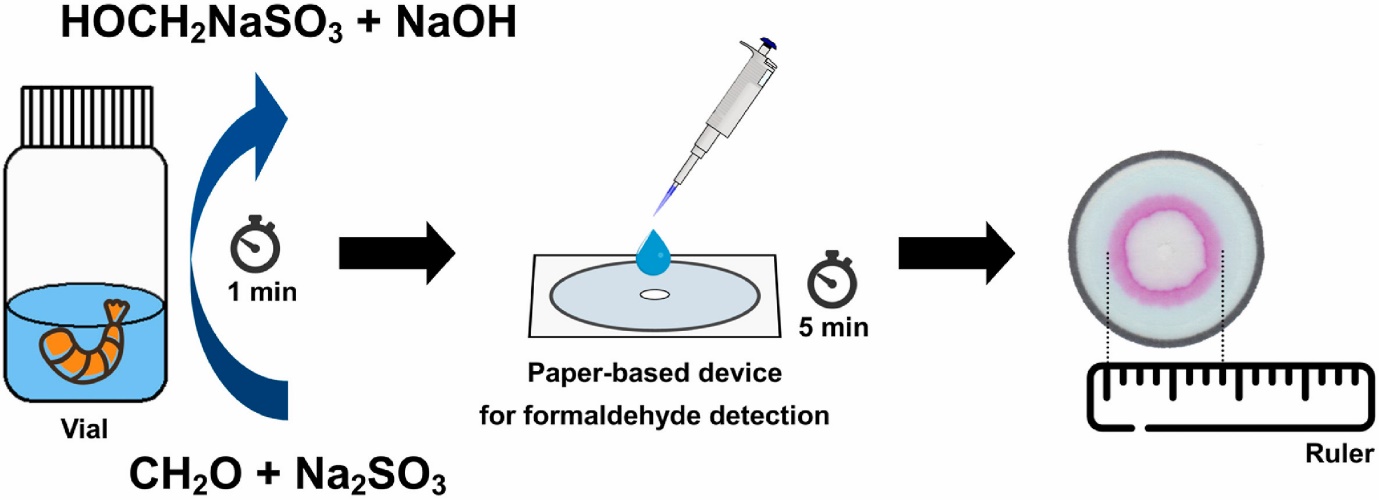 Figure 9 Graphical abstract demonstrating the use of PADs as a diameter-based colourimetric approach.  Inexpensive, easy-to-fabricate, and portable for on-site testing. Visual diameter readout allows semi-quantitative analysis without instruments. Susceptible to environmental humidity affecting wicking diameter. Limited precision and not suitable for trace-level formaldehyde quantification. |
| 3D printed electrodes | (78) | *3D-printing in forensic electrochemistry: Atropine determination in beverages using an additively manufactured graphene-polylactic acid electrode* | 3D-printing of graphene-polylactic acid electrodes for atropine detection in beverages offers low-cost, reproducible, large-scale sensor production and shows great promise for developing other electrochemical sensors for analytes commonly found at crime scenes.  SENSING MECHANISM:  Developed a 3D-printed electrochemical sensor using graphene-polylactic acid composite electrodes for atropine detection. The sensor exploits atropine's electroactive properties via cyclic voltammetry for quantification in spiked beverage samples.  Cost-effective and customizable sensor platform using additive manufacturing. High sensitivity and selectivity for atropine in complex matrices. Graphene-PLA electrodes may require surface treatment for optimal conductivity. Reusability and long-term stability of printed electrodes may be limited. |
| Dual detection | (79) | *Fabrication of an electrochemical biodevice for ractopamine detection under a strategy of a double recognition of the aptamer/molecular imprinting polymer* | *Roushani et al.* discuss a double recognition strategy using MIP and aptamer on a carbon electrode to sense ractopamine, a molecule commonly used in livestock feed, sometimes inappropriately or excessively. This strategy can be extended to other target analytes by the simple exchange of the relevant aptamer.  SENSING MECHANISM:  Fabricated a dual-recognition electrochemical sensor using an aptamer and molecularly imprinted polymer (MIP) for ractopamine detection. The synergy of aptamer specificity and MIP selectivity enhances signal response in square wave voltammetry.  Dual-recognition system provides excellent selectivity even in complex samples. Aptamer-MIP combination improves reproducibility and target specificity. Fabrication is complex and may hinder rapid deployment or scalability. Stability of aptamers under varied storage/field conditions is a concern. |
|  | (80) | *Silver nanoparticle-decorated TiO₂ nanotube array for solid-phase microextraction and SERS detection of antibiotic residue in milk* | The paper highlights antibiotic degradation detection in milk using silver nanoparticle-decorated TiO_2_ for solid-phase microextraction (SPME) and SERS. SPME is a new sample preparation technique that simplifies extraction and reduces sample loss. Silver nanoparticles have greater SERS activity than traditional silver sol. In combination, SPME-SERS provides rapid on-site detection.  SENSING MECHANISM:  Designed a SERS-active substrate by decorating TiO₂ nanotube arrays with silver nanoparticles for the solid-phase microextraction and detection of antibiotic residues in milk. The platform enhances Raman signals of extracted analytes.  Extremely high sensitivity due to localized surface plasmon resonance effects. Integration with SPME improves analyte preconcentration and detection limits. Requires precise nanomaterial synthesis, limiting mass production. Matrix interferences from milk may complicate quantitative analysis. |
|  | (81) | *A fluorescence based immunochromatographic sensor for monitoring chlorpheniramine and its comparison with a gold nanoparticle-based lateral-flow strip* | Antibody-based sensing technologies are common in food safety analysis offering low-cost and rapid detection. Development of a fluorescent immunochromatographic strip assay based on a chlorpheniramine (CPM) antibody in the detection of CPM, a harmful illegal additive in teas and health foods is discussed.  SENSING MECHANISM:  Developed a fluorescence-based immunochromatographic assay (FICA) for chlorpheniramine detection using fluorescently labelled antibodies and compared it with a gold nanoparticle-based LFA. Detection was based on specific antigen-antibody binding.  Fluorescence readout offers higher sensitivity and lower detection limits than traditional gold LFAs. Rapid detection, suitable for field use and screening. Requires fluorescence reader, increasing equipment dependency. Shelf life of fluorescent conjugates can be shorter than gold nanoparticles. |
|  | (82) | *Fluorescence based immunochromatographic sensor for rapid and sensitive detection of tadalafil and comparison with a gold lateral flow immunoassay* | A LFIA based on a fluorescence and gold nanoparticles labelled antibody for Tadalafil (a banned additive found in beverages) recognition is also presented. The strip is observed under ultra-violet light and can be completed within ten-minutes making it perfect for on-site analysis  SENSING MECHANISM:  Developed a Fluorescence Immunochromatographic Assay (FICA) for tadalafil detection using lanthanide chelate-labelled antibodies and compared it to a gold-based lateral flow assay. Fluorescence intensity directly correlates to the concentration of bound tadalafil.  High sensitivity and specificity for detecting low tadalafil concentrations. Good comparison with traditional methods, showing improved detection limits. Fluorescent readers add cost and may limit use in resource-limited settings. Cross-reactivity with structurally similar PDE-5 inhibitors may affect specificity. |

# Illicit Drugs

Table 10 Overview of selected illicit drug sensing studies, highlighting the sensing mechanisms employed along with their respective advantages and disadvantages.

| **Theme** | **Ref.** | **Title** | **Discussion** |
| --- | --- | --- | --- |
| Portability, Affordability and Ease of Use | (83–85) | *Wearable Electrochemical Sensors for the Monitoring and Screening of Drugs. (89)*  *Perspective and application of modified electrode material technology in electrochemical voltammetric sensors for analysis and detection of illicit drugs. (90)*  *Nanoarchitectonics for Abused-Drug Biosensors. (91)* | Key to successful implementation of roadside drug testing is portability, with many review papers (83–85) suggesting further work that needs to be done to achieve this.  SENSING MECHANISM:  **(89)** This paper discusses wearable electrochemical sensors that employ enzymatic or molecularly selective layers (e.g., aptamers, ion-selective membranes) on flexible electrodes to detect drug metabolites in biofluids such as sweat, saliva, or interstitial fluid. Detection is commonly achieved through voltammetry or amperometry, which transduce molecular recognition into electrical signals.  Enables non-invasive, continuous drug monitoring in real time, improving compliance and personalised care. Miniaturised and flexible designs are suitable for on-body applications and remote health tracking. Susceptible to signal drift and biofouling from complex biofluid matrices. Sensitivity and specificity can be challenged by inter-individual variability and cross-reactivity with metabolites or other substances.  **(90)** This paper highlights the use of nanostructured electrode modifiers—such as metal nanoparticles, graphene, and conducting polymers—to enhance electrochemical voltammetric detection of drugs. These modifications increase surface area and electron transfer rates, allowing for highly sensitive measurements of electroactive illicit substances like cocaine or methamphetamine.  High signal amplification leads to ultra-low detection limits and fast response times. Selective surface chemistry can be tuned to target specific drugs, improving analytical accuracy. Nanomaterial synthesis and electrode fabrication can be complex and costly for routine forensic deployment. Some materials may suffer from instability under environmental or storage conditions, impacting reproducibility.  **(91)** This review explores the concept of nanoarchitectonics—precise molecular and nanostructure engineering—to create biosensing platforms incorporating materials such as metal–organic frameworks (MOFs), mesoporous structures, and carbon nanomaterials. These platforms integrate recognition elements (e.g., antibodies, aptamers) with nanostructured transducers for specific and amplified detection of drug molecules.  Combines ultra-sensitive nanomaterials with biomolecular specificity, achieving both selectivity and low detection thresholds. Supports multiplexed sensing formats and integration with portable or wearable electronics. Some nanoarchitectured systems remain in early development and lack scalability for real-world use. Stability and bio-compatibility of hybrid materials in practical biosensing environments need further validation. Let me know if you'd like this formatted into a table or document |
|  | (86) | *Recent advances in developing optical and electrochemical sensors for analysis of methamphetamine: A review* | A pivotal review article surveys literature spanning the last two decades, focusing on optical and electrochemical sensing technologies for analysing methamphetamine. Many low-cost sensing technologies have been outlined from fluorescence to electro-chemiluminescence highlighting the range of existing and potential low-cost sensing platforms for methamphetamine. Application of these developed sensing technologies to other drug sensing provides a low-cost, high-yield route for further sensing development.  SENSING MECHANISM:  This review covers both electrochemical and optical sensing platforms for methamphetamine detection. Electrochemical sensors often rely on modified electrodes (e.g., carbon-based nanomaterials, metal nanoparticles) for voltammetric or amperometric detection via oxidation of methamphetamine at the electrode surface. Optical methods include colorimetric, fluorescence, and surface plasmon resonance (SPR) techniques using molecular recognition elements such as molecularly imprinted polymers (MIPs) or aptamers to induce detectable changes in optical signals upon binding to the target.  Electrochemical sensors offer high sensitivity, low cost, and potential for portable use in field settings or point-of-care analysis. Optical methods provide fast response times, visual outputs, and are often suitable for high-throughput or non-contact measurements. Incorporation of nanomaterials enhances both sensitivity and selectivity across techniques. Electrochemical sensors may suffer from electrode fouling and matrix interference, reducing long-term stability in complex samples like blood or urine. Optical sensors can be sensitive to environmental conditions (e.g., pH, light interference), and often require complex calibration and fabrication processes to ensure specificity for methamphetamine over similar compounds. |
|  | (87) | *Sensitive and reliable identification of fentanyl citrate in urine and serum using chloride ion-treated paper-based SERS substrate* | SERS technology on a paper-based substrate enables on-site detection, such as for fentanyl citrate in serum and urine. This method utilises a paper-based SERS substrate embedded with chloride ion treated gold nanospheres, with SERS spectra collected using a portable Raman spectrometer (87).  SENSING MECHANISM:  This study develops a surface-enhanced Raman scattering (SERS) substrate by treating paper with chloride ions to modulate the morphology and plasmonic properties of silver nanoparticles. This enhances signal uniformity and reproducibility for detecting fentanyl citrate. The analyte interacts with the SERS-active surface, producing characteristic Raman peaks that allow for trace-level identification in complex biological matrices.  The chloride-modified SERS substrate enables ultrasensitive and specific detection of fentanyl in bodily fluids, even at trace levels. Paper-based format allows low-cost, portable testing suitable for field use or rapid diagnostics. Improved substrate uniformity increases signal reproducibility, addressing a common limitation in SERS. SERS signals are still influenced by sample matrix effects, requiring pre-treatment or purification for complex biological fluids. Substrate fabrication, while low-cost, requires strict control of ion treatment conditions to maintain reproducibility and sensitivity across batches. |
|  | (25) | *Development of a Photoelectric Adjustment System with Extended Range for Fluorescence Immunochromatographic Assay Strip Readers* | The development of a fluorescence immunochromatographic assay (FICA) strip reader provides low cost, user-friendly, highly sensitive and rapid detection capabilities, enabling convenient on-site testing (25). This sensor incorporates a photoelectric adjustment system, leveraging the linear correlation between fluorescence and excitation light intensity, enabling precise tuning of the excitation light intensity. Such adjustment broadens the potential detection range for target analytes. This technology will have implications in many sensing devices for various target analytes on-site.  SENSING MECHANISM:  This work presents an enhanced fluorescence immunochromatographic assay (FICA) system integrating a photoelectric adjustment mechanism. The system improves detection by optimising alignment and signal acquisition from fluorescently labelled antibodies bound to analytes on test strips. The platform uses photodiodes and dynamic calibration to maintain accuracy over a broader signal range, improving consistency in lateral flow-based immunoassays.  The improved reader allows higher sensitivity and broader dynamic range, making it more suitable for detecting low-abundance analytes. Automated signal adjustment reduces user error and enhances reproducibility in point-of-care or on-site settings. Compatible with existing FICA platforms and adaptable for various analytes. The system’s complexity and cost may limit widespread use, especially in resource-limited settings. Hardware dependency reduces portability and requires regular maintenance or calibration for consistent performance. |
|  | (88) | *Tackling the Problem of Sensing Commonly Abused Drugs Through Nanomaterials and (Bio)Recognition Approaches* | *Truta et al.* show that electrochemical methods can rapidly determine drugs with rapid, sensitive, selective detection in complex human matrices (*e.g.* blood, urine or saliva) and are easily miniaturised for on-site use. However, the electrochemical sensing of illicit drugs so far has been limited to academic research – no commercial market appears to have been conquered yet.  SENSING MECHANISM:  This review explores the use of nanomaterials (e.g., carbon nanostructures, metal nanoparticles, quantum dots) combined with biorecognition elements like aptamers, antibodies, and enzymes for detecting abused drugs. Detection strategies include electrochemical, fluorescent, and colorimetric platforms, often with nanomaterial-enhanced sensitivity due to increased surface area and signal amplification.  Nanomaterials offer high sensitivity, low detection limits, and rapid response times, ideal for forensic or roadside screening. Versatile platforms allow integration into portable, wearable, or point-of-care formats. Wide compatibility with various drug targets and sample types. Reproducibility and stability of nanomaterial-based sensors remain a key challenge in real-world applications. Biorecognition elements may be susceptible to denaturation or matrix interferences, especially in biological samples. |
|  | (89) | *Recent developments in electrochemical detection of illicit drugs in diverse matrices* | Square-wave voltammetry (SWV) is the most widely used voltammetry technology for facile and rapid quantitative sensing of illicit drugs (89). The choice of electrode, especially the working electrode, can have a large impact on a sensor’s sensitivity. *De Rycke et al.* (89) predict that carbon paste electrodes will further gain popularity and be used in most electrochemical sensors for the detection of illicit drugs as they are flexible in design with the possibility for miniaturisation.  SENSING MECHANISM:  This review focuses on electrochemical detection of illicit drugs using modified electrodes (e.g., with carbon nanomaterials, conducting polymers, or molecularly imprinted polymers) to enhance selectivity and sensitivity. Drug molecules are typically oxidised or reduced at the electrode surface, and the resultant current is proportional to concentration.  Electrochemical techniques are cost-effective, rapid, and suitable for miniaturisation, facilitating field deployment. Electrodes can be tailored to specific drugs for selective and multiplexed detection. Capable of analysing complex matrices like urine, saliva, and blood with minimal sample preparation. Electrode fouling and non-specific adsorption may interfere with performance and require frequent maintenance. Matrix effects and overlapping redox peaks in multi-drug scenarios can reduce accuracy without extensive optimisation. |
|  | (90) | *Recent advances in the direct electrochemical detection of drugs of abuse* | The presence on the market of cheap and disposable electrochemical cells, namely screen-printed electrodes, has made feasible the creation of effective devices for the quantification of illicit drugs in an on-site screening test (90).  SENSING MECHANISM:  This paper highlights progress in direct electrochemical detection, particularly using unmodified or minimally modified electrodes for analysing drugs like cocaine, heroin, and amphetamines. Detection relies on intrinsic electroactivity of target molecules, captured via voltammetric techniques.  Simplified sensor design avoids need for bioreceptors or complex functionalisation. Offers fast, low-cost detection and can be implemented in disposable formats. Direct sensing enables use in resource-limited or on-site environments. Lower selectivity due to interfering electroactive species in real samples. Not suitable for drugs lacking electroactive functional groups, limiting universality. |
|  | (91) | *Electrochemical Sensing of Cannabinoids in Biofluids: A Noninvasive Tool for Drug Detection* | Nanomaterials, with their unique properties and low cost, enhance electrochemical sensor sensitivity for detecting drugs in low concentrations. However, future electrochemical sensor development needs to consider the implementation of biocompatible and environmentally friendly materials (91). Many articles discuss the potential of paper-based portable sensors to aid police enforcement (22,87,92–102).  SENSING MECHANISM:  This review discusses electrochemical sensors targeting cannabinoids like THC and CBD in saliva, sweat, and urine. Methods involve voltammetry and amperometry, often employing nanostructured electrodes to improve signal transduction. Detection typically focuses on oxidation reactions of cannabinoid molecules.  Enables non-invasive sampling (e.g., saliva, sweat), improving user compliance and rapid field testing. Sensors are adaptable for wearable technologies, allowing continuous monitoring. Use of nanomaterials enhances sensitivity and limit of detection. Low analyte concentration in some biofluids may require pre-concentration or amplification steps. Cannabinoid metabolites and structurally similar compounds can cause cross-reactivity, complicating quantification. |
|  | (103) | *Recent Advances in Cellulose-Based Biosensors for Medical Diagnosis* | Cellulose is also discussed as a potential low-cost, environmentally friendly supporting material for biosensors, whose high number of hydroxyl functional groups provide the ability for the construction of novel materials for new advanced biosensor-based applications (103).  SENSING MECHANISM:  This review focuses on cellulose-based biosensors, particularly those utilising cellulose nanofibres or nanocrystals as the sensing matrix. These materials support immobilisation of bioreceptors (e.g., antibodies, enzymes) and facilitate optical or electrochemical detection. Their high surface area and modifiable hydroxyl groups enable strong analyte interaction and signal transduction.  Cellulose is biodegradable, biocompatible, and abundant, making it a sustainable base material. High surface functionality allows for stable bioreceptor immobilisation and enhanced sensitivity. Compatible with wearable formats and flexible electronics for real-time health or forensic diagnostics. Mechanical and environmental stability (e.g., under high humidity or chemical exposure) can be a concern. Batch-to-batch variations in natural cellulose sources may affect sensor reproducibility and calibration. Requires precise surface modification techniques to control bioreceptor orientation and activity. |
|  | (22) | *Electrochemical paper-based devices: sensing approaches and progress toward practical applications* | To reduce errors of inference by non-experts (i.e. most police officers do not have chemistry degrees), on-site drug tests would need to be simple to operate and interpret. Therefore, recent publications for on-site testing have focused on the simplification of current systems. Research includes the design of data processing software to simplify measurements (22), potential for smartphone control of sensors (110), and the development of smartphone apps for interpreting data (111).  SENSING MECHANISM:  This review details the use of electrochemical paper-based analytical devices (ePADs), which combine porous paper substrates with printed electrodes to perform sensing via techniques like voltammetry, amperometry, and potentiometry. The paper substrate acts as a fluidic transport medium, while electrodes are functionalised with recognition elements (e.g., enzymes, aptamers, MIPs) for target-specific electrochemical readout.  ePADs are low-cost, lightweight, and suitable for point-of-care or field use, including forensic screening. The use of paper allows for capillary-driven flow, eliminating the need for external pumps. Devices are easily fabricated with methods like screen printing or wax printing and can be disposed of sustainably. Paper's inherent variability and porosity can lead to signal inconsistency and lower reproducibility. Limited sample pre-treatment or volume control makes it challenging to process complex matrices without interference. Integration with electronic readers or multiplexing functions can be technically demanding. |
|  | (104) | *A smartphone-based colorimetric assay using Au@Ag core-shell nanoparticles as the nanoprobes for visual tracing of fluvoxamine in biofluids as a common suicide drug* | SENSING MECHANISM:  The paper describes a smartphone-based colorimetric assay using Au@Ag core-shell nanoparticles as nanoprobes for visual tracing of fluvoxamine, a common suicide drug. The sensing mechanism involves the reduction of Au@Ag nanoparticles in the presence of fluvoxamine, changing the colour of the solution, which is then detected by a smartphone-based camera system.  The method provides a rapid, cost-effective, and portable approach for detecting fluvoxamine in biofluids. It leverages smartphone technology for real-time data collection, enhancing accessibility and ease of use. The sensitivity and accuracy of the method may be influenced by the complexity of the biofluid matrix, requiring precise calibration for reliable results. Additionally, it may not differentiate between fluvoxamine and structurally similar compounds. |
|  | (105) | *Generic sensor platform based on electro-responsive molecularly imprinted polymer nanoparticles (e-NanoMIPs)* | SENSING MECHANISM:  The authors present a sensor platform based on electro-responsive molecularly imprinted polymer nanoparticles (e-NanoMIPs). These nanoparticles selectively capture illicit drug molecules, and their electrochemical properties change in response to drug binding, producing a measurable signal.  The e-NanoMIPs offer high selectivity and sensitivity for detecting illicit drugs, with the ability to adapt to various drug types by tailoring the polymer imprinting. This platform is versatile, enabling both laboratory and field use The fabrication of molecularly imprinted polymers can be complex and time-consuming, and the sensor's performance may be influenced by the presence of interfering substances in complex samples |
| Matrix Tolerance and Interferents | (90) | *Recent advances in the direct electrochemical detection of drugs of abuse* | Roadside drug sensors will require detection in a human matrix which introduces issues of interference and may require sample pre-treatment (95)  SENSING MECHANISM:  *See above* |
|  | (106) | *Rapid Drop-Volume Electrochemical Detection of the “Date Rape” Drug Flunitrazepam in Spirits Using a Screen-Printed Sensor in a Dry-Reagent Format* | Testing in a greater range of potential matrices to make technologies field ready is attractive (106).  SENSING MECHANISM:  This paper discusses a rapid drop-volume electrochemical detection method for the "date rape" drug flunitrazepam in spirits using a screen-printed sensor with a dry-reagent format. The electrochemical detection relies on the redox reaction of flunitrazepam at the electrode surface, producing a measurable current.  The method offers fast, sensitive, and on-site detection of flunitrazepam, especially useful in forensic and emergency settings. The screen-printed sensor is cost-effective and portable, ideal for point-of-use testing. The sensor’s performance could be affected by the alcohol content in spirits, requiring adjustments in sensor calibration. Additionally, it may not be as sensitive for detecting low concentrations of flunitrazepam compared to more traditional laboratory methods. |
|  | (107) | *Electrochemiluminescent screening for methamphetamine metabolites* | Electro-chemiluminescent screening using a Nafion film on a glassy carbon electrode is a sensing technique that requires little to no extraction or sample preparation. This provides ideal implementation for on-site screening in serum, urine and saliva.  SENSING MECHANISM:  Electrochemiluminescent (ECL) screening is used to detect methamphetamine metabolites. The ECL reaction occurs when methamphetamine metabolites interact with a luminophore, producing light, which is then measured to determine drug concentration.  This method is highly sensitive and specific, offering low detection limits for methamphetamine metabolites. ECL is a non-invasive, rapid, and cost-effective technique ideal for field applications. The need for specialized equipment for detecting the emitted light limits the portability of the method. Additionally, the technique may be affected by matrix interferences, requiring careful sample preparation. |
|  | (108) | *Selective Sensing of THC and Related Metabolites in Biofluids by Host:Guest Arrays* | Selective discrimination of illicit drugs and their metabolites is a key theme to be explored for many sensing devices.  SENSING MECHANISM:  The paper discusses the use of host:guest arrays for selective sensing of THC and related metabolites in biofluids. The host molecules selectively bind to THC or its metabolites, causing a shift in the fluorescence signal, which can be measured.  The method offers high selectivity and sensitivity for detecting THC and its metabolites, even at low concentrations. It is non-invasive, suitable for biofluid analysis, and provides quick results. The sensitivity may decrease in the presence of other similar compounds, making it challenging to differentiate THC from other cannabinoids without additional modifications. The approach may require optimization for different biofluid types. |
| Specificity | (109) | *The Future of Analytical and Interpretative Toxicology: Where are We Going and How Do We Get There?* | For roadside drug testing, high specificity is needed to ensure the fair apprehension of suspects. Many drugs, due to degradation, are identified through their metabolites in body fluids.  SENSING MECHANISM:  This paper discusses the future directions for analytical and interpretative toxicology, with a focus on advanced methods for detecting illicit drugs. The methods include biosensors, chromatography, and mass spectrometry, all offering distinct mechanisms for identifying and quantifying drugs of abuse.  The integration of various techniques promises improved detection capabilities and sensitivity, enabling more reliable and faster toxicology analysis in complex matrices. The need for sophisticated and often expensive equipment, along with the potential for complex sample preparation, can limit accessibility in some forensic and emergency settings. |
|  | (110) | *Recent biosensing advances in the rapid detection of illicit drugs* | Technologies outlined to improve specificity include the use of novel recognition elements such as aptamers – which provide a cheap method to bind to target groups enabling sensing – and the combination of two sensor elements.  SENSING MECHANISM:  The review focuses on the latest advances in biosensors for rapid detection of illicit drugs. Techniques such as electrochemical, optical, and biorecognition-based sensors are highlighted, with emphasis on their ability to detect a variety of illicit drugs quickly and efficiently.  The biosensors discussed offer rapid, specific, and highly sensitive detection of illicit drugs, making them ideal for real-time monitoring and on-site testing. Some of the techniques require complex instrumentation and may be prone to matrix effects, which could lead to false positives or negatives unless carefully calibrated. |
| Multiplexing | (89,91,111) | *Recent developments in electrochemical detection of illicit drugs in diverse matrices. (94)*  *Electrochemical Sensing of Cannabinoids in Biofluids: A Noninvasive Tool for Drug Detection. (96)*  *Electrochemical sensors targeting salivary biomarkers: A comprehensive review. (117)* | Multiplex detection is increasingly important, allowing simultaneous analysis of multiple analytes (89,91,111). This will be vital for on-site drug tests as there are many potential target drugs that need to be identified. Paper-based sensors have the potential for multiplexed detection and will be important for on-site application (22). Lateral flow tests provide rapidity, simplicity, relative cost-effectiveness, and the possibility to be used by non-skilled personnel. However, drawbacks include possible cross-reactivity, matrix interference and (easy) manipulation by users (*e.g.* the use of soda to cause a false positive). A lateral flow immunoassay combines multiple lines to increase detection capability, where each line contains a specific recognition element for different target analytes.  SENSING MECHANISM:  Electrochemical sensors targeting salivary biomarkers are explored as non-invasive tools for illicit drug detection. These sensors use electrochemical reactions triggered by drug metabolites in saliva, which generate a measurable signal.  The non-invasive nature of the sensors, along with their ability to provide rapid and real-time results, makes them suitable for field applications, particularly in law enforcement and roadside testing. Saliva composition can vary significantly between individuals, potentially influencing the sensor’s accuracy. Additionally, low concentrations of drugs may be difficult to detect without highly sensitive sensors. |
|  | (112) | *Ten Years of Lateral Flow Immunoassay Technique Applications: Trends, Challenges and Future Perspectives* | However, the addition of multiple recognition sites and therefore lateral flow lines requires an increased sample volume, higher fabrication costs and increased reagent use.  SENSING MECHANISM:  The paper reviews the lateral flow immunoassay technique (LFIA) for detecting illicit drugs. In this method, antibodies specific to the drug of interest are immobilized on a strip, and the drug presence is detected through a colour change as it binds to the antibody.  LFIAs are cost-effective, easy to use, and offer rapid results, making them ideal for point-of-care and field applications. They can be adapted for multiple drugs simultaneously in a single test. LFIAs may lack sensitivity compared to more complex methods, and false negatives or positives can occur due to cross-reactivity or improper handling of samples. |

# Other

Table 11 Overview of selected sensing studies relating to other target analytes not covered by the main categories, highlighting the sensing mechanisms employed along with their respective advantages and disadvantages.

| **Ref.** | **Title** | **Discussion** |
| --- | --- | --- |
| (113) | *Neutron and Gamma-Ray Detection System Coupled to a Multirotor for Screening of Shipping Container Cargo* | Many publications focussed on the detection of radioactive materials (113,114). This study highlighted the advantages of silicon photomultipliers in beta and gamma detectors over current radiation monitors used at seaports, citing their lightweight, compact design, and lower power consumption.  SENSING MECHANISM:  The study introduces a mobile radiation detection system mounted on a multirotor drone, utilizing an EJ-200 plastic scintillator to detect gamma rays and neutrons. The system captures radiation signatures from shipping containers to identify illicit materials.  Enhances safety by enabling remote detection without human exposure. Increases efficiency in scanning large or hard-to-reach areas.​ Potential regulatory and operational challenges in deploying drones in certain airspaces. Limited payload capacity may restrict the size and sensitivity of detection equipment.​ |
| (113,114) | *Development of a CsI(Tl) scintillator based gamma probe for the identification of nuclear materials in unknown areas. Journal of Instrumentation* | SENSING MECHANISM:  *Kim et al.* developed a gamma probe based on a CsI(Tl) scintillator coupled with a photodetector to identify nuclear materials. The scintillator emits light upon gamma-ray interaction, with the intensity correlating to radiation levels.​  High detection efficiency and energy resolution for gamma-ray identification. Compact design suitable for field deployment in unknown areas.​ CsI(Tl) scintillators can be hygroscopic, requiring protective encapsulation. Potential background radiation may affect measurement accuracy |
| (115) | *A Fabrication of Multichannel Graphite Electrode Using Low-Cost Stencil-Printing Technique* | Emphasis was placed on developing environmentally friendly alternatives to current sensing technologies, including sustainable printed electrochemical platforms (115) and paper-based microfluidic devices (116).  SENSING MECHANISM:  Fabricated multichannel graphite electrodes using a stencil-printing technique on polypropylene substrates. These electrodes facilitate simultaneous electrochemical measurements for analyte detection. ​  Cost-effective and straightforward fabrication process. Multichannel design allows for parallel analysis, increasing throughput.​ Stencil-printing may result in variability between electrodes, affecting reproducibility. Graphite electrodes may exhibit lower conductivity compared to other materials, impacting sensitivity.​ |
| (116) | *Paper-based microfluidic devices: On-site tools for crime scene investigation* | SENSING MECHANISM:  Reviewed the application of paper-based microfluidic devices for on-site forensic analysis. These devices utilize capillary action to transport samples to reaction zones for colorimetric or electrochemical detection. ​  Portable, inexpensive, and easy to use, making them suitable for fieldwork. Require minimal sample volumes and reduce the need for complex instrumentation.​ Limited sensitivity and quantitative capabilities compared to laboratory-based methods. Potential issues with sample evaporation and environmental contamination. |
| (117) | *Mobile Raman spectroscopy analysis of elephant ivory objects* | Portability emerged as a key theme, with innovations such as a mobile fibre-optics Raman spectrometer addressing challenges of dispersive Raman spectroscopy and potentially enabling mobile spectroscopy applications, such as for elephant ivory.  SENSING MECHANISM:  Sensing Mechanism: Employed mobile Raman spectroscopy to analyse elephant ivory artifacts non-destructively. The technique uses laser light scattering to obtain molecular fingerprints of the material. ​  Allows in situ analysis without damaging valuable artifacts. Provides specific molecular information aiding in authentication and conservation.​ Fluorescence interference from the sample can obscure Raman signals. Portable Raman instruments may have lower sensitivity compared to benchtop models. |
| (124–134) |  | Additionally, several ad hoc reviews were identified (124–134), covering diverse topics such as 3D electrodes in electrochemical sensing (125), SERS studies on saliva (131) and advances in SERS for molecular sensing (132). |
| (125) | *3-D Electrodes for Electrochemical Sensors: Review in Different Approaches* | SENSING MECHANISM:  Reviewed various approaches for creating 3D electrodes in electrochemical sensors, including 3D printing and templating techniques. These electrodes enhance analytical performance by increasing surface area. ​  3D electrodes provide improved sensitivity and lower detection limits due to increased active sites. Customization through advanced fabrication techniques allows for tailored sensor designs.​ Fabrication methods like 3D printing may introduce structural inconsistencies affecting reproducibility. Integration with existing sensor platforms may require additional optimization. |
| (131) | *Methods in Raman spectroscopy for saliva studies - a review* | SENSING MECHANISM:  This review explores various Raman spectroscopy methods applied to the analysis of saliva, focusing on vibrational fingerprinting through inelastic light scattering. Techniques such as conventional Raman, surface-enhanced Raman spectroscopy (SERS), and fibre-optic Raman setups are discussed in the context of biomarker detection and disease diagnosis from saliva matrices.  Raman spectroscopy allows for non-destructive, label-free analysis of complex biological fluids like saliva. Minimal sample preparation is needed, and the technique can be adapted for portable and point-of-care systems. Saliva sampling is non-invasive, increasing compliance in forensic or clinical settings. Saliva's variable composition and fluorescence background can interfere with Raman signal quality. SERS requires careful substrate preparation and can suffer from reproducibility issues due to hotspot variability and inconsistent nanoparticle distribution. Conventional Raman has low sensitivity, often necessitating enhancement techniques or advanced instrumentation. |
| (132) | *Progress in surface enhanced Raman scattering molecular sensing: A review* | SENSING MECHANISM:  This review outlines advancements in surface-enhanced Raman scattering (SERS) for molecular sensing, where analyte molecules are adsorbed onto plasmonic nanostructures (typically gold or silver), significantly amplifying Raman signals via localized surface plasmon resonance (LSPR). It discusses substrate design, fabrication methods, and target molecule applications.  SERS offers ultra-sensitive detection, capable of single-molecule level analysis under optimized conditions. High molecular specificity enables clear spectral identification in complex samples. Can be applied to a wide range of analytes including drugs, explosives, and biological markers relevant in forensic investigations. Fabrication of reproducible and stable SERS-active substrates remains a major technical challenge. Quantitative analysis is difficult due to signal variation from inconsistent hotspot distribution and signal fluctuations. Sensitivity can degrade over time due to oxidation or degradation of metallic nanostructures, especially under environmental conditions. |

# References

1. Shalini Devi KS, Anantharamakrishnan A, Maheswari Krishnan U. Expanding Horizons of Metal Oxide-based Chemical and Electrochemical Sensors. Electroanalysis [Internet]. 2021;33(9):1979–96. Available from: https://onlinelibrary.wiley.com/doi/full/10.1002/elan.202100087

2. Castro SVF, Lima AP, Rocha RG, Cardoso RM, Montes RHO, Santana MHP, et al. Simultaneous determination of lead and antimony in gunshot residue using a 3D-printed platform working as sampler and sensor. Anal Chim Acta. 2020 Sep 15;1130:126–36.

3. Promsuwan K, Kanatharana P, Thavarungkul P, Limbut W. Nitrite amperometric sensor for gunshot residue screening. Electrochim Acta. 2020 Jan 20;331:135309.

4. McKeever C, Callan S, Warren S, Dempsey E. Magnetic nanoparticle modified electrodes for voltammetric determination of propellant stabiliser diphenylamine. Talanta. 2022;238.

5. Priya S, Jain VK, Suman N. Gunshot residue detection technologies—a review. Egypt J Forensic Sci [Internet]. 2021 Dec;11(1). Available from: https://www.proquest.com/scholarly-journals/gunshot-residue-detection-technologies-review/docview/2535751703/se-2?accountid=14511

6. Harshey A, Srivastava A, Das T, Nigam K, Shrivastava R, Yadav VK. Trends in Gunshot Residue Detection by Electrochemical Methods for Forensic Purpose. Journal of Analysis and Testing 2021 5:3 [Internet]. 2021 Jan 31 [cited 2021 Sep 4];5(3):258–69. Available from: https://link.springer.com/article/10.1007/s41664-020-00152-x

7. Castro SVF, Rocha RG, Joao AF, Richter EM, Munoz RAA. Promising Applications of Additive-Manufactured (3D-printed) Electrochemical Sensors for Forensic Chemistry. BRAZILIAN JOURNAL OF ANALYTICAL CHEMISTRY. 2022;9(34):79–105.

8. Chedid AA, Azevedo LS, Galaço A, Casagrande TR, Serra OA, de Oliveira MF. Voltammetric analysis of luminescent markers in gunshot residues. J Forensic Sci. 2023;68(3):780–9.

9. Senesi GS, Harmon RS, Hark RR. Field-portable and handheld laser-induced breakdown spectroscopy: Historical review, current status and future prospects. Spectrochim Acta Part B At Spectrosc. 2021 Jan 1;175:106013.

10. Nadar SS, Kelkar RK, Pise P V, Patil NP, Patil SP, Chaubal-Durve NS, et al. The untapped potential of magnetic nanoparticles for forensic investigations: A comprehensive review. Talanta. 2021;230.

11. Yan Y, Zhang J, Yi S, Liu L, Huang C. Lighting up forensic science by aggregation-induced emission: A review. Anal Chim Acta. 2021 Apr 22;1155:238119.

12. Verhagen A, Kelarakis A. Carbon Dots for Forensic Applications: A Critical Review. NANOMATERIALS. 2020;10(8).

13. Kamal R, Saif M. Barium tungstate doped with terbium ion green nanophosphor: Low temperature preparation, characterization and potential applications. SPECTROCHIMICA ACTA PART A-MOLECULAR AND BIOMOLECULAR SPECTROSCOPY. 2020;229.

14. Naik VM, Gunjal DB, Gore AH, Anbhule P V, Sohn D, Bhosale S V, et al. Nitrogen-doped carbon dot threads as a “turn-off” fluorescent probe for permanganate ions and its hydrogel hybrid as a naked eye sensor for gold(III) ions. Anal Bioanal Chem. 2020;412(12):2993–3003.

15. Szczeszak A, Skwierczynska M, Przybylska D, Runowski M, Smiechowicz E, Erdman A, et al. Upconversion luminescence in cellulose composites (fibres and paper) modified with lanthanide-doped SrF2 nanoparticles. J Mater Chem C Mater. 2020;8(34):11922–8.

16. Abdollahi A, Dashti A, Rahmanidoust M, Hanaei N. Metal-free and ecofriendly photoluminescent nanoparticles for visualization of latent fingerprints, anticounterfeiting, and information encryption. SENSORS AND ACTUATORS B-CHEMICAL. 2022;372.

17. Tomar A, Gupta RR, Mehta SK, Sharma S. An Overview of Security Materials in Banknotes and Analytical Techniques in Detecting Counterfeits. Crit Rev Anal Chem. 2023;

18. Kumar S, Singh P. Visualization and dermatoglyphics of latent fingerprints (sweat pores): Security ink for anticounterfeiting labels and case studies. JOURNAL OF PHOTOCHEMISTRY AND PHOTOBIOLOGY A-CHEMISTRY. 2023;437.

19. Paxton N, Smolan W, Böck T, Mary Francis B, Sophia Ponraj J, Dhanabalan B, et al. Forensic analysis on printer inks via chemometrics. IOP Conf Ser Mater Sci Eng [Internet]. 2021 Nov 1 [cited 2024 Jan 9];1192(1):012029. Available from: https://iopscience.iop.org/article/10.1088/1757-899X/1192/1/012029

20. Nurfarhana H, Tahir A, Mahat NA, Hasmerya M, Keat HF. Counterfeit fifty Ringgit Malaysian banknotes authentication using novel graph-based chemometrics method. Scientific Reports (Nature Publisher Group) [Internet]. 2022;12(1). Available from: https://www.proquest.com/scholarly-journals/counterfeit-fifty-ringgit-malaysian-banknotes/docview/2641737027/se-2?accountid=14511

21. Senra MVX, Fonseca AL. New tyrosinases with putative action against contaminants of emerging concern. Proteins: Structure, Function, and Bioinformatics. 2021 Sep 1;89(9):1180–92.

22. Noviana E, McCord CP, Clark KM, Jang I, Henry CS. Electrochemical paper-based devices: sensing approaches and progress toward practical applications. Lab Chip. 2019 Dec 17;20(1):9–34.

23. Kamel S, Khattab TA. Recent Advances in Cellulose-Based Biosensors for Medical Diagnosis. Biosensors (Basel). 2020 Jun 17;10(6):67.

24. Zhang M, Yu Q, Guo J, Wu B, Kong X. Review of Thin-Layer Chromatography Tandem with Surface-Enhanced Raman Spectroscopy for Detection of Analytes in Mixture Samples. Biosensors (Basel) [Internet]. 2022;12(11). Available from: https://pubmed.ncbi.nlm.nih.gov/36354446/

25. Wu H, Gao Y, Yang J, Vai M, Du M, Pun S. Development of a Photoelectric Adjustment System with Extended Range for Fluorescence Immunochromatographic Assay Strip Readers. IEEE Photonics J. 2021 Jun 1;13(3).

26. Lan J, Sun W, Chen L, Zhou H, Fan Y, Diao X, et al. Simultaneous and rapid detection of carbofuran and 3-hydroxy-carbofuran in water samples and pesticide preparations using lateral-flow immunochromatographic assay. Food Agric Immunol. 2020 Jan 1;31(1):165–75.

27. Pena-Pereira F, Bendicho C, Pavlović DM, Martín-Esteban A, Díaz-Álvarez M, Pan Y, et al. Miniaturized analytical methods for determination of environmental contaminants of emerging concern – A review. Anal Chim Acta. 2021;1158:238108.

28. Dhar BC, Roche CE, Levine JF. Developing mitochondrial DNA field-compatible tests. https://doi.org/101080/1064338920211904709. 2021;

29. Adhikary RR, Banerjee R. Development of smart core-shell nanoparticle-based sensors for the point-of-care detection of alpha amylase in diagnostics and forensics. Biosens Bioelectron. 2021 Jul 15;184:113244.

30. Nardo F Di, Chiarello M, Cavalera S, Baggiani C, Anfossi L. Ten Years of Lateral Flow Immunoassay Technique Applications: Trends, Challenges and Future Perspectives. Sensors. 2021 Jul 30;21(15):5185.

31. Kishbaugh JM, Cielski S, Fotusky A, Lighthart S, Maguire K, Quarino L, et al. Detection of prostate specific antigen and salivary amylase in vaginal swabs using SERATEC® immunochromatographic assays. Forensic Sci Int. 2019 Nov 1;304:109899.

32. Murahashi M, Makinodan M, Yui M, Hibi T, Kobayashi M. Immunochromatographic detection of human hemoglobin from deteriorated bloodstains due to methamphetamine contamination, aging, and heating. Analytical and Bioanalytical Chemistry 2020 412:23 [Internet]. 2020 Jul 8 [cited 2021 Sep 4];412(23):5799–809. Available from: https://link.springer.com/article/10.1007/s00216-020-02802-6

33. Kasry A, Nicol A, Knoll W. Grating-coupled surface-plasmon fluorescence DNA sensor. Applied Physics B 2021 127:5 [Internet]. 2021 Apr 23 [cited 2021 Aug 23];127(5):1–12. Available from: https://link.springer.com/article/10.1007/s00340-021-07619-4

34. Cao HY, Cai ZZ, Li YS, Wang GF, Dou XC. Colorimetric-fluorescent dual-mode sensing of peroxide explosives based on inner filter effect with boosted sensitivity and selectivity. CHINESE JOURNAL OF ANALYTICAL CHEMISTRY. 2022;50(1):4–12.

35. Su Z, Li YS, Li JG, Li K, Dou XC. Ultrasensitive dual-mode visualization of perchlorate in water, soil and air boosted by close and stable Pt-Pt packing endowed low-energy absorption and emission. J Mater Chem A Mater. 2022;10(15):8195–207.

36. Nadar SS, Kelkar RK, Pise P V., Patil NP, Patil SP, Chaubal-Durve NS, et al. The untapped potential of magnetic nanoparticles for forensic investigations: A comprehensive review. Talanta. 2021 Aug 1;230:122297.

37. Cardoso RM, Rocha DP, Rocha RG, Stefano JS, Silva RAB, Richter EM, et al. 3D-printing pen versus desktop 3D-printers: Fabrication of carbon black/polylactic acid electrodes for single-drop detection of 2,4,6-trinitrotoluene. Anal Chim Acta. 2020 Oct 2;1132:10–9.

38. Urbanová V, Pumera M. Uranium detection by 3D-printed titanium structures: Towards decentralized nuclear forensic applications. Appl Mater Today. 2020;21.

39. Ricci PP, Gregory OJ. Free-standing, thin-film sensors for the trace detection of explosives. Scientific Reports 2021 11:1 [Internet]. 2021 Mar 23 [cited 2021 Aug 25];11(1):1–10. Available from: https://www.nature.com/articles/s41598-021-86077-6

40. Senesi GS, Harmon RS, Hark RR. Field-portable and handheld laser-induced breakdown spectroscopy: Historical review, current status and future prospects. Spectrochim Acta Part B At Spectrosc. 2021 Jan 1;175:106013.

41. Couturier C, Wada A, Louis K, Mistretta M, Beitz B, Povogui M, et al. Characterization and analytical validation of a new antigenic rapid diagnostic test for Ebola virus disease detection. PLoS Negl Trop Dis [Internet]. 2020 Jan 1 [cited 2021 Sep 4];14(1):e0007965. Available from: https://journals.plos.org/plosntds/article?id=10.1371/journal.pntd.0007965

42. DeMers HL, He S, Pandit SG, Hannah EE, Zhang Z, Yan F, et al. Development of an antigen detection assay for early point-of-care diagnosis of Zaire ebolavirus. PLoS Negl Trop Dis [Internet]. 2020 Nov 1 [cited 2021 Sep 4];14(11):e0008817. Available from: https://journals.plos.org/plosntds/article?id=10.1371/journal.pntd.0008817

43. Wu KH, Huang WC, Shyu RH, Chang SC. Silver nanoparticle-base lateral flow immunoassay for rapid detection of Staphylococcal enterotoxin B in milk and honey. J Inorg Biochem. 2020 Sep 1;210:111163.

44. Li Y, Xu X, Liu L, Kuang H, Xu L, Xu C. A gold nanoparticle-based lateral flow immunosensor for ultrasensitive detection of tetrodotoxin. Analyst [Internet]. 2020 Mar 16 [cited 2021 Sep 4];145(6):2143–51. Available from: https://pubs.rsc.org/en/content/articlehtml/2020/an/d0an00170h

45. Bever CS, Adams CA, Hnasko RM, Cheng LW, Stanker LH. Lateral flow immunoassay (LFIA) for the detection of lethal amatoxins from mushrooms. PLoS One [Internet]. 2020 Apr 1 [cited 2021 Sep 4];15(4):e0231781. Available from: https://journals.plos.org/plosone/article?id=10.1371/journal.pone.0231781

46. Pan M, Ma T, Yang J, Li S, Liu S, Wang S. Development of Lateral Flow Immunochromatographic Assays Using Colloidal Au Sphere and Nanorods as Signal Marker for the Determination of Zearalenone in Cereals. Foods 2020, Vol 9, Page 281 [Internet]. 2020 Mar 4 [cited 2021 Sep 4];9(3):281. Available from: https://www.mdpi.com/2304-8158/9/3/281/htm

47. Xu S, Zhang G, Fang B, Xiong Q, Duan H, Lai W. Lateral Flow Immunoassay Based on Polydopamine-Coated Gold Nanoparticles for the Sensitive Detection of Zearalenone in Maize. ACS Appl Mater Interfaces [Internet]. 2019 Aug 28 [cited 2021 Sep 4];11(34):31283–90. Available from: https://pubs.acs.org/doi/abs/10.1021/acsami.9b08789

48. Li R, Meng C, Wen Y, Fu W, He P. Fluorometric lateral flow immunoassay for simultaneous determination of three mycotoxins (aflatoxin B1, zearalenone and deoxynivalenol) using quantum dot microbeads. Microchimica Acta 2019 186:12 [Internet]. 2019 Nov 6 [cited 2021 Sep 4];186(12):1–9. Available from: https://link.springer.com/article/10.1007/s00604-019-3879-6

49. Huang X, Huang X, Xie J, Li X, Huang Z. Rapid simultaneous detection of fumonisin B1 and deoxynivalenol in grain by immunochromatographic test strip. Anal Biochem. 2020 Oct 1;606:113878.

50. Li X, Wu X, Wang J, Hua Q, Wu J, Shen X, et al. Three lateral flow immunochromatographic assays based on different nanoparticle probes for on-site detection of tylosin and tilmicosin in milk and pork. Sens Actuators B Chem. 2019 Dec 12;301:127059.

51. Wang Z, Hu S, Bao H, Xing K, Liu J, Xia J, et al. Immunochromatographic assay based on time-resolved fluorescent nanobeads for the rapid detection of sulfamethazine in egg, honey, and pork. J Sci Food Agric [Internet]. 2021 Jan 30 [cited 2021 Sep 4];101(2):684–92. Available from: https://onlinelibrary.wiley.com/doi/full/10.1002/jsfa.10681

52. Wang Z, Wu X, Liu L, Xu L, Kuang H, Xu C. Rapid and sensitive detection of diclazuril in chicken samples using a gold nanoparticle-based lateral-flow strip. Food Chem. 2020 May 15;312:126116.

53. Byzova NA, Serchenya TS, Vashkevich II, Zherdev A V., Sviridov O V., Dzantiev BB. Lateral flow immunoassay for rapid qualitative and quantitative control of the veterinary drug bacitracin in milk. Microchemical Journal. 2020 Jul 1;156:104884.

54. Zhuang L, Gong J, Ji Y, Tian P, Kong F, Bai H, et al. Lateral flow fluorescent immunoassay based on isothermal amplification for rapid quantitative detection of Salmonella spp. Analyst [Internet]. 2020 Mar 16 [cited 2021 Sep 4];145(6):2367–77. Available from: https://pubs.rsc.org/en/content/articlehtml/2020/an/c9an02011j

55. Ilhan H, Tayyarcan EK, Caglayan MG, Boyaci İH, Saglam N, Tamer U. Replacement of antibodies with bacteriophages in lateral flow assay of Salmonella Enteritidis. Biosens Bioelectron. 2021 Oct 1;189:113383.

56. He D, Wu Z, Cui B, Xu E, Jin Z. Establishment of a dual mode immunochromatographic assay for Campylobacter jejuni detection. Food Chem. 2019 Aug 15;289:708–13.

57. Anfossi L, Di Nardo F, Russo A, Cavalera S, Giovannoli C, Spano G, et al. Silver and gold nanoparticles as multi-chromatic lateral flow assay probes for the detection of food allergens. Analytical and Bioanalytical Chemistry 2018 411:9 [Internet]. 2018 Nov 6 [cited 2021 Sep 4];411(9):1905–13. Available from: https://link.springer.com/article/10.1007/s00216-018-1451-6

58. Wang Y, Li Z, Lin H, Siddanakoppalu PN, Zhou J, Chen G, et al. Quantum-dot-based lateral flow immunoassay for the rapid detection of crustacean major allergen tropomyosin. Food Control. 2019 Dec 1;106:106714.

59. Galan-Malo P, Pellicer S, Pérez MD, Sánchez L, Razquin P, Mata L. Development of a novel duplex lateral flow test for simultaneous detection of casein and β-lactoglobulin in food. Food Chem. 2019 Sep 30;293:41–8.

60. Zhang M, Li M, Zhao Y, Xu N, Peng L, Wang Y, et al. Novel monoclonal antibody-sandwich immunochromatographic assay based on Fe3O4/Au nanoparticles for rapid detection of fish allergen parvalbumin. Food Research International. 2021 Apr 1;142:110102.

61. Ge W, Suryoprabowo S, Kuang H, Liu L, Song S. Rapid detection of triazophos in cucumber using lateral flow immunochromatographic assay. https://doi.org/101080/0954010520201816919 [Internet]. 2020 Jan 1 [cited 2021 Sep 4];31(1):1051–60. Available from: https://www.tandfonline.com/doi/abs/10.1080/09540105.2020.1816919

62. Cevallos-Cedeño RE, Agulló C, Abad-Fuentes A, Abad-Somovilla A, Mercader J V. Enzyme and lateral flow monoclonal antibody-based immunoassays to simultaneously determine spirotetramat and spirotetramat-enol in foodstuffs. Scientific Reports 2021 11:1 [Internet]. 2021 Jan 19 [cited 2021 Sep 4];11(1):1–13. Available from: https://www.nature.com/articles/s41598-021-81432-z

63. Wu KH, Huang WC, Chang SC, Kao CH, Shyu RH. Colloidal silver-based lateral flow immunoassay for rapid detection of melamine in milk and animal feed. Mater Chem Phys. 2019 Jun 1;231:121–30.

64. Chen Q, Qie M, Peng X, Chen Y, Wang Y. Immunochromatographic assay for melamine based on luminescent quantum dot beads as signaling probes. RSC Adv [Internet]. 2020 Jan 16 [cited 2021 Sep 4];10(6):3307–13. Available from: https://pubs.rsc.org/en/content/articlehtml/2020/ra/c9ra08350b

65. Xue MJ, Wei XZ, Feng W, Xing ZF, Liu SL, Song QH. Sensitive and selective detections of mustard gas and its analogues by 4-mercaptocoumarins as fluorescent chemosensors in both solutions and gas phase. J Hazard Mater. 2021 Aug 15;416.

66. Shin J, Song YG, Jung SJ, Yoon T, Kim GS, Kim JH, et al. Smart forensic kit: Real-time estimation of postmortem interval using a highly sensitive gas sensor for microbial forensics. SENSORS AND ACTUATORS B-CHEMICAL. 2020;322.

67. Feng W, Liu XJ, Xue MJ, Song QH. Bifunctional Fluorescent Probes for the Detection of Mustard Gas and Phosgene. Anal Chem. 2023;

68. Azman A, Mahat N, Wahab R, Ahmad W, Puspanadan J, Huri M, et al. Box-Behnken design optimisation of a green novel nanobio-based reagent for rapid visualisation of latent fingerprints on wet, non-porous substrates. Biotechnol Lett. 2021 Apr 1;43(4):881–98.

69. Prlainović NŽ, Bezbradica DI, Knežević-Jugović ZD, Stevanović SI, Avramov Ivić ML, Uskoković PS, et al. Adsorption of lipase from Candida rugosa on multi walled carbon nanotubes. Journal of Industrial and Engineering Chemistry. 2013 Jan 25;19(1):279–85.

70. Che Marzuki NH, Mahat NA, Huyop F, Aboul-Enein HY, Wahab RA. Sustainable production of the emulsifier methyl oleate by Candida rugosa lipase nanoconjugates. Food and Bioproducts Processing. 2015 Oct 1;96:211–20.

71. Mohamad NR, Buang NA, Mahat NA, Jamalis J, Huyop F, Aboul-Enein HY, et al. Simple adsorption of Candida rugosa lipase onto multi-walled carbon nanotubes for sustainable production of the flavor ester geranyl propionate. Journal of Industrial and Engineering Chemistry. 2015 Dec 25;32:99–108.

72. Nadar SS, Kelkar RK, Pise P V., Patil NP, Patil SP, Chaubal-Durve NS, et al. The untapped potential of magnetic nanoparticles for forensic investigations: A comprehensive review. Talanta. 2021 Aug 1;230:122297.

73. Wei S, Cui X. Synthesis of gold nanoparticles immobilized on fibrous nano‐silica for latent fingerprints detection. Journal of Porous Materials. 2021 Jan 25;28(3):751–62.

74. Ansari AAA, Aldajani KMM, AlHazaa ANN, Albrithen HAA. Recent progress of fluorescent materials for fingermarks detection in forensic science and anti-counterfeiting. Coord Chem Rev. 2022;462.

75. Shabashini A, Panja SK, Nandi GC. Applications of Carbon Dots (CDs) in Latent Fingerprints Imaging. Chem Asian J. 2021 May 3;16(9):1057–72.

76. Ding L, Peng D, Wang R, Li Q. A user-secure and highly selective enhancement of latent fingerprints by magnetic composite powder based on carbon dot fluorescence. J Alloys Compd. 2021 Mar 5;856:158160.

77. Tasangtong B, Henry CS, Sameenoi Y. Diameter-based inkjet-printed paper devices for formaldehyde analysis in foods. Food Control. 2023;145.

78. Joao AF, Rocha RG, Matias TA, Richter EM, Petruci JFS, Muñoz RAA. 3D-printing in forensic electrochemistry: Atropine determination in beverages using an additively manufactured graphene-polylactic acid electrode. MICROCHEMICAL JOURNAL. 2021;167.

79. Roushani M, Ghanbarzadeh M, Shahdost-Fard F. Fabrication of an electrochemical biodevice for ractopamine detection under a strategy of a double recognition of the aptamer/molecular imprinting polymer. BIOELECTROCHEMISTRY. 2021;138.

80. Jing MY, Zhang H, Li M, Mao Z, Shi XM. Silver nanoparticle-decorated TiO2 nanotube array for solid-phase microextraction and SERS detection of antibiotic residue in milk. SPECTROCHIMICA ACTA PART A-MOLECULAR AND BIOMOLECULAR SPECTROSCOPY. 2021;255.

81. Zhou SY, Xu XX, Wang L, Guo LL, Liu LQ, Kuang H, et al. A fluorescence based immunochromatographic sensor for monitoring chlorpheniramine and its comparison with a gold nanoparticle-based lateral-flow strip. ANALYST. 2021;146(11):3589–98.

82. Suryoprabowo S, Liu LQ, Kuang H, Cui G, Xu CL. Fluorescence based immunochromatographic sensor for rapid and sensitive detection of tadalafil and comparison with a gold lateral flow immunoassay. Food Chem. 2021;342.

83. Teymourian H, Parrilla M, Sempionatto JR, Montiel NF, Barfidokht A, Van Echelpoel R, et al. Wearable Electrochemical Sensors for the Monitoring and Screening of Drugs. ACS Sens. 2020;5(9):2679–700.

84. Ren SF, Zeng JL, Zheng ZX, Shi HQ. Perspective and application of modified electrode material technology in electrochemical voltammetric sensors for analysis and detection of illicit drugs. SENSORS AND ACTUATORS A-PHYSICAL. 2021;329.

85. Moradi R, Khalili NP, Septiani NW, Liu CH, Doustkhah E, Yamauchi Y, et al. Nanoarchitectonics for Abused-Drug Biosensors. SMALL. 2022;18(10).

86. Khorablou Z, Shahdost-fard F, Razmi H, Yola ML, Karimi-Maleh H. Recent advances in developing optical and electrochemical sensors for analysis of methamphetamine: A review. Chemosphere. 2021;278.

87. Han S, Zhang C, Lin S, Sha X, Hasi W. Sensitive and reliable identification of fentanyl citrate in urine and serum using chloride ion-treated paper-based SERS substrate. Spectrochim Acta A Mol Biomol Spectrosc. 2021 Apr 15;251:119463.

88. Truta F, Florea A, Cernat A, Tertis M, Hosu O, de Wael K, et al. Tackling the Problem of Sensing Commonly Abused Drugs Through Nanomaterials and (Bio)Recognition Approaches. Front Chem. 2020;8.

89. De Rycke E, Stove C, Dubruel P, De Saeger S, Beloglazova N. Recent developments in electrochemical detection of illicit drugs in diverse matrices. Biosens Bioelectron. 2020;169.

90. Zanfrognini B, Pigani L, Zanardi C. Recent advances in the direct electrochemical detection of drugs of abuse. JOURNAL OF SOLID STATE ELECTROCHEMISTRY. 2020;24(11–12):2603–16.

91. Klimuntowski M, Alam MM, Singh G, Howlader MMR. Electrochemical Sensing of Cannabinoids in Biofluids: A Noninvasive Tool for Drug Detection. ACS Sens. 2020;5(3):620–36.

92. Ameku WA, Gonçalves JM, Ataide VN, Santos MSF, Gutz IGR, Araki K, et al. Combined Colorimetric and Electrochemical Measurement Paper-Based Device for Chemometric Proof-of-Concept Analysis of Cocaine Samples. ACS Omega. 2021;6(1):594–605.

93. Rocha DS, Duarte LC, Silva-Neto HA, Chagas CLS, Santana MHP, Antoniosi NR, et al. Sandpaper-based electrochemical devices assembled on a reusable 3D-printed holder to detect date rape drug in beverages. Talanta. 2021;232.

94. Ribeiro MFM, Bento F, Ipólito AJ, de Oliveira MF. Development of a Pencil Drawn Paper-based Analytical Device to Detect Lysergic Acid Diethylamide (LSD). J Forensic Sci. 2020;65(6):2121–8.

95. Dias BC, Batista AD, Petruci JFD. mOPTO: A microfluidic paper-based optoelectronic tongue as presumptive tests for the discrimination of alkaloid drugs for forensic purposes. Anal Chim Acta. 2021;1187.

96. Saisahas K, Soleh A, Promsuwan K, Saichanapan J, Phonchai A, Sadiq NSM, et al. Nanocoral-like Polyaniline-Modified Graphene-Based Electrochemical Paper-Based Analytical Device for a Portable Electrochemical Sensor for Xylazine Detection. ACS Omega. 2022;7(15):13913–24.

97. Pholsiri T, Khamcharoen W, Vimolmangkang S, Siangproh W, Chailapakul O. Paper-based electrochemical sensor for simultaneous detection of salivary?9-tetrahydrocannabinol and thiocyanate to differentiate illegal cannabis smokers. SENSORS AND ACTUATORS B-CHEMICAL. 2023;383.

98. Ataide VN, Mendes LF, Gama L, de Araujo WR, Paixao T. Electrochemical paper-based analytical devices: ten years of development. ANALYTICAL METHODS. 2020;12(8):1030–54.

99. Solin K, Vuoriluoto M, Khakalo A, Tammelin T. Cannabis detection with solid sensors and paper-based immunoassays by conjugating antibodies to nanocellulose. Carbohydr Polym. 2023;304.

100. Sha XY, Han S, Zhao H, Li N, Zhang C, Hasi WLJ. A Rapid Detection Method for On-site Screening of Estazolam in Beverages with Au@Ag Core-shell Nanoparticles Paper-based SERS Substrate. ANALYTICAL SCIENCES. 2020;36(6):667–74.

101. Alder R, Hong JM, Chow E, Fang JH, Isa F, Ashford B, et al. Application of Plasma-Printed Paper-Based SERS Substrate for Cocaine Detection. SENSORS. 2021;21(3).

102. Mao K, Yang ZG, Zhang H, Li XQ, Cooper JM. Paper-based nanosensors to evaluate community-wide illicit drug use for wastewater-based epidemiology. Water Res. 2021;189.

103. Kamel S, Khattab TA. Recent Advances in Cellulose-Based Biosensors for Medical Diagnosis. BIOSENSORS-BASEL. 2020;10(6).

104. Madani-Nejad E, Shokrollahi A, Shahdost-Fard F. A smartphone-based colorimetric assay using Au@Ag core-shell nanoparticles as the nanoprobes for visual tracing of fluvoxamine in biofluids as a common suicide drug. SPECTROCHIMICA ACTA PART A-MOLECULAR AND BIOMOLECULAR SPECTROSCOPY. 2023;296.

105. Garcia-Cruz A, Ahmad OS, Alanazi K, Piletska E, Piletsky SA. Generic sensor platform based on electro-responsive molecularly imprinted polymer nanoparticles (e-NanoMIPs). Microsystems & Nanoengineering 2020 6:1. 2020 Oct 19;6(1):1–9.

106. Papadopoulos F, Diamanteas K, Economou A, Kokkinos C. Rapid Drop-Volume Electrochemical Detection of the “Date Rape” Drug Flunitrazepam in Spirits Using a Screen-Printed Sensor in a Dry-Reagent Format. Sensors 2020, Vol 20, Page 5192. 2020 Sep 11;20(18):5192.

107. Dokuzparmak E, Brown K, Dennany L. Electrochemiluminescent screening for methamphetamine metabolites. Analyst. 2021 May 17;146(10):3336–45.

108. Gill A, Hickey B, Zhong W, Hooley R. Selective Sensing of THC and Related Metabolites in Biofluids by Host:Guest Arrays. ChemComm The Royal Society of Chemistry. 2020;

109. Wille SMR, Elliott S. The Future of Analytical and Interpretative Toxicology: Where are We Going and How Do We Get There? J Anal Toxicol. 2021 Sep 1;45(7):619–32.

110. Ahmed SR, Chand R, Kumar S, Mittal N, Srinivasan S, Rajabzadeh AR. Recent biosensing advances in the rapid detection of illicit drugs. TRAC-TRENDS IN ANALYTICAL CHEMISTRY. 2020;131.

111. Mani V, Beduk T, Khushaim W, Ceylan AE, Timur S, Wolfbeis OS, et al. Electrochemical sensors targeting salivary biomarkers: A comprehensive review. TrAC Trends in Analytical Chemistry. 2021 Feb 1;135:116164.

112. Nardo F Di, Chiarello M, Cavalera S, Baggiani C, Anfossi L. Ten Years of Lateral Flow Immunoassay Technique Applications: Trends, Challenges and Future Perspectives. Sensors 2021, Vol 21, Page 5185. 2021 Jul 30;21(15):5185.

113. Marques L, Félix L, Cruz G, Coelho V, Caetano J, Vale A, et al. Neutron and Gamma-Ray Detection System Coupled to a Multirotor for Screening of Shipping Container Cargo. Sensors 2023, Vol 23, Page 329. 2022;23(1):329.

114. Kim G, Lee J, Jeong S, Kim M. Development of a CsI(Tl) scintillator based gamma probe for the identification of nuclear materials in unknown areas. Journal of Instrumentation. 2022;17(03).

115. Kongkaew S, Tubtimtong S, Thavarungkul P, Kanatharana P, Chang KH, Abdullah AFL, et al. A Fabrication of Multichannel Graphite Electrode Using Low-Cost Stencil-Printing Technique. Sensors. 2022;22(8):3034.

116. Musile G, Agard Y, Wang L, De Palo EF, McCord B, Tagliaro F. Paper-based microfluidic devices: On-site tools for crime scene investigation. TRAC-TRENDS IN ANALYTICAL CHEMISTRY. 2021;143.

117. Parungao D, Vandenabeele P, Edwards HGM, Candeias A, Miguel C. Mobile Raman spectroscopy analysis of elephant ivory objects. Journal of Raman Spectroscopy. 2022;

118. Costanzo H, Gooch J, Frascione N. Nanomaterials for optical biosensors in forensic analysis. Talanta. 2023;253.

119. Abdelkader M, Elmanzalawy M, Pauliukaite R. 3-D Electrodes for Electrochemical Sensors: Review in Different Approaches. IEEE Sens J. 2022;22(24):23620–32.

120. Abdulhussein SK, Al-Kazazz FFM, Rheima AM. The Role of Nanomaterials in the Recent Development of Electrochemical Biosensors. PORTUGALIAE ELECTROCHIMICA ACTA. 2022;41(3):211–21.

121. Alves TMR, Deroco PB, Wachholz DJ, Vidotto LHB, Kubota LT. Wireless Wearable Electrochemical Sensors. BRAZILIAN JOURNAL OF ANALYTICAL CHEMISTRY. 2021;8(31):22–50.

122. Alberti G, Zanoni C, Spina S, Magnaghi LR, Biesuz R. Trends in Molecularly Imprinted Polymers (MIPs)-Based Plasmonic Sensors. CHEMOSENSORS. 2023;11(2).

123. Fakayode SO, Lisse C, Medawala W, Brady PN, Bwambok DK, Anum D, et al. Fluorescent chemical sensors: applications in analytical, environmental, forensic, pharmaceutical, biological, and biomedical sample measurement, and clinical diagnosis. Appl Spectrosc Rev. 2023;

124. Batool M, Afzal Z, Junaid HM, Solangi AR, Hassan A. Sulfonamides as Optical Chemosensors. Crit Rev Anal Chem. 2022;

125. Hardy M, Kelleher L, Gomes PD, Buchan E, Chu HOM, Oppenheimer PG. Methods in Raman spectroscopy for saliva studies - a review. Appl Spectrosc Rev. 2022;57(3):177–233.

126. Mandal P, Tewari BS. Progress in surface enhanced Raman scattering molecular sensing: A review. SURFACES AND INTERFACES. 2022;28.

127. Kulkarni MB, Ayachit NH, Aminabhavi TM. Biosensors and Microfluidic Biosensors: From Fabrication to Application. BIOSENSORS-BASEL. 2022;12(7).

128. Geballa-Koukoula A, Ross GMS, Bosman AJ, Zhao Y, Zhou H, Nielen MWF, et al. Best practices and current implementation of emerging smartphone-based (bio)sensors-Part 2: Development, validation, and social impact. TRAC-TRENDS IN ANALYTICAL CHEMISTRY. 2023;161.
